# Hydroxamic Acids Immobilized on Resins (HAIRs): Synthesis of Dual‐Targeting HDAC Inhibitors and HDAC Degraders (PROTACs)

**DOI:** 10.1002/anie.202006725

**Published:** 2020-10-09

**Authors:** Laura Sinatra, Jan J. Bandolik, Martin Roatsch, Melf Sönnichsen, Clara T. Schoeder, Alexandra Hamacher, Andrea Schöler, Arndt Borkhardt, Jens Meiler, Sanil Bhatia, Matthias U. Kassack, Finn K. Hansen

**Affiliations:** ^1^ Institute for Drug Discovery, Medical Faculty Leipzig University Brüderstraße 34 04103 Leipzig Germany; ^2^ Institute of Pharmaceutical and Medicinal Chemistry Heinrich-Heine-Universität Düsseldorf 40225 Düsseldorf Germany; ^3^ Center for Biopharmaceuticals Department of Drug Design and Pharmacology University of Copenhagen Universitetsparken 2 2100 Copenhagen Denmark; ^4^ Department of Pediatric Oncology Hematology and Clinical Immunology Medical Faculty Heinrich Heine University Düsseldorf Moorenstr. 5 40225 Düsseldorf Germany; ^5^ Center for Structural Biology Department of Chemistry Vanderbilt University Nashville TN 37221 USA; ^6^ Pharmaceutical and Cell Biological Chemistry Pharmaceutical Institute University of Bonn An der Immenburg 4 53121 Bonn Germany

**Keywords:** DNA damage, histone deacetylase, multi-target drugs, PROTAC, solid-phase synthesis

## Abstract

Inhibition of more than one cancer‐related pathway by multi‐target agents is an emerging approach in modern anticancer drug discovery. Here, based on the well‐established synergy between histone deacetylase inhibitors (HDACi) and alkylating agents, we present the discovery of a series of alkylating HDACi using a pharmacophore‐linking strategy. For the parallel synthesis of the target compounds, we developed an efficient solid‐phase‐supported protocol using *hydroxamic acids immobilized on resins* (HAIRs) as stable and versatile building blocks for the preparation of functionalized HDACi. The most promising compound, **3 n**, was significantly more active in apoptosis induction, activation of caspase 3/7, and formation of DNA damage (γ‐H2AX) than the sum of the activities of either active principle alone. Furthermore, to demonstrate the utility of our preloaded resins, the HAIR approach was successfully extended to the synthesis of a proof‐of‐concept proteolysis‐targeting chimera (PROTAC), which efficiently degrades histone deacetylases.

## Graphical abstract

**Hydroxamic acids immobilized on resins** (HAIRs) were developed and utilized for the library synthesis of DNA‐alkylating HDAC inhibitors and a proof‐of‐concept HDAC degrader (PROTAC). A hybrid compound based on the pharmacophores of chlorambucil and panobinostat was identified as the most promising chimeric HDAC inhibitor and demonstrated improved anticancer properties compared to the sum of the activities of either pharmacophore alone.

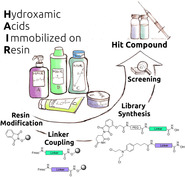

Difficulties in developing new drugs for multifactorial diseases like neurological disorders or cancer have led to rethinking of the “one disease—one target—one drug” paradigm to a “multi‐target drug” concept.^[1]^ Compared to combination therapies using two or more drugs, a single multi‐target molecule has the advantages of no drug‐drug interactions, a more predictable pharmacokinetic profile, and improved patient compliance.[^2^, ^3^]

Histone acetyltransferases (HATs) and histone deacetylases (HDACs) are key enzymes controlling the acetylation level of histones and non‐histone proteins.^[4]^ Due to their repressive effect on gene transcription and their essential influence on drug resistance mechanisms of tumor cells,[^5^, ^6^, ^7^] HDACs are validated drug targets in epigenetic cancer therapy with four inhibitors already approved by the FDA.^[8]^ Typically common HDAC inhibitors (HDACi) comprise a cap group, a linker region and a zinc binding group (ZBG), which is crucial for the chelation of the zinc ion inside the active site tunnel (Figure 1).^[9]^ Fortunately, the cap group can be subjected to various structural modifications providing sufficient scope for hybridization approaches towards HDACi‐based multi‐target drugs.[^10^, ^11^]

**Figure 1:**
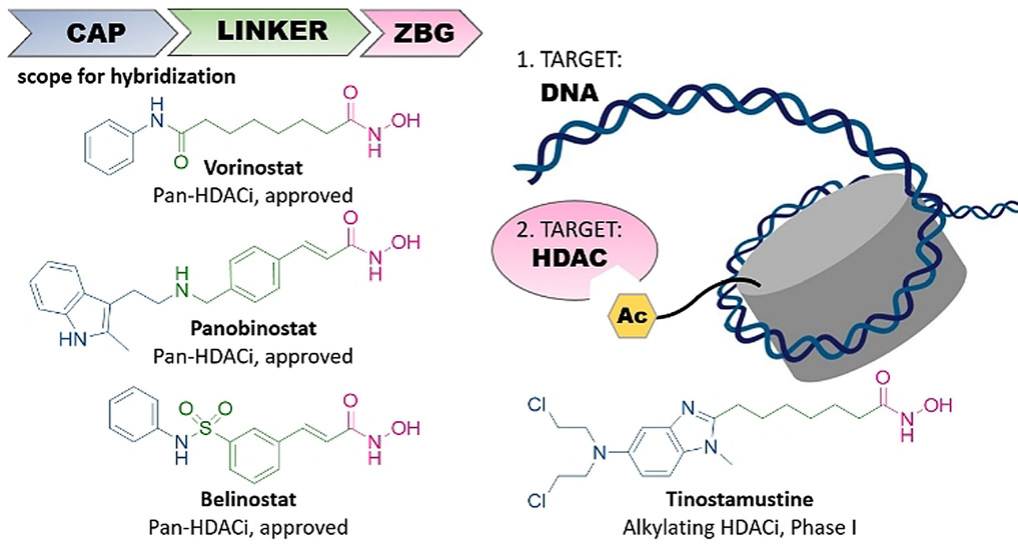
*left*: Selected HDAC inhibitors and their typical design; *right*: intended targets of this work and of the hybrid compound tinostamustine.

Recent preclinical results provide evidence that combinations of alkylating agents and HDACi exhibit efficacy against drug‐resistant glioblastoma multiforme (GBM), the most common and aggressive primary brain tumor,[^12^, ^13^] by increasing the DNA damage of alkylating agents via HDACi‐mediated chromatin relaxation.[^14^, ^15^] Consequently, the first‐in‐class nitrogen mustard‐HDACi hybrid molecule tinostamustine (Figure 1) was developed by fusing the pharmacophores of the DNA‐alkylating drug bendamustine and the HDACi vorinostat (SAHA).^[16]^ Tinostamustine demonstrated superior *in vivo* activity compared to bendamustine, temozolomide and radiotherapy indicating that DNA/HDAC dual‐targeting inhibitors could be promising drug candidates for cancer therapy.

Since HDACi‐based multi‐target drugs show great promise in preclinical and early clinical studies,[^10^, ^11^] there is an urgent need for efficient synthetic protocols allowing the synthesis of focused compound libraries with linked or merged pharmacophores. Herein we present the development of a series of preloaded resins for solid‐phase synthesis termed *hydroxamic acids immobilized on resins* (HAIRs). To demonstrate the utility of these HAIRs and to take advantage of the synergism between HDACi and alkylating agents, we successfully prepared a set of HDACi with DNA‐alkylating properties via a fast and straightforward parallel synthesis approach. The scope of the HAIR technology was further extended to the synthesis of a proof‐of‐concept proteolysis‐targeting chimera (PROTAC), that is, a protein degrader.

The preparation of HAIRs **A**–**E** as HDACi precursors is summarized in Scheme 1. Initially, we modified the commercially available 2‐chlorotrityl chloride (2‐CTC) resin with the immobilization of hydroxylamine by treatment of the resin with *N*‐hydroxyphthalimide and triethylamine for 48 h. Next, after deprotection of the Phth‐group using hydrazine hydrate, different Fmoc‐protected HDACi linkers were loaded to the functionalized resin to provide the preloaded resins HAIRs **A**–**E**. For the linker a series of well‐established HDACi linkers such as benzyl, alkyl and cinnamyl was selected. For all synthesized resins, high loadings between 0.81–0.90 mmol g^−1^ were achieved. All prepared HAIRs showed excellent crude purities and reproducible loadings (±5 %). Furthermore, after storage for >6 months the weight and purity of all modified resins were retested after swelling and drying. All HAIRs showed stable weight (±5 %) and high crude purities (>95 % after >6 months of storage) (Table 1).

**Scheme 1:**
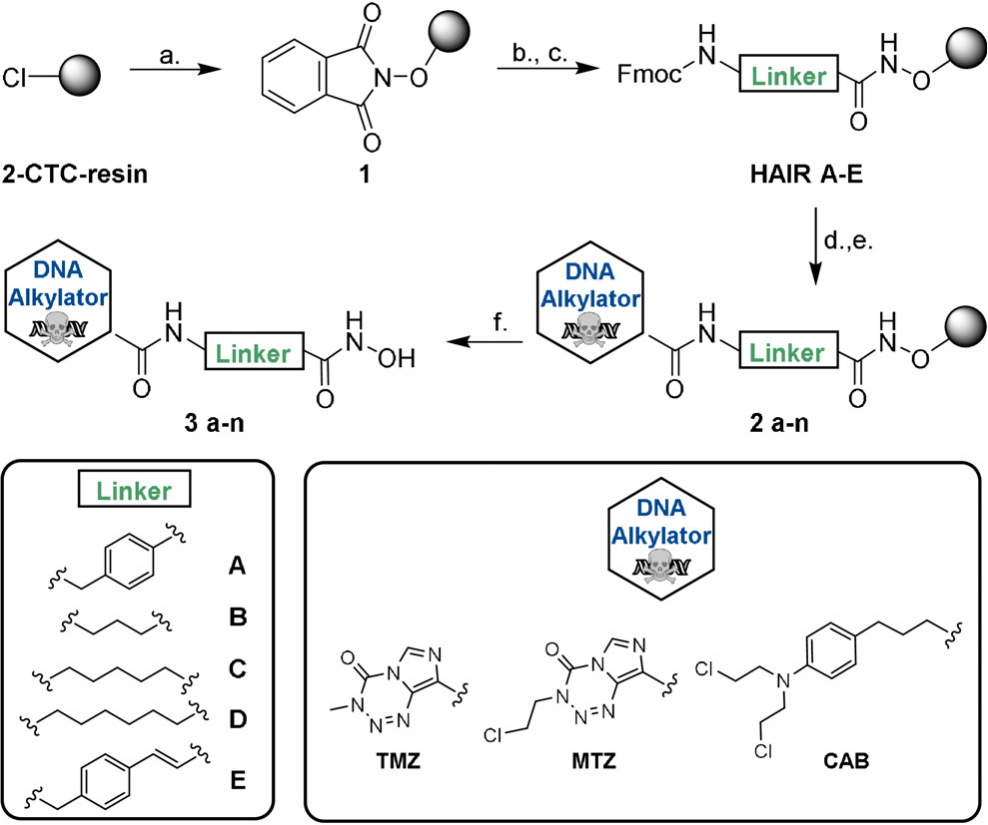
Solid‐phase supported strategy for the parallel synthesis of DNA‐alkylating HDACi hybrid molecules. **a.)** PhthN‐OH (3.5 equiv), Et_3_N (3.5 equiv), DMF, r.t., 48 h. **b.)** 5 % N_2_H_4_⋅H_2_O in MeOH, r.t., 2× 15 min. **c.)** Fmoc‐NH‐Linker‐COOH (2.0 equiv), HATU (2.0 equiv), HOBt⋅H_2_O (2.0 equiv), DIPEA (3.0 equiv), DMF, r.t., 20 h. **d.)** 20 % piperidine in DMF, 2x 5 min. **e.)** Cap‐COOH (3.0 equiv), HATU (3.0 equiv), DIPEA (5.0 equiv), DMF, r.t., 20 h, dark environment. **f.)** 5 % TFA in CH_2_Cl_2_, r.t.,1 h.

**Table 1:** Loadings of synthesized HAIRs and stability after >6 months stored at 4 °C monitored by repetition of the loading and crude purity determination.

Entry	preloaded resin	Loading^[a]^ (mmol g^−1^)	Loading^[a]^ >6 months (mmol g^−1^)	Crude purity^[b]^ >6 months
HAIR **A**	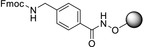	0.90	0.91	96 %
HAIR **B**	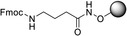	0.96	0.97	95 %
HAIR **C**	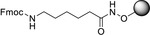	0.87	0.90	96 %
HAIR **D**	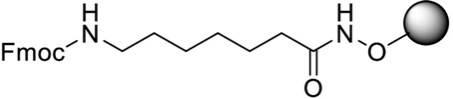	0.81	0.83	96 %
HAIR **E**	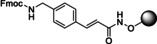	0.87	0.85	95 %

[a] Loadings were photometrically determined at 300 nm after deprotection of the Fmoc‐group using 20 % piperidine in DMF. [b] Crude purities were analyzed by HPLC after test cleavage with 5 % TFA in dichloromethane for 1 h.

After establishing the preloaded HAIRs **A**–**E**, we utilized them to incorporate the DNA‐alkylating part in the cap group region. Due to the synergistic activity of HDACi with alkylating agents, we chose the DNA‐alkylating drugs temozolomide (TMZ), mitozolomide (MTZ), and chlorambucil (CAB) as suitable scaffolds for a hybridization approach. All three examples can be considered as challenging, because TMZ and MTZ are sensitive to base treatment, which leads to ring opening of the imidazotetrazinone ring system, whereas CAB contains a very reactive nitrogen mustard group.[^17^, ^18^] For library synthesis, the Fmoc protecting group was removed and each linker was coupled with the three chosen alkylators in a concentration range of 0.5–1.0 m. Using a parallel synthesis strategy, we prepared a series of compounds combining each linker with each alkylator yielding a library comprising 15 hybrid molecules. This approach provided the target compounds in total yields of up to 83 %. Purification by preparative HPLC afforded all compounds in >95 % purity. Thus, only one purification step at the very end of the synthesis is required for each inhibitor allowing for rapid and time‐efficient library expansion.

Compounds **3 a**–**n** were tested in a fluorogenic assay for their in vitro inhibitory activity against HDAC1 (class I) and HDAC6 (class IIb). Results of the inhibition assay are shown in Table 2. Compounds containing a cinnamyl (**3 l**–**n**) and hexyl (**3 i**–**k**) linker showed inhibitory activity in the nanomolar range against both HDAC1 and HDAC6, which was comparable to the control compound vorinostat. Notably, inhibitors utilizing a benzyl linker revealed potent and preferential inhibition of HDAC6 with up to 17‐fold selectivity over HDAC1 (see compound **3 a**, Table 2). All compounds with a short propyl linker (**3 d**–**f**) showed very low HDAC inhibition, which suggests that this linker length is too short to chelate the Zn^2+^ ion in the active site. To investigate the anticancer properties of the hybrid HDACi, the antiproliferative effects of **3 a**–**n** were determined in three human cancer lines: the human tongue squamous cell carcinoma cell line Cal27 and the human primary glioblastoma cell lines U87 and U251. Results are summarized in Table 2. Based on data from MTT assays, compound **3 n**, which contains a cinnamyl linker and CAB as cap group, emerged as the most promising compound. **3 n** showed HDAC1 and 6 inhibition in a similar range as vorinostat and 3 to 5.6‐fold weaker inhibition compared to the hybrid compound tinostamustine. Furthermore, **3 n** demonstrated the most potent antiproliferative effect of all hybrid compounds against the three cancer cell lines (IC_50_ (Cal27): 2.68 μM, IC_50_ (U87): 19.8 μM, and IC_50_ (U251): 14.5 μM) and exceeded or was equal to the cytotoxicity of the reference compounds vorinostat, tinostamustine, TMZ, MTZ and CAB in the Cal27 cell line and with the exception of vorinostat and tinostamustine also in the glioblastoma cell lines. Notably, **3 n** showed up to 10‐fold improved antiproliferative effect in comparison to its parent compound CAB. Compounds bearing the cinnamyl linker (**3 l**–**n**) showed the most effective combination of antiproliferative effect as well as HDAC inhibitory activity and were therefore chosen for evaluation of their DNA‐damaging effects. **3 l**–**n** were tested for induction of DNA double strand breaks by a γ‐H2AX assay^[19]^ using cisplatin, vorinostat, TMZ, MTZ, and CAB as controls (Figure 2 A). Cal27 cells were treated with an IC_50_ or fivefold IC_50_ (from MTT assay) for 24 h. Among the cinnamyl linker‐containing compounds **3 l**–**n**, **3 n** caused the largest increase in γ‐H2AX levels in Cal27 cells. Using an IC_50_ from MTT assay, only **3 n** achieved a significant increase in γ‐H2AX formation, whereas **3 l** and **3 m** were not different from control. At fivefold IC_50_, **3 l**–**n** showed DNA damage with **3 n** being significantly more active than **3 l** and **3 m**, Because **3 l** and **3 m** showed only weak DNA damage, they were excluded from further studies.

**Figure 2:**
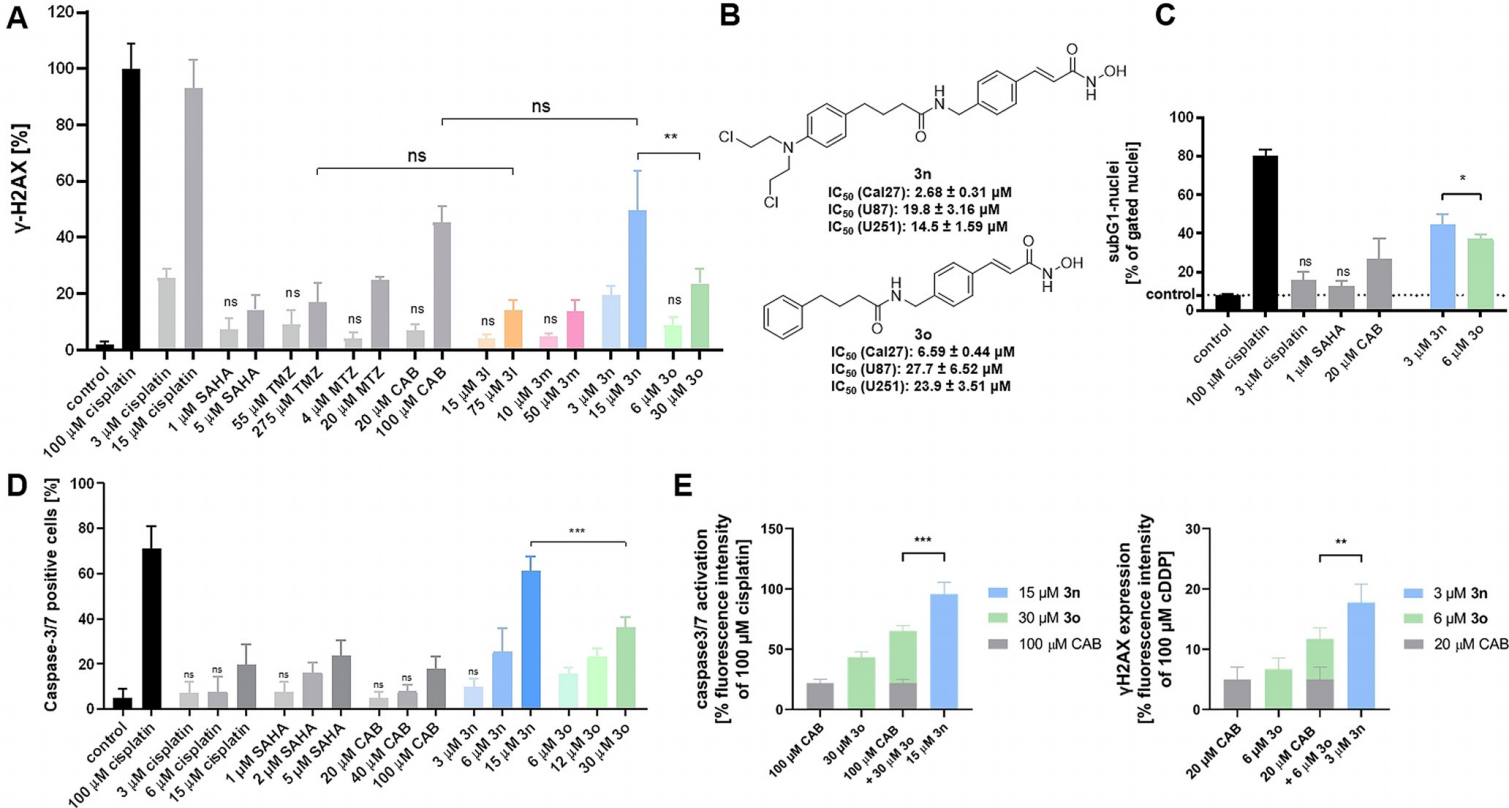
**A**. Compound‐induced formation of γ‐H2AX in Cal27 cells. Cells were treated with an IC_50_ or fivefold IC_50_ (from MTT assay) for 24 h. γ‐H2AX formation was analyzed by immunohistochemistry. 100 μM cisplatin served as positive control and was set as 100 %. “control” is vehicle control. Data are the mean ± SD, *n* ≥3. T‐test was used to analyse for significant differences between compounds and control or as indicated. ns (*p*>0.05); ** (*p* ≤0.01). **B**. Synthesized hit compound **3 n** and its control compound without *N*‐lost‐functionality (**3 o**) and their cytotoxicity. **C**. Induction of apoptosis shown as subG1 nuclei induced by **3 n** and the nitrogen mustard‐free compound **3 o** in Cal27 cells. Cells were treated with indicated compounds and concentrations (MTT‐IC_50_) for 24 h, and sub‐G1 cell fractions were analysed by flow cytometry. 100 μM cisplatin served as positive control for apoptosis induction. “control” is vehicle control. Data are the mean ± SD, *n*=3. T‐test was used to analyze for significant differences between compounds and control or as indicated. ns (*p*>0.05); * (*p* ≤0.05). **D**. Compound‐induced caspase3/7 activation in Cal27 cells. Cells were treated with a single, double, or fivefold IC_50_ (from MTT assay) for 24 h. 100 μM Cisplatin was added as positive control. “control” is vehicle control. Data are the mean ± SD, *n*=3. t‐test was used to analyze for significant differences between compounds and control or as indicated. ns (*p*>0.05); *** (*p* ≤0.001). **E. 3 n** is significantly more effective than the sum of the effects of **3 o** and CAB on caspase3/7 activation (left) and on formation of γH2AX (right). Compounds were used in concentrations reflecting the fivefold IC_50_ (MTT assay). Green and grey bars show sum of the effects of **3 o** and CAB. Light blue bars show the effect of **3 n**. Vehicle control was subtracted. Data are mean ± SD, *n*=3. T‐test was used to analyze for significant differences. ** (*p* ≤0.01); *** (*p* ≤0.001).The analysis was performed as previously described.^[20]^

**Table 2:** *In vitro* inhibition of HDAC1 and 6 by the hybrid inhibitors with different combinations of linkers and cap groups and antiproliferative activity (MTT assay) against the human cancer cell lines Cal27, U87 and U251.

Compound	Linker	Cap Group	Target Inhibition^[a]^ IC_50_ [μM]		Antiproliferative Effect^[b]^ IC_50_ [μM]		
			HDAC1	HDAC6	Cal27	U87	U251
**3 a**	A	TMZ	0.705±0.077	0.035±0.003	16.2±2.86	165±26.1	98.7±10.0
**3 b**	A	MTZ	0.394±0.020	0.023±0.002	16.8±3.62	147±19.7	44.5±8.94
**3 c**	A	CAB	0.518±0.065	0.032±0.003	6.89±1.04	23.9±3.41	22.0±3.09
**3 d**	B	TMZ	35.1±5.32	4.99±0.129	97.1±16.3	323±107	n.e.
**3 e**	B	MTZ	16.2±0.070	2.37±0.086	87.8±9.52	413±77.7	461±89.9
**3 f**	B	CAB	8.21±0.183	4.60±0.082	8.67±1.31	46.1±5.71	20.1±2.41
**3 g**	C	MTZ	0.681±0.015	0.094±0.002	29.0±3.73	262±75.6	140±17.8
**3 h**	C	CAB	0.444±0.053	0.121±0.008	7.50±0.62	43.9±15.3	25.1±3.27
**3 i**	D	TMZ	0.161±0.006	0.031±0.002	10.2±1.54	166±33.6	86.2±13.1
**3 j**	D	MTZ	0.143±0.005	0.038±0.004	9.90±1.24	122±56.0	48.2±7.35
**3 k**	D	CAB	0.244±0.041	0.063±0.001	6.17±0.51	39.2±7.16	27.0±3.55
**3 l**	E	TMZ	0.146±0.008	0.072±0.008	15.4±1.61	141±20.7	97.8±9.07
**3 m**	E	MTZ	0.122±0.009	0.051±0.002	10.1±0.99	84.3±12.4	32.2±3.55
**3 n**	E	CAB	0.151±0.021	0.062±0.001	2.68±0.31	19.8±3.16	14.5±1.59
vorinostat	–	–	0.097±0.012	0.045±0.008	3.26±0.22	6.37±0.51	7.49±0.67
tinostamustine	–	–	0.047±0.003	0.011±0.001	2.94±0.07	8.32±0.55	10.2±0.62
TMZ	–	–	–	‐	56.8±5.05	144±15.6	457±57.3
MTZ	–	–	–	–	3.56±0.29	57.4±9.51	84.5±7.30
CAB	–	–	‐	–	23.7±2.26	86.3±17.5	48.7±11.9
**3 o**	–	–	0.156±0.008	0.066±0.001	6.59±0.44	27.7±6.52	23.9±3.51

[a] *n*≥2, each in duplicate wells. [b] *n*≥3, each in triplicate wells. n.e.: no effect up to 100 μM.

Based on these results, we investigated whether the nitrogen mustard moiety is contributing to the antiproliferative activity and DNA‐damage of **3 n**. We thus synthesized the control compound **3 o** omitting the nitrogen mustard group (see Scheme S1, Supporting Information). The antiproliferative potency of **3 o** was slightly reduced compared to **3 n** (Figure 2 B). The most pronounced reduction in antiproliferative potency was observed in Cal27 cells (**3 n** IC_50_: 2.68 μM vs. **3 o** IC_50_: 6.59 μM). More importantly, **3 n** caused significantly greater DNA damage (γ‐H2AX assay) than **3 o** at a concentration of five times its IC_50_ (Figure 2 A) indicating an additive effect of the nitrogen mustard group. γ‐H2AX formation is a marker for DNA damage, which usually results in induction of apoptosis.^[19]^ Figure 2 C displays apoptosis, shown as subG1 nuclei, induced by **3 n** and the nitrogen mustard‐free control **3 o**. Notably, **3 n** is more potent than **3 o**, cisplatin, vorinostat, and CAB in inducing apoptosis at the respective IC_50_ values. Comparable results were observed in a caspase 3/7 activation‐apoptosis assay demonstrating the superior utility of **3 n** to kill cancer cells via induction of the programmed cell death (Figure 2 D). Notably, although the combination of **3 o** and CAB showed synergism in caspase activation assay as analysed by the method of *Chou‐Talalay* (combination index (CI) values <1), equal concentrations of compound **3 n** demonstrated significantly stronger effects and lower CI values (<0.9) (see Figure S1, Table S1, Supporting Information). In addition, the effect of 15 μM **3 n** was significantly stronger than the sum of the effects of even 100 μM CAB and 30 μM **3 o**, again indicating a superadditive effect of the dual‐targeting compound **3 n** (Figure 2 E, left). The same holds true for results from the γ‐H2AX assay (Figure 2 E, right). The superior antiproliferative activity, apoptosis induction, activation of caspase 3/7, and formation of DNA damage of **3 n** vs. **3 o** could result from an altered HDAC inhibition profile. **3 n** and **3 o** were therefore screened for their inhibitory activity against all class I HDACs and HDAC6. However, **3 n** showed similar or less potent HDAC inhibitory activity than **3 o** (Figure 3 A) highlighting the importance of the DNA‐alkylating feature of the hit compound **3 n** for its anticancer activity. Based on the biochemical HDAC inhibition data, it can be assumed that the nitrogen mustard in *p*‐position of the cap group has little impact on the HDAC inhibitory activity of **3 n**. To test the hypothesis that the DNA alkylating moiety fails to form specific interactions with the HDAC proteins, compounds **3 n** and **3 o** were docked into HDAC6. During the docking study the hydroxamic acid group was restrained in proximity to the zinc ion in the catalytic center, resulting in very similar binding poses for both compounds (Figure 3 B). The addition of the *N*‐lost group did not alter the binding pose of the compounds as it points outwards and only interacts loosely with the protein surface. Taken together, our results demonstrate that the incorporation of a DNA‐alkylating feature in the *p*‐position of the cap group has little impact on the HDAC inhibition and can thus be utilized to enhance the anticancer effects of HDACi.

**Figure 3:**
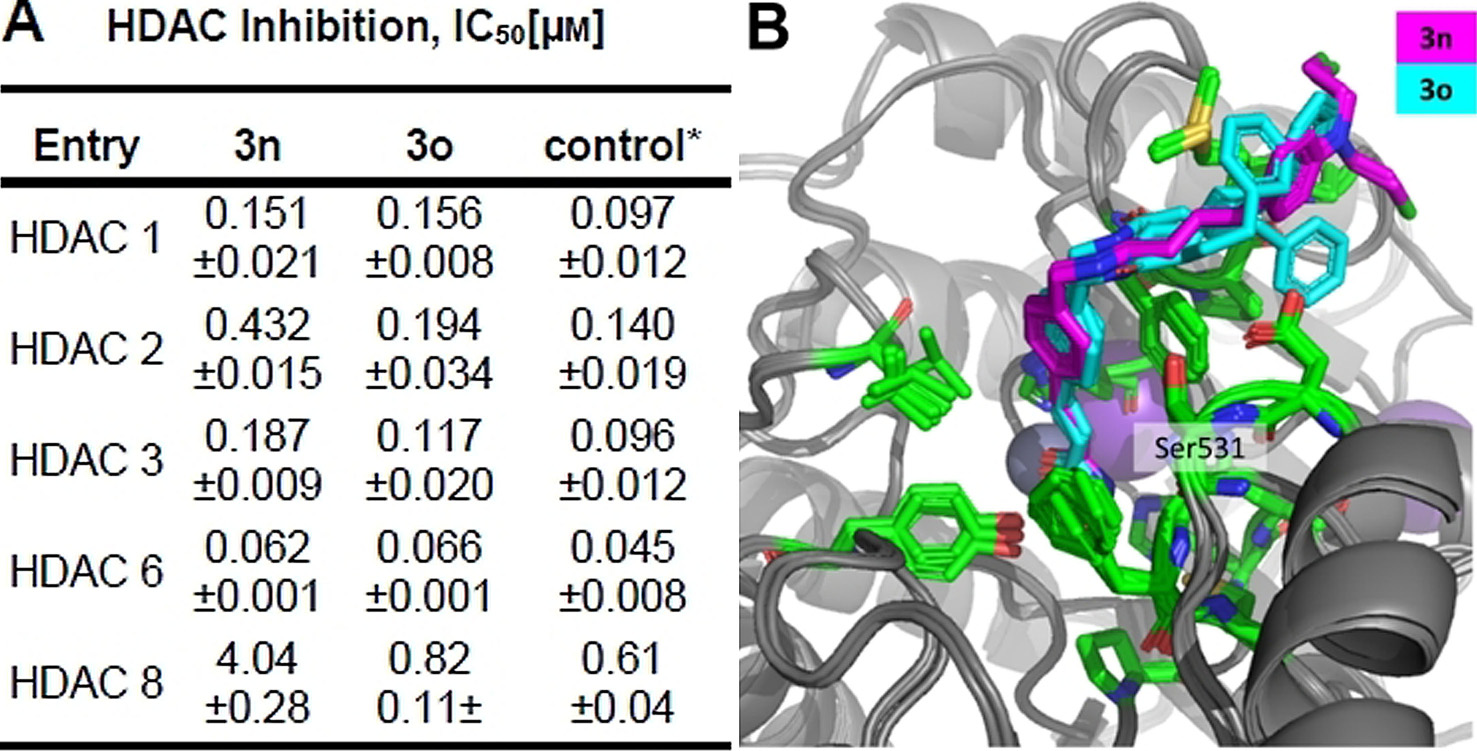
**A**. Inhibition of HDAC 1, 2, 3, 6, and 8 by the hit compound **3 n** and compound **3 o**. *Vorinostat was used as control compound except in the case of HDAC8, where panobinostat was used. *n*≥2, each in duplicate wells. **B**. Docking poses of **3 n** and **3 o** in the human HDAC6 (PDB: 5EDU). Both compounds were individually docked to human HDAC6 using RosettaLigand in iterative rounds of focused docking.

Encouraged by the successful and straightforward parallel synthesis of a focused library of DNA‐alkylating HDACi, our aim was to extend the HAIR approach to a second class of chimeric small molecules: proteolysis‐targeting chimeras (PROTACs). PROTACs are bifunctional small molecules that are able to hijack the cellular protein degradation system by recruiting the protein of interest (POI) to E3 ubiquitin ligases, which leads to polyubiquitinylation of the POI and induction of its proteasomal degradation.[^21^, ^22^] Developments in PROTAC technology give new opportunities to address and study epigenetic targets such as HDACs, with already a few HDAC PROTACs reported recently.[^23^, ^24^, ^25^, ^26^] However, due to their high molecular weight and bifunctional nature, the synthesis of HDAC PROTACs is usually cumbersome and involves multi‐step protocols.^[26]^ Consequently, we undertook the first reported solid‐phase synthesis of an HDAC degrader. HAIR **D** was chosen as a suitable starting point for the preparation of the proof‐of‐concept HDAC PROTAC **4**. Iterative cycles of Fmoc deprotection and amide coupling allowed to introduce the HDAC cap, PROTAC linker, and thalidomide‐based ubiquitin E3 ligase ligand in a modular fashion (see Scheme S2, Supporting Information for synthetic details) to generate PROTAC **4** (Figure 4 A). This straightforward and rapid HAIR‐supported synthesis provided **4** in an excellent crude purity of 91 % (Figure 4 B) and >95 % after purification. Biochemical HDAC inhibition assays highlighted **4** as a potent pan‐HDAC inhibitor (Figure 4 A). Pleasingly, PROTAC **4** turned out to be an efficient HDAC degrader. Western blot experiments using the AML cell line HL60 confirmed that **4** was able to degrade especially HDAC6 and also HDAC1 in a concentration dependent manner (Figure 4 C). Furthermore, the treatment of HL60 cells with **4** led to a significant hyperacetylation of histone H3 (a marker of reduced HDAC1‐3 activity) and α‐tubulin (a marker of reduced HDAC6 activity) (Figure 4 C). Thus, these results clearly confirm that PROTAC **4** is an efficient HDAC degrader, which is suitable for the *chemical knock‐down* of histone deacetylases.

**Figure 4:**
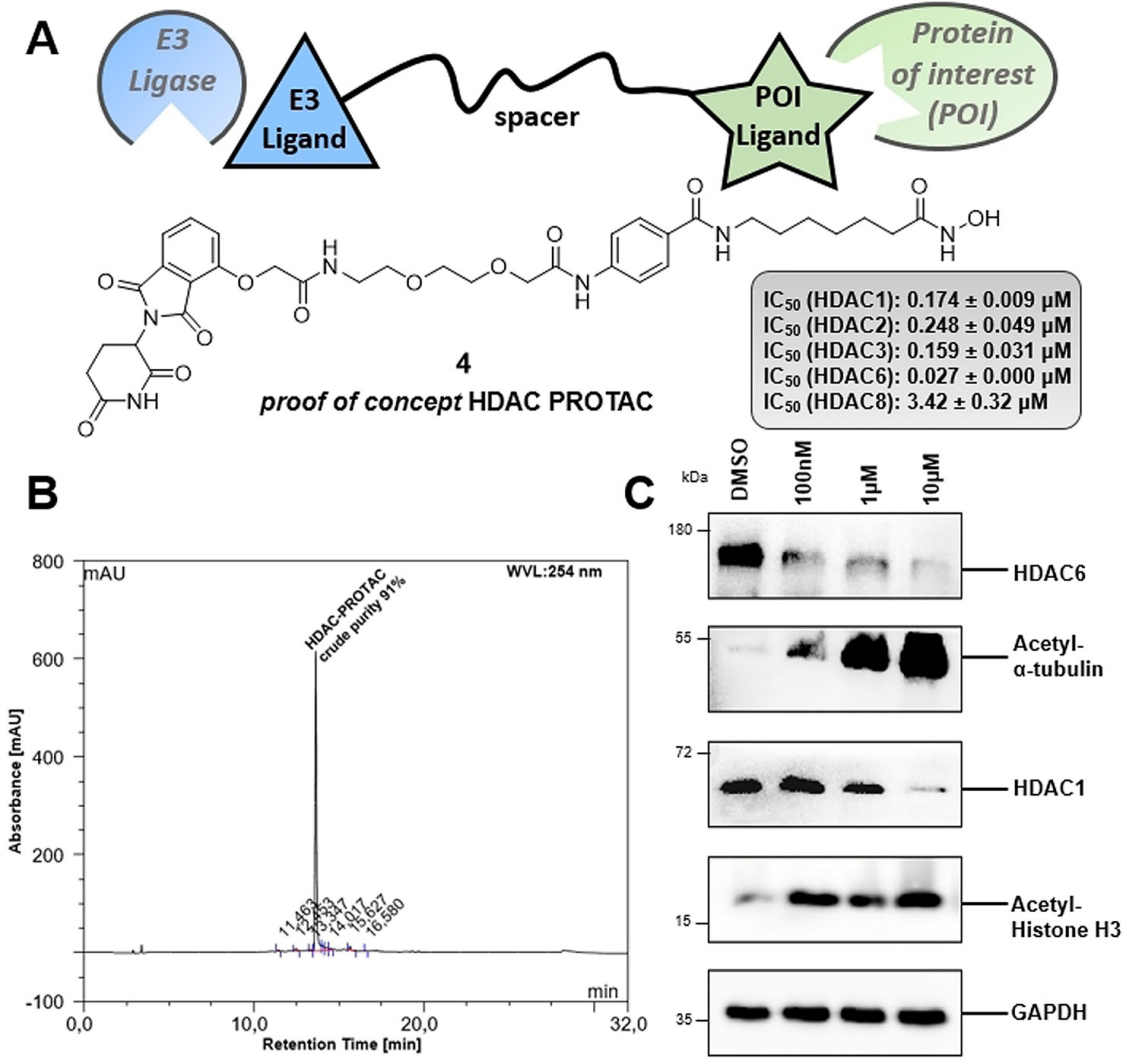
**A**. Synthesized proof of concept HDAC degrader **4** and inhibitory activities against HDAC1, 2, 3, 6, and 8. **B**. HPLC‐chromatogram of the test cleavage of crude proof of concept PROTAC **4** (cleavage mix: 5 % TFA in CH_2_Cl_2_, 1 h, r.t.). **C**. Immunoblot analysis of cell lysate after HL‐60 cells were treated with **4** at various concentrations (0.1 μM, 1 μM and 10 μM).

In conclusion, we have developed an efficient solid‐phase synthesis protocol using *hydroxamic acids immobilized on resins* (HAIRs) to prepare novel dual‐target epigenetic‐cytotoxic compounds. The combination of potent class I/ HDAC6 inhibition with established alkylating agents gave a series of active compounds, among which **3 n** (derived from panobinostat and chlorambucil) showed the highest antiproliferative activity. **3 n** was significantly more active in apoptosis induction, activation of caspase 3/7, and formation of DNA damage (γ‐H2AX) than the sum of the activities of either control compounds alone, that is, chlorambucil and compound **3 o**, an analogue of **3 n** missing the nitrogen mustard. Thus, the combination of an HDACi and a DNA alkylating agent in **3 n** indicates a superadditive effect. Finally, the HAIR technology was applied to synthesize the proof of concept HDAC degrader **4**. Indeed our proof of concept HDAC PROTAC showed efficient degradation of HDACs. The HAIR methodology is thus a versatile method for synthesis of HDACi‐based chimeric small molecules.

## Conflict of interest

The authors declare no conflict of interest.

## Supporting information

As a service to our authors and readers, this journal provides supporting information supplied by the authors. Such materials are peer reviewed and may be re‐organized for online delivery, but are not copy‐edited or typeset. Technical support issues arising from supporting information (other than missing files) should be addressed to the authors.

Supplementary
