# Supplementary material for: Hydroxamic Acids Immobilized on Resins (HAIRs): Synthesis of Dual‐Targeting HDAC Inhibitors and HDAC Degraders (PROTACs)
Source: Angew Chem Int Ed Engl. 2020 Oct 9;59(50):22494–9. doi: 10.1002/anie.202006725 (PMC7756583; doi:10.1002/anie.202006725)
Supplement: Supplementary file 1 — Supplementary [file ANIE-59-22494-s001.pdf]

Angewandte Chemie
Eine Zeitschrift der Gesellschaft Deutscher Chemiker

Supporting Information

Hydroxamic Acids Immobilized on Resins (HAIRs): Synthesis of Dual-Targeting HDAC Inhibitors and HDAC Degraders (PROTACs)

Laura Sinatra, Jan J. Bandolik, Martin Roatsch, Melf Sönnichsen, Clara T. Schoeder, Alexandra Hamacher, Andrea Schöler, Arndt Borkhardt, Jens Meiler, Sanil Bhatia, Matthias U. Kassack, and Finn K. Hansen**

anie_202006725_sm_miscellaneous_information.pdf

Table of Contents

Abbreviations **2**
1 Supplementary tables, schemes and figures **4**
2 Chemistry **6**
2.1 General Remarks 6
2.2 General Procedures 7
2.3 Synthesis 8
Building block synthesis 8
HAIR synthesis 11
Library Synthesis 12
3 Biological Evaluation **38**
3.1 Reagents 38
3.2 Cell Lines and Cell Culture 38
3.3 MTT Cell Viability Assay 38
3.4 γ-H2AX Expression DNA Damage Assay 38
3.5 Measurement of Apoptotic Nuclei 38
3.6 Caspase 3/7 Activation Assay 38
3.7 Immunoblotting 39
3.8 Data Analysis 39
3.9 In-vitro human HDAC1/2/3/6 assay 39
3.10 In-vitro human HDAC8 assay 39
4. Docking Studies **40**
References **42**

Abbreviations

°C	degrees Celsius
Å	angstrom
aq.	aqueous
BSA	bovine serum albumin
c	concentration
calcd	calculated
d	doublet (spectral)
Da	dalton
DNA	deoxyribonucleic acid
DIPEA	*N*,*N*-Diisopropylethylamine
DMEM	Dulbecco's Modified Eagle Medium
DMF	*N,N*-Dimethylformamide
DMSO	dimethyl sulfoxide
EDTA	2,2',2'',2'''-(Ethane-1,2-diyldinitrilo)tetraacetic acid
equiv.	equivalent
ESI	electrospray ionization (mass spectrometry)
et al.	and others
Fmoc	fluorenylmethoxycarbonyl
h	hour
H	histone
HAIR	hydroxamic acid immobilized on resin
HATU	[*O*-(7-Azabenzotriazol-1-yl)-*N*,*N*,*N′*,*N′*-tetramethyluronium-hexafluorophosphate]
HDAC	histone deacetylase
HOBt	1-Hydroxybenzotriazole
HPLC	high performance liquid chromatography
HRMS	high-resolution mass spectrometry
Hz	hertz
IR	infrared spectroscopy
J	coupling constant (NMR spectrometry)
K	kelvin
lit	literature
M	molar (moles per liter)
m	multiplet (spectral)
m/z	mass-to-charge ratio
M+/M-	molecular ion
max.	maximum
MeOH	methanol
min	minutes
mL	milliliter
mm	millimeter
MS	mass spectrometry
MTT	3-(4,5-dimethylthiazol-2-yl)-2,5-diphenyltetrazolium bromide
nm	nanometer
NMR	nuclear magnetic resonance

PBS	phosphate-buffered saline
PDB	protein data bank
PE	polyethylene
PI	propidium iodide
PP	polypropylene
ppm	parts per million
PROTAC	protolysis targeting chimera
q	quartet (spectral)
QVD	5-(2,6-Difluorophenoxy)-3-[[3-methyl-1-oxo-2-[(2-quinolinylcarbonyl)amino]butyl]amino]-4-oxo-pentanoic acid hydrate
RIPA	Radioimmunoprecipitation assay
rpm	revolutions per minute
s	singlet (spectral)
sat.	saturated
SDS	sodium dodecyl sulfate
t	triplet (spectral)
TFA	trifluoroacetic acid
TLC	thin-layer chromatography
TNBS	2,4,6-trinitrobenzenesulfonic acid
TOF	time-of-flight (mass spectrometry)
t_R	retention time
TRIS	Tris(hydroxymethyl)-aminomethane
UV-vis	ultraviolet-visible
v/v	volume per volume
ZMAL	*N*-(4-Methyl-7-aminocoumarinyl)-*N*α-(t-butoxycarbonyl)-*N*ω-acetyllysineamide
δ	NMR chemical shift in parts per million downfield from a standard (tetramethylsilane)
ε	molar extinction coefficient

1 Supplementary tables, schemes and figures

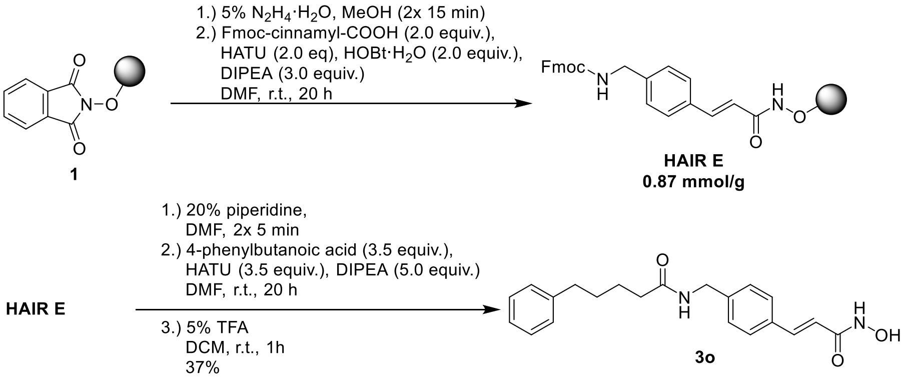

Scheme S1: Solid Phase supported Synthesis of control compound **3o** using HAIR E.

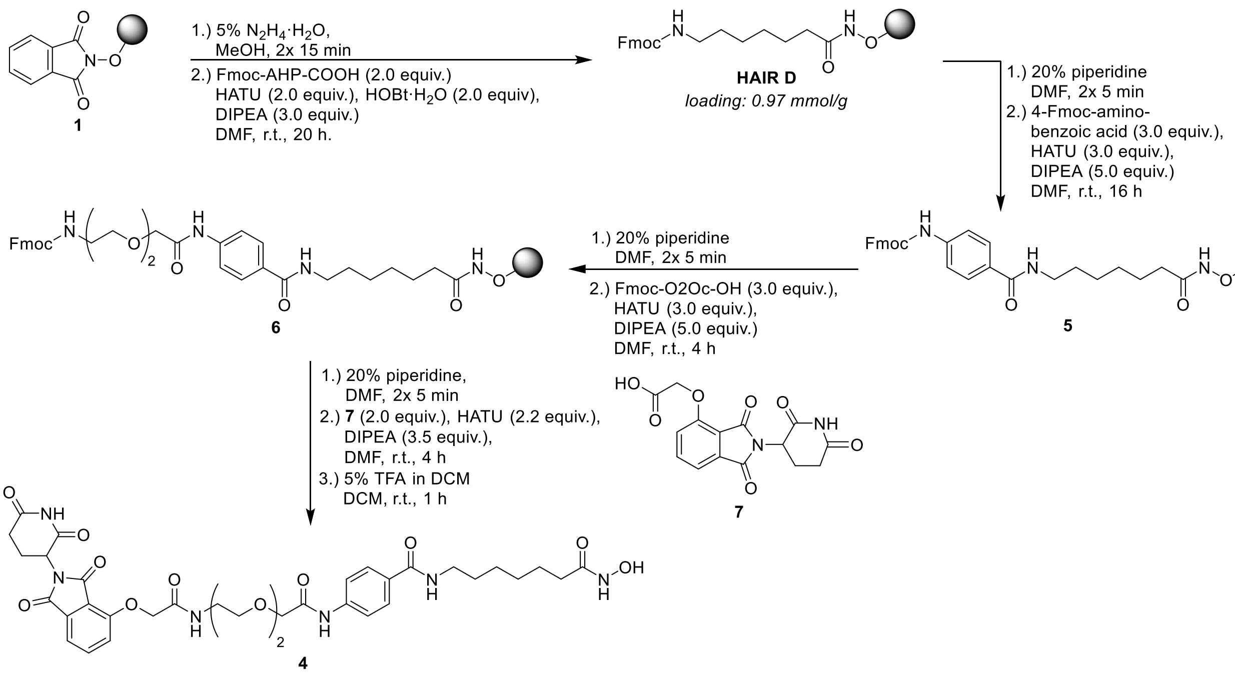

Scheme S2: Synthesis of proof of concept HDAC-PROTAC using solid phase supported HAIR approach. **7** was synthesized according to literature.[1]

Table S1: Data shown are combination indices (CI) calculated using CompuSyn 1.0 based on the Chou-Talalay method [2, 3]. CI > 1.0 indicates antagonism, CI = 1 indicates an additive effect, and CI < 1.0 indicates synergism. Cal27 cells were incubated with the drugs indicated for 24 h and caspase-3/7-positive cells were analyzed. The single drug effects of **3o** and CAB were compared to the effect of coincubation of "**3o** + CAB" and to the effect of **3n**. Values are the mean of three experiments. SD is < 10 % of the mean.

[3o], µM	[CAB], µM	CI-Values	
		3o + CAB	3n
6	6	0,93	0,92
9	9	0,90	0,69
12	12	0,96	0,70

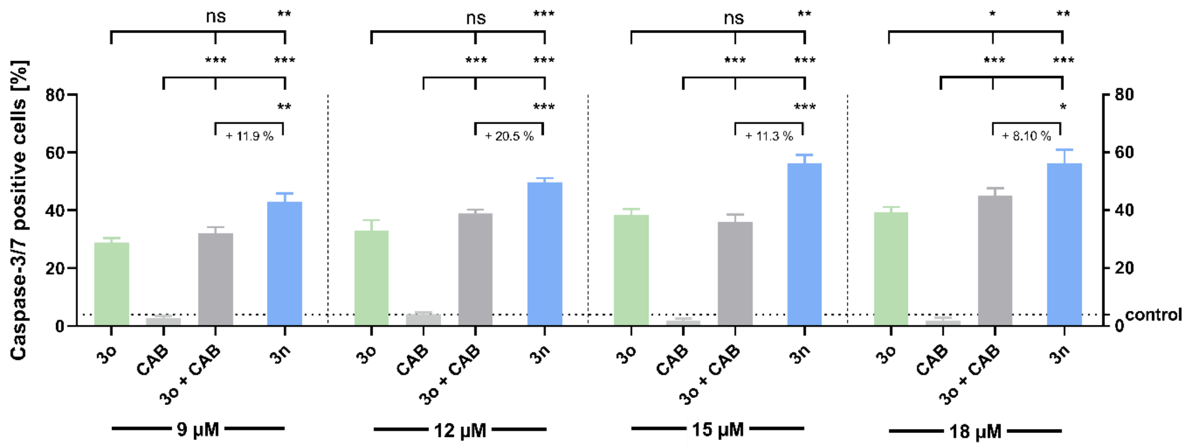

Figure S1: Compound-induced caspase-3/7 activation in Cal27 cells. Cells were treated with indicated concentrations for 24 h. 100 µM Cisplatin was added as positive control (data not shown). "control" is vehicle control (and showed 3.96 % caspase-3/7 positive cells). t-test was used to analyse for significant differences between **3o** or CAB single treatment and coincubation of **3o** and CAB or **3n** and the comparison of **3o** and CAB coincubation and **3n** single treatment as indicated. The increase in caspase-3/7 activation by **3n** single treatment over coincubation of **3o** and CAB is shown as percentage points below the respective bracket. Data are the mean ± SD, n = 3. ns ($p > 0.05$); * ($p \leq 0.05$); ** ($p \leq 0.01$); *** ($p \leq 0.001$).

2 Chemistry

2.1 General Remarks

Materials and Experimental Procedures

All chemicals were obtained from commercial suppliers (Sigma-Aldrich, Acros Organics, Carbolution Chemicals, Iris Biotech, TCI Chemicals) and used as purchased without further purification. 2-((2-(2,6-Dioxopiperidin-3-yl)-1,3-dioxoisoindolin-4-yl)oxy)acetic acid (**7**) was synthesized according to literature known procedures.[1] Solvents with technical grade were distilled prior to use. Acetonitrile in HPLC-grade quality (HiPerSolv CHROMANORM, *VWR*) was used for all HPLC purposes. Water was purified with a Milli-Q Simplicity 185 Water Purification System (Merck Millipore). All reactions with water- and/or air-sensitive starting materials were carried out in pre-dried glass wares under Argon atmosphere utilizing standard *Schlenk* techniques. All solid phase reactions were carried out in PP-reactors with PE frits (sizes: 2/5/10/20 mL, pore size 23 µm, *MultiSynTech GmbH*) and a 2-chlorotrityl chloride resin (200-400 mesh, 1.60 mmol/g, Iris Biotech) was used. Thin-layer chromatography (TLC) was carried out on prefabricated plates (silica gel 60, F254 with fluorescence indicator, *Merck*). Components were visualized by irradiation with ultraviolet light (254 nm, 366 nm) or by staining in potassium permanganate dip or ninhydrin dip followed by careful heating (~300 °C). Column Chromatography was either carried out on silica gel (NORMASIL 60®, 40-63 µm, *VWR*) or on a *Teledyne ISCO* Combi Flash NEXTGEN 300+ using prepacked silica columns (Redisep® normal phase, 35 to 70 microns, 230 to 400 mesh, 60 Å in the sizes 12 g or 24 g, *Teledyne ISCO*).

Figure 1 was partially created with BioRender.com.

Nuclear Magnetic Resonance Spectroscopy (NMR)

Proton (^{1}H) and carbon (^{13}C) NMR spectra were recorded either on a *Bruker* Avance III HD 400 MHz at a frequency of 400 MHz (^{1}H) and 100 MHz (^{13}C) or *Varian/Agilent* Mecury-plus-400 at a frequency of 400 MHz (^{1}H) and 100 MHz (^{13}C) or a *Varian/Agilent* Mecury-plus-300 at a frequency of 300 MHz (^{1}H) and 75 MHz (^{13}C). The residual solvent signal ($CDCl_3$: ^{1}H NMR: 7.26 ppm, ^{13}C NMR: 77.1 ppm, DMSO-d_6: ^{1}H NMR: 2.50 ppm, ^{13}C NMR: 39.52 ppm) was used for calibration referred to tetramethylsilane. As solvents deuterated chloroform ($CDCl_3$), deuterated methanol (MeOD) and deuterated dimethyl sulfoxide (DMSO-d_6) were used. The chemical shifts are given in parts per million (ppm). The multiplicity of each signal is reported as singlet (s), doublet (d), triplet (t), quartet (q), multiplet (m) or combinations thereof. Multiplicities are reported as they were measured, and they might disagree with the expected multiplicity of a signal.

Mass Spectrometry

High resolution electrospray ionisation mass spectra (HR-ESI-MS) were acquired either with a *Bruker Daltonik GmbH* micrOTOF coupled to a an *LC Packings* Ultimate HPLC system and controlled by micrOTOFControl3.4 and HyStar 3.2-LC/MS or with a *Bruker Daltonik GmbH* ESI-qTOF Impact II coupled to a *Dionex* UltiMate^{TM} 3000 UHPLC system and controlled by micrOTOFControl 4.0 and HyStar 3.2-LC/MS.

High Performance Liquid Chromatography (HPLC)

For analytical purposes either a *Thermo Fisher Scientific* UltiMate^{TM} 3000 UHPLC system or a *Gynkotek* Gina 50 HPLC system (Detector: Gynkotek UVD340U, Pump: Dionex P680 HPLC pump, column oven: Dionex STH 585) with a Nucleodur 5 u C18 100 Å (250 x 4.6 mm, Macherey Nagel) column were used. In the process a flow rate of 1 mL/min and a temperature of 25° C were set. For preparative purposes a *Varian* ProStar system with either a Jupiter 5 u C18 100 Å-column (250 x 10 mm, Phenomenex) with 4 mL/min or a Nucleodur 5 u C18 HTec (150 x 32 mm, Macherey Nagel) column with 15 mL/min were used. Detection was implemented by UV absorption measurement at a wavelength of λ = 220 nm and λ =254 nm. Bidest. H_2O (A) and MeCN (B) were used as eluents with an addition of 0.1% TFA for eluent A. For analytical as well as preparative purposes after column equilibration for 5 min a linear gradient from 5% A to 95% B in 15 min followed by an isocratic regime of 95% B for 5 min was used.

UV-VIS and Infrared Spectroscopy (IR)

Loading determinations were performed on a *Shimadzu* UV-160A spectrometer at room temperature. All measurements were performed in a 3500 µL quartz cuvette (100-QS, Hellma Analytics) with a path lengths of 10 mm. Infrared spectroscopy measurements were performed on a PerkinElmer SpectrumTwo FT-IR spectrometer at room temperature.

Determination of the Loading

A small part of the different HAIRs (~5 mg) was weighed and treated with 500 µL of the deprotection solution (20% piperidine in DMF) for 5 min. The solution was collected and the procedure was repeated once. The concentration of the cleaved Fmoc-group was determined photometrically ($\varepsilon_{300\ nm}$(dibenzofulvene)= 7800 $M^{-1}cm^{-1}$). With the concentration of the cleaved dibenzofulvene determined by using Lambert-Beer law and the mass of the resin the loading could be determined.

$$A = \varepsilon \cdot c \cdot l$$

where *A* = absorbance; ε = molar extinction coefficient; *c* = concentration; *l* = optical path length.

2.2 General Procedures

General Procedure A

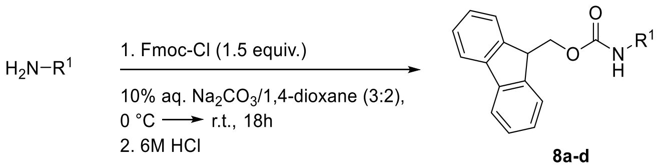

The amounts of reagents and solvents used in the following synthesis protocol correspond to 6.00 – 8.00 mmol scale. To a cooled (0 °C) solution of the appropriate amine (1.00 equiv.) in 10% aq. Na_2CO_3/1,4-dioxane (3:2, 20mL/mmol) was added Fmoc-Cl (1.50 equiv.) in five portions over a period of 20 min. The mixture was then allowed to warm to room temperature and stirred for 18 h. Upon completion of the reaction, the reaction solution was diluted with dest. H_2O (50 mL) and washed with Et_2O (3 x 150 mL). Subsequently the aqueous phase was acidified to pH 1 with 6 M HCl. Filtration of the resulting precipitate, followed by washing with dest. H_2O (200 mL) and drying *in vacuo* afforded the desired products **8a-d**.

General Procedure B

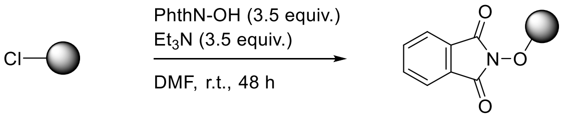

The amounts of reagents and solvents used in the following synthesis protocol correspond to a 3.00 – 4.00 mmol scale. This reaction step was carried out based on a procedure of Khan *et. al.*[4] After resin swelling for 30 min in DMF, a solution of *N*-hydroxyphthalimide (3.50 equiv) and Et_3N (3.50 equiv.) in DMF (1.5 mL/g resin) was added to the resin and reacted for 48 h. Afterwards the resin was washed with DMF (10 x 5 mL) and CH_2Cl_2 (10 x 5 mL). Capping of the modified resin was performed by treatment with capping solution (10 mL, CH_2Cl_2/MeOH/DIPEA, 80:15:5) two times for 15 min. Subsequently, the resin was washed with DMF (5 x 5 mL) and CH_2Cl_2 (10 x 5 mL) and dried *in vacuo* to afford the modified resin **1**.

General Procedure C

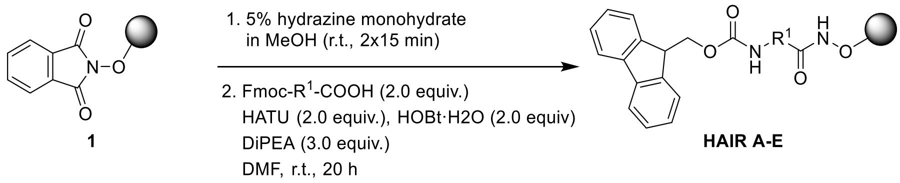

The amounts of reagents and solvents used in the following synthesis protocol correspond to a 1.50 – 2.00 mmol scale. After swelling of the modified resin **1** (estimated loading 1.50 mmol/g) for 30 min in DMF, the resin was washed with MeOH (3 x 5 mL). The phthaloyl protecting group was removed by treatment with 5% hydrazine monohydrate in MeOH for 15 min (2 x 5 mL). Afterwards the resin was washed with DMF (5 x 5 mL), MeOH (5 x 5 mL), CH_2Cl_2 (5 x 5 mL) and DMF (5 x 5 mL). For the subsequent amide coupling reaction a solution of the respective acid (2.00 equiv.), HATU (2.00 equiv.), HOBt·H_2O (2.00 equiv.) and DIPEA (3.00 equiv.) in DMF (1 mL/mmol acid) was agitated for 5 min and then added to the resin. The amide coupling was performed for 20 h at room temperature. Afterwards the resin was washed with DMF (5 x 5 mL) and CH_2Cl_2 (5 x 5 mL). Completion of the reaction was monitored via TNBS-test. The preloaded resins **HAIR A-E** were dried *in vacuo* and stored at 4 °C. Determination of the loading capacity was conducted photometrically.

General Procedure D

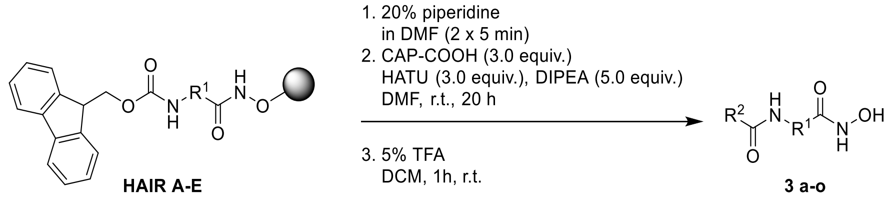

The amounts of reagents and solvents used in the following synthesis protocol correspond to 0.30 – 0.50 mmol scale. After swelling of the appropriate **HAIR** in DMF for 30 min, Fmoc deprotection was performed by treatment with 20% piperidine in DMF for 5 min (2 x 3 mL). Afterwards the resin was washed with DMF (5 x 3 mL), CH_2Cl_2 (5 x 3 mL) and DMF (5 x 3 mL). For the subsequent amide coupling reaction a solution of the respective acid (3.00 equiv.), HATU (3.00 equiv.), and DIPEA (5.00 equiv.) in DMF (1 mL/mmol acid) was agitated for 5 min and then added to the resin. The amide coupling was performed in dark environment for 20 h at room temperature. Afterwards the resin was washed with DMF (5 x 3 mL) and CH_2Cl_2 (5 x 3 mL). Completion of the reaction was monitored via TNBS-test on a few resin beads. The resin was dried *in vacuo* followed by the cleavage of the crude products from the resin by treatment with cleavage solution (5% TFA in CH_2Cl_2, 1 mL/40 mg resin) for 1 h at room temperature. The filtrates were concentrated *in vacuo* and the crude products **3a-o** were purified by preparative HPLC. Lyophilization of the respective fractions yielded the desired products **3a-o** in >95% purity.

2.3 Synthesis

Building block synthesis

*4-((((9H-Fluoren-9-yl)methoxy)carbonyl)amino)butanoic acid (**8a**)*

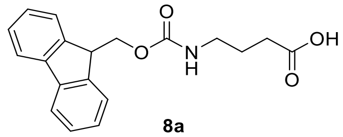

8a

Synthesized from 4-aminobutanoic acid (2.00 g, 19.4 mmol, 1.00 equiv.) and 9-fluorenylmethoxycarbonyl chloride (7.53 g, 29.1 mmol, 1.50 equiv.) according to general procedure A. Filtration and drying *in vacuo* afforded **8a** as a white solid (5.04 g, 15.5 mmol, 80%). **^{1}H-NMR** (400 MHz, MeOD): δ= 7.79 (d, *J* = 7.6 Hz, 2H), 7.65 (d, *J* = 7.5 Hz, 2H), 7.39 (t, *J* = 7.4 Hz, 2H), 7.31 (t, *J* = 7.4 Hz, 2H), 4.35 (d, *J* = 6.8 Hz, 2H), 4.20 (t, *J* = 6.9 Hz, 1H), 3.15 (t, *J* = 6.8 Hz, 2H), 2.31 (t, *J* = 7.5 Hz, 2H), 1.84 – 1.69 (m, 2H) ppm, -COO*H* signal could not be detected due to solvent exchange. **HRMS-ESI (*m/z*)**: $[M + Na]^+$ calcd for $C_{19}H_{19}NO_4$: 348.1206; found: 348.1204. The analytical data are in accordance with the literature.[5]

*7-((((9H-Fluoren-9-yl)methoxy)carbonyl)amino)heptanoic acid (**8b**)*

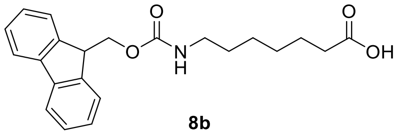

8b

Synthesized from 4-aminoheptanoic acid (1.00 g, 6.90 mmol, 1.00 equiv.) and 9-fluorenylmethoxycarbonyl chloride (2.67 g, 10.3 mmol, 1.50 equiv.) according to general procedure A. Filtration and drying *in vacuo* afforded **8b** as a white solid (2,26 g, 6.14 mmol, 89%). **^{1}H-NMR** (400 MHz, DMSO-d_6): δ= 7.88 (dt, *J* = 7.6, 1.0 Hz, 2H), 7.68 (d, *J* = 7.4 Hz, 2H), 7.41 (td, *J* = 7.5, 1.2 Hz, 2H), 7.32 (td, *J* = 7.4, 1.2 Hz, 2H), 7.24 (t, *J* = 5.7 Hz, 1H), 4.29 (d, *J* = 6.9 Hz, 2H), 4.20 (t, *J* = 6.9 Hz, 1H), 2.98 – 2.92 (m, *J* = 6.6 Hz, 2H), 2.18 (t, *J* = 7.4 Hz, 2H), 1.48 (p, *J* = 7.4 Hz, 2H), 1.38 (p, *J* = 6.9 Hz, 2H), 1.25 (h, *J* = 4.2, 3.1 Hz, 4H) ppm, -COO*H* signal could not be detected due to solvent exchange. **^{13}C-NMR** (101 MHz, DMSO-d_6): δ= 174.6, 156.1, 144.0, 140.8, 127.6, 127.1, 125.2, 121.4, 120.1, 65.2, 46.8, 33.8, 29.2, 28.3, 26.0, 24.5 ppm. **HRMS-ESI (*m/z*)**: $[M - H]^-$ calcd for $C_{22}H_{25}NO_4$: 366.1711, found: 366.1722. **IR**: $\tilde{\nu}$= 3222 (br), 2935 (br), 1743 (vs), 1640 (vs), 1579 (s), 1518 (s), 1455 (vs),1249 (s), 1087 (s), 956 (m), 735 (s) cm^{-1}.

*tert-Butyl (4-bromobenzyl)carbamate (**9**)*

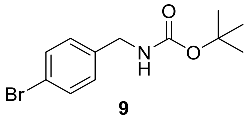

9

To a cooled (0 °C) solution of 4-bromobenzylamine (1.00 g, 5.38 mmol, 1.00 equiv.) and triethylamine (822 µL, 5.91 mmol, 1.10 equiv.) in CH_2Cl_2 (15 mL), a solution of di-*tert*-butyl dicarbonate (1.29 g, 5.91 mmol, 1.10 equiv.) in CH_2Cl_2 (10 mL) was added dropwise. The mixture was then allowed to warm to room temperature and stirred for 18 h. Upon completion of the reaction, the solvent was removed *in vacuo* and the crude product was dissolved in EtOAc (20 mL). The organic phase was acidified to pH 4 with anhydrous citric acid. The organic phase was washed with dest. H_2O (3 x 20 mL) and sat. $NaHCO_3$ (3 x 20 mL). Drying over anhydrous $MgSO_4$ and removal of the solvent *in vacuo* afforded the crude product **9**, which was purified by flash column chromatography (cyclohexane/EtOAc, 4:1) to afford the desired product **9** (1.15 g, 4.04 mmol, 75%). **^{1}H-NMR** (400 MHz, $CDCl_3$): δ= 7.60 – 7.39 (m, 2H), 7.15 (d, *J* = 8.0 Hz, 2H), 4.85 (s, 1H), 4.26 (d, *J* = 6.0 Hz, 2H), 1.45 (s, 9H) ppm. **^{13}C-NMR** (75 MHz, $CDCl_3$): δ= 156.0, 138.2, 131.8, 129.3, 121.3, 58.4, 44.2, 28.5 ppm. **HRMS-ESI (*m/z*)**: $[M + Na]^+$ calcd for $C_{12}H_{16}BrNO_2$: 308.0257, found: 308.0253. **IR**: $\tilde{\nu}$= 3363 (m), 3340 (m), 2976 (m), 2931 (w), 1682 (vs), 1534 (s), 1504 (vs), 1486 (s), 1362 (s), 1281 (m), 1244 (vs), 1157 (vs), 1068 (m), 1047 (vs), 1010 (s), 877 (m), 808 (m), 798 (m), 623 (m), 479 (s) cm^{-1}.

*tert-Butyl (E)-3-(4-(((tert-butoxycarbonyl)amino)methyl)phenyl)acrylate (**10**)*

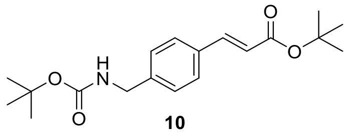

10

To a mixture of tert-butyl (4-bromobenzyl)carbamate (600 mg, 2.10 mmol, 1.00 equiv.), $[PdCl_2(PPh_3)_2]$ (73.5 mg, 0.11 mmol, 5 mol%), LiCl (177 mg, 4.20 mmol, 2.00 equiv.) and DIPEA (714 µL, 4.20 mmol, 2.00 equiv.) in DMF (7 mL) was added *tert*-Butyl acrylate (609 µL, 4.20 mmol, 2.00 equiv.). The reaction was heated up to 100 °C under argon atmosphere for 16 h. Upon completion of the reaction, the solvent was removed *in vacuo* and coevaporated with toluene (3 x 15 mL). The residue war dissolved in EtOAc (20 mL) and washed with 1 M HCl (5 x 20 mL), sat. aq. $NaHCO_3$ (3 x 20 mL) and brine (3 x 20 mL). The organic phase was dried over anhydrous $MgSO_4$ and the solvent was removed under reduced pressure. Purification by flash column chromatography (cyclohexane/EtOAc, 9:1) afforded the desired product **10** as white solid (574 mg, 1.72 mmol, 82%). **^{1}H-NMR** (400 MHz, $CDCl_3$): δ= 7.56 (d, *J* = 16.0 Hz, 1H), 7.46 (d, *J* = 8.0 Hz, 2H), 7.28 (d, *J* = 8.1 Hz, 2H), 6.34 (d, *J* = 16.0 Hz, 1H), 4.88 (s, 1H), 4.32 (d, *J* = 6.0 Hz, 2H), 1.53 (s, 9H), 1.46 (s, 9H) ppm. **^{13}C-NMR** (101 MHz, $CDCl_3$): δ= 166.5, 156.0, 143.2, 141.2, 133.9, 128.4, 127.9, 120.2, 80.7, 79.8, 44.5, 28.5, 28.3 ppm. **HRMS-ESI (*m/z*)**: $[M + Na]^+$ calcd for $C_{19}H_{27}NO_4$: 356.1832, found: 356.1828. **IR**: $\tilde{\nu}$= 3362 (br), 2977 (m), 2930 (m), 1703 (vs), 1513 (br), 1367 (m), 1151 (vs), 845 (br) cm^{-1}.

*(E)-3-(4-(((((9H-Fluoren-9-yl)methoxy)carbonyl)amino)methyl)phenyl)acrylic acid (**8c**)*

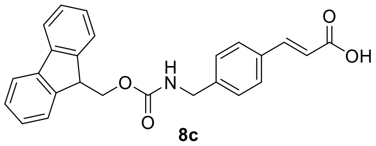

8c

TFA (7.5 mL) was added dropwise to a cooled (0 °C) solution of **10** (511 mg, 1.55 mmol, 1.00 equiv.) in CH_2Cl_2 (22.5 mL). After stirring for 15 min, the solution was allowed to warm to room temperature and stirred for further 2 h. Upon completion of the reaction, the solvent was removed *in vacuo*. The crude product was further reacted with 9-fluorenylmethoxycarbonyl chloride (481mg, 1.86 mmol, 1.20 equiv.) following general procedure A yielding the desired product **8c** as white solid (482 mg, 1.20 mmol, 77%). **^{1}H-NMR** (400 MHz, DMSO-*d*$_6$): δ= 12.39 (s, 1H), 7.89 -7.82 (m, 3H), 7.70 (d, *J* = 7.5 Hz, 2H), 7.63 (d, *J* = 8.0 Hz, 2H), 7.57 (d, *J* = 16.0 Hz, 1H), 7.42 (t, *J* = 7.5 Hz, 2H), 7.33 (t, *J* = 7.4 Hz, 2H), 7.25 (d, *J* = 7.9 Hz, 2H), 6.51 (d, *J* = 16.0 Hz, 1H), 4.36 (d, *J* = 6.8 Hz, 2H), 4.23 (t, *J* = 6.7 Hz, 1H), 4.20 (d, *J* = 6.2 Hz, 2H) ppm. **^{13}C-NMR** (101 MHz, DMSO-*d*$_6$): δ= 167.9, 156.4, 143.9, 143.2, 142.0, 140.8, 133.0, 128.1, 127.6, 127.4, 127.1, 125.2, 120.1, 119.4, 65.3, 46.8, 43.5 ppm. **HRMS-ESI (*m/z*)**: $[M - H]^-$ calcd for $C_{25}H_{21}NO_4$: 398.1398, found:398.1398. **IR**: $\tilde{\nu}$= 3310 (br), 1686 (vs), 31 (m), 1532 (m), 1427 (w), 1254 (s), 1142 (m), 984 (m), 757 (m), 737 (s), 533 (m) cm^{-1}.

*4-((((9H-Fluoren-9-yl)methoxy)carbonyl)amino)benzoic acid (**8d**)*

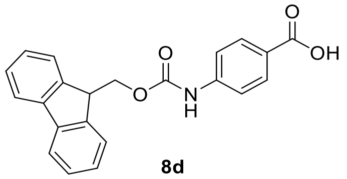

8d

Synthesized from 4-aminobenzoic acid (1.00 g, 7.30mmol, 1.00 equiv.) and 9-fluorenylmethoxycarbonyl chloride (2.83 g, 11.0 mmol, 1.50 equiv.) according to general procedure A. Filtration and drying *in vacuo* afforded the desired product **8d** as white solid (2.32 g, 6.46 mmol, 88%). ^{1}**H-NMR** (400 MHz, DMSO-d_6): δ= 12.67 (s, 1H, broad signal), 10.06 (s, 1H), 7.91 (dt, *J* = 7.6, 0.9 Hz, 2H), 7.85 (d, *J* = 8.5 Hz, 2H), 7.76 (dd, *J* = 7.4, 1.2 Hz, 2H), 7.55 (d, *J* = 8.1 Hz, 2H), 7.48 – 7.39 (m, 2H), 7.35 (td, *J* = 7.4, 1.2 Hz, 2H), 4.53 (d, *J* = 6.5 Hz, 2H), 4.33 (t, *J* = 6.5 Hz, 1H) ppm. **HRMS-ESI (*m/z*)**: [*M* + Na]$^+$ calcd for $C_{22}H_{17}NO_4$: 382.1050, found: 382.1049. The analytical data are in accordance with the literature.[6]

*4-Diazo-4H-imidazole-5-carboxamide (**11**)*

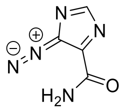

11

A solution of 5-amino-1*H*-imidazole-4-carboxamide hydrochloride (3.50 g,21.5 mmol, 1.00 equiv.) in 1 M HCl (28 mL) was slowly added dropwise to a solution of sodium nitrite (1.63 g, 23.7 mmol, 1.10 equiv.) in water (42 mL) at 0°C in an ice-bath. With every drop, the orange-reddish color of the suspension intensified and more and more precipitate formed. After completion of the addition, the dark red suspension was stirred for further 15 min at 0°C, before the solids were collected by suction filtration and thoroughly washed with water. Drying in a desiccator over $CaCl_2$ under vacuum for several days afforded **11** as a deep-red powder (3.02 g, 22.0 mmol, 79%). ^{1}**H-NMR** (400 MHz, DMSO-d_6): δ= 7.99 (s, 1H), 7.80 (s, 1H), 7.61 (s, 1H) ppm. ^{13}**C-NMR** (101 MHz, DMSO-d_6): δ= 161.3, 155.4, 149.7, 102.3 ppm. **MS-ESI (*m/z*)**: [*M* + Na]$^+$ calcd for $C_4H_3N_5O$: 160.023, found: 160.024; [*M* - H]$^-$ calcd for $C_4H_3N_5O$: 136.026, found: 136.027. Synthesized according to literature.[7]

*3-(2-Chloroethyl)-4-oxo-3,4-dihydroimidazo[5,1-*d*][1,2,3,5]tetrazine-8-carboxamide (**12**)*

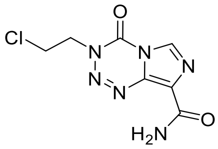

12

To a suspension of 4-diazo-4*H*-imidazole-5-carboxamide (2.00 g, 14.6 mmol, 1.00 equiv.) in dry EtOAc (70 mL) were slowly added via syringe 6.60 mL (8.16 g, 77.3 mmol, 5.30 equiv.) chloroethyl isocyanate. The dark red suspension was stirred for 72 h in the dark at room temperature, before being diluted with diethyl ether (180 mL). More solid material formed, which was collected by suction filtration and dried, yielding **12** as a red powder (2.84 g, 11.7 mmol, 80%). ^{1}**H-NMR** (400 MHz, DMSO-d_6): δ= 8.87 (s, 1H), 7.83 (s, 1H), 7.70 (s, 1H), 4.64 (t, *J* = 6.1 Hz, 2H), 4.03 (t, *J* = 6.1 Hz, 2H) ppm. ^{13}**C-NMR** (101 MHz, DMSO-d_6): δ= 161.4, 139.1, 134.0, 131.2, 129.1, 50.0, 41.5 ppm. **MS-ESI (*m/z*)**: [*M* + Na]$^+$ calcd for $C_7H_7ClN_6O_2$: 265.021, found: 265.021.

*3-(2-Chloroethyl)-4-oxo-3,4-dihydroimidazo[5,1-*d*][1,2,3,5]tetrazine-8-carboxylic acid (**13**)*

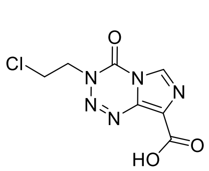

13

A solution of sodium nitrite (2.56 g, 37.1 mmol, 3.60 equiv.) in 7.50 mL water was added very slowly and under vigorous stirring to a suspension of 2.50 g (10.30 mmol, 1.00 equiv.) **12** in concentrated sulfuric acid (15 mL) at 0°C in an ice-bath. Under heavy evolution of gases, the suspension became lighter in color and more precipitate formed, while the temperature was maintained at max. 10°C. After completion of the addition (ca. 1 h), the mixture was warmed to room temperature and vigorously stirred for further 3 h, before being poured into crushed ice (ca. 100 g). An off-white solid formed, which was collected by suction filtration and dried *in vacuo* to yield **13** as a peach-colored powder (1.76 g, 7.23 mmol, 70%). ^{1}**H-NMR** (400 MHz, DMSO-d_6): δ= 8.86 (s, 1H), 4.65 (t, *J* = 6.1 Hz, 2H), 4.02

(t, J = 6.1 Hz, 2H) ppm. **^{13}C-NMR** (101 MHz, DMSO-d_6): δ= 161.7, 138.9, 135.9, 129.7, 128.4, 50.1, 41.4 ppm. **HRMS-ESI (*m/z*)**: [M + Na]$^+$ calcd for $C_7H_6ClN_5O_3$: 266.005, found: 266.005. Synthesized according to literature.[8]

*3-Methyl-4-oxo-3,4-dihydroimidazo[5,1-*d*][1,2,3,5]tetrazine-8-carboxylic acid (**14**)*

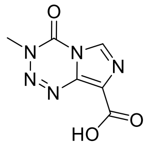

14

A solution of sodium nitrite (2.56 g (37.1 mmol, 3.60 equiv.) in 20 mL water was added very slowly and under vigorous stirring to a suspension of 2.00 g (10.3 mmol, 1.00 equiv.) temozolomide in concentrated sulfuric acid (20 mL) at 0°C in an ice-bath. Under heavy evolution of gases, the suspension became lighter in color and more precipitate formed, while the temperature was maintained at max. 10°C. After completion of the addition within 45 min the mixture was warmed to room temperature and vigorously stirred for further 2 h, before being poured into crushed ice (ca. 100 g). An off-white solid formed, which was collected by suction filtration and dried *in vacuo* to yield **14** as a white powder (1.52 g, 7.77 mmol, 76%). **^{1}H-NMR** (400 MHz, DMSO-d_6): δ= 8.80 (s, 1H), 3.87 (s, 3H) ppm. **^{13}C-NMR** (101 MHz, DMSO-d_6): δ= 161.8, 139.0, 136.4, 129.0, 127.8, 36.3 ppm. **MS-ESI (*m/z*)**: [M + Na]$^+$ calcd for $C_6H_5N_5O_3$: 218.028 found: 218.028; [M - H]$^-$ calcd for $C_6H_5N_5O_3$: 194.032, found: 194.032. Synthesized according to literature.[8]

HAIR synthesis

HAIR A

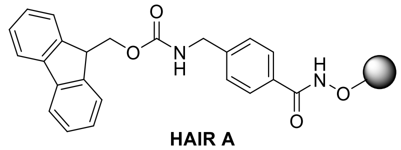

HAIR A

Synthesized according to general procedure C using modified resin **1** (1.00 g, 1.50 mmol, estimated loading 1.50 mmol), 4-(Fmoc-aminomethyl)benzoic acid (1.12 g, 3.00 mmol, 2.00 equiv.), HOBt·H_2O (459 mg, 3.00 mmol, 2.00 equiv.), HATU (1.14 g, 3.00 mmol, 2.00 equiv.) and DIPEA (790 µL, 4.50 mmol, 3.00 equiv.) following general procedure C. Upon completion of the reaction and washing, the resin was dried *in vacuo* and a loading of 0.90 mmol/g was photometrically determined for **HAIR A**.

HAIR B

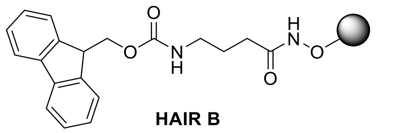

HAIR B

Synthesized according to general procedure C using modified resin **1** (1.00 g, 1.50 mmol, estimated loading 1.50 mmol), 4-Fmoc-aminobutanoic acid (1.12 g, 3.00 mmol, 2.00 equiv.), HOBt·H_2O (459 mg, 3.00 mmol, 2.00 equiv.), HATU (1.14 g, 3.00 mmol, 2.00 equiv) and DIPEA (790 µL, 4.50 mmol, 3.00 equiv) following general procedure C. Upon completion of the reaction and washing, the resin was dried *in vacuo* and a loading of 0.96 mmol/g was photometrically determined for **HAIR B**.

HAIR C

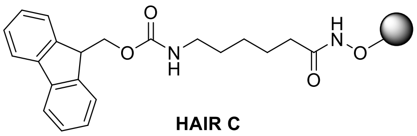

HAIR C

Synthesized according to general procedure C using modified resin **1** (1.19 g, 1.79 mmol, estimated loading 1.50 mmol), 6-Fmoc-aminohexanoic acid (1.26 g, 3.57 mmol, 2.00 equiv.), HOBt·H_2O (547 mg, 3.57 mmol, 2.00 equiv.), HATU (1.36 g, 3.57 mmol, 2.00 equiv.) and DIPEA (933 µL, 5.35 mmol, 3.00 equiv.) following general procedure C. Upon completion of the reaction and washing, the resin was dried *in vacuo* and a loading of 0.87 mmol/g was photometrically determined for **HAIR C**.

HAIR D

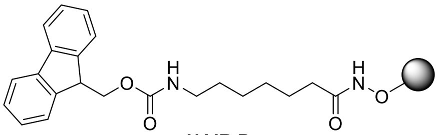

HAIR D

Synthesized according to general procedure C using modified resin **1** (1.22 g, 1.83 mmol, estimated loading 1.50 mmol), 7-Fmoc-aminoheptanoic acid **8b** (1.34 g, 3.65 mmol, 2.00 equiv), HOBt·H_2O (560 mg, 3.65 mmol, 2.00 equiv.), HATU (1.39 g, 3.65 mmol, 2.00 equiv.) and DIPEA (960 µL, 5.50 mmol, 3.00 equiv.) following general procedure C. Upon completion of the reaction and washing, the resin was dried *in vacuo* and a loading of 0.81 mmol/g was photometrically determined for **HAIR D**.

HAIR E

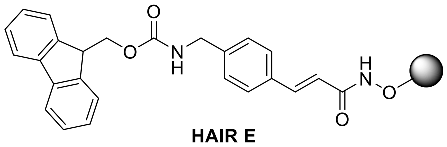

HAIR E

Synthesized according to general procedure C using modified resin **1** (66.6 mg, 0.10 mmol, estimated loading 1.50 mmol), 4-(Fmoc-aminomethyl)phenylacrylic acid **8c** (79.8 mg, 0.20 mmol, 2.00 equiv.), HOBt·H_2O (30.6 mg, 0.20 mmol, 2.00 eq), HATU (76.0 mg, 0.20 mmol, 2.00 equiv.) and DIPEA (51.0 µL, 0.30 mmol, 3.00 equiv.) following general procedure C. Upon completion of the reaction and washing, the resin was dried *in vacuo* and a loading between 0.80 and 0.87 mmol/g was photometrically determined for different batches **HAIR E**.

Library Synthesis

*N-(4-(Hydroxycarbamoyl)benzyl)-3-methyl-4-oxo-3,4-dihydroimidazo[5,1-d][1,2,3, 5] tetrazine-8-carboxamide (**3a**)*

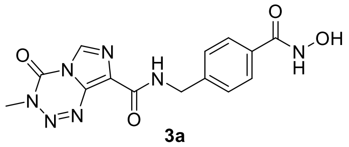

3a

Synthesized according to general procedure D with different equivalents for the reagents using **HAIR A** (200 mg, 0.30 mmol, 1.00 equiv.), HATU (171 mg, 0.45 mmol, 1.50 equiv.), DIPEA (116 mg, 0.90 mmol, 3.00 equiv.) and **14** (88.0 mg, 0.45 mmol, 1.50 equiv.). Purification by preparative HPLC afforded **3a** as beige colored powder (5.0 mg, 14.6 mmol, 5%). **HPLC**: t_R= 12.70 min, **^{1}H-NMR** (400 MHz, DMSO-d_6): δ= 11.16 (s, 1H), 9.13 (t, *J* = 6.3 Hz, 1H), 8.99 (s, 1H), 8.87 (s, 1H), 7.70 (d, *J* = 8.1 Hz, 2H), 7.39 (d, *J* = 8.0 Hz, 2H), 4.53 (d, *J* = 6.3 Hz, 2H), 3.87 (s, 3H) ppm. **^{13}C-NMR** (101 MHz, DMSO-d_6): δ= 164.1, 159.8, 142.8, 139.2, 134.6, 131.3, 130.2, 128.5, 127.1, 126.9, 41.9, 36.1 ppm. **HRMS-ESI (*m/z*)**: $[M + H]^+$ calcd for $C_{14}H_{13}N_7O_4$: 344.1102, found: 344.1101. **IR**: $\tilde{\nu}$= 3281 (br), 1756 (m), 1641 (vs), 1557 (m), 1517 (vs), 1455 (m), 1350 (m), 1251 (s), 1015 (m), 952 (m), 732 (s), 586 (s) cm^{-1}.

*3-(2-Chloroethyl)-N-(4-(hydroxycarbamoyl)benzyl)-4-oxo-3,4-dihydroimidazo[5,1-d][1,2,3,5]tetrazine-8-carboxamide (**3b**)*

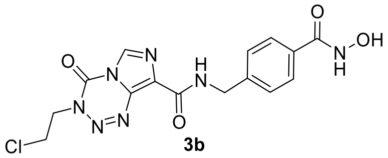

3b

Synthesized according to general procedure D with different equivalents for the reagents using **HAIR A** (400 mg, 0.60 mmol, 1.00 equiv.), HATU (342 mg, 0.90 mmol, 1.50 equiv.), DIPEA (233 mg, 1.80 mmol, 3.00 equiv.) and **13** (219 mg, 0.90 mmol, 1.50 equiv.). Purification by preparative HPLC afforded **3b** as beige coloured powder (56.0 mg, 0.14 mmol, 24%). **HPLC**: t_R= 14.22 min. **^{1}H-NMR** (400 MHz, DMSO-d_6): δ= 11.16 (s, 1H), 9.18 (t, *J* = 6.3 Hz, 1H), 8.96 (s, 1H, broad signal), 8.93 (s, 1H), 7.71 (d, *J* = 8.1 Hz, 2H), 7.40 (d, *J* = 8.0 Hz, 2H), 4.64 (t, *J* = 6.1 Hz, 2H), 4.54 (d, *J* = 6.3 Hz, 2H), 4.03 (t, *J* = 6.1 Hz, 2H) ppm. **^{13}C-NMR** (101 MHz, DMSO-d_6): δ= 164.1, 159.7, 142.7, 139.0, 134.0, 131.3, 130.9, 129.3, 127.1, 126.9, 50.0, 42.0, 41.4 ppm. **HRMS-ESI (*m/z*)**: $[M + H]^+$ calcd for

$C_{15}H_{14}ClN_7O_4$: 392.0869, found 392.0866. **IR**: $\tilde{\nu}$= 3413 (w), 3286 (br), 3128 (w), 2869 (br), 1766 (s), 1644 (vs), 1580 (s), 1519 (s), 1456 (s), 1316 (m), 1255 (s), 1246 (s), 1077 (m), 954 (s), 902 (m), 729 (m), 654 (s), 600 (s), 572 (vs), 554 (s) cm^{-1}.

*4-((4-(4-(Bis(2-chloroethyl)amino)phenyl)butanamido)methyl)-N-hydroxybenz-amide (**3c**)*

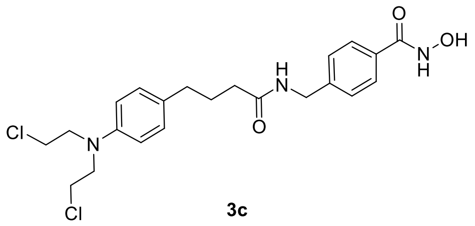

3c

Synthesized according to general procedure D with different equivalents for the reagents using **HAIR A** (200 mg, 0.30 mmol, 1.00 equiv.), HATU (171 mg, 0.45 mmol, 1.50 equiv.), DIPEA (116 mg, 0.90 mmol, 3.00 equiv.) and chlorambucil (137 mg, 0.45 mmol, 1.50 equiv.). Purification by preparative HPLC afforded **3c** as white powder (25.0 mg, 0.06 mmol, 18%). **HPLC**: t_R= 18.17 min. **^{1}H-NMR** (300 MHz, DMSO-d_6): δ= 11.14 (br s, 1H), 8.35 (t, J = 5.9 Hz, 1H), 7.69 (d, J = 8.2 Hz, 2H), 7.29 (d, J = 8.2 Hz, 2H), 7.02 (d, J = 8.6 Hz, 2H), 6.69 – 6.63 (m, 2H), 4.29 (d, J = 5.9 Hz, 2H), 3.69 (s, 8H), 2.45 (d, J = 7.6 Hz, 2H), 2.15 (t, J = 7.5 Hz, 2H), 1.76 (p, J = 7.6 Hz, 2H) ppm. **APT** (75 MHz, DMSO-d_6): δ= 172.0, 164.0, 144.4, 143.0, 131.2, 129.9, 129.3, 127.0, 126.9, 111.9, 52.2, 41.7, 41.2, 34.8, 33.6, 27.4 ppm. **HRMS-ESI (*m/z*)**: [M + H]$^+$ calcd for $C_{22}H_{27}Cl_2N_3O_3$: 452.1502, found 452.1367. **IR**: $\tilde{\nu}$= 3281 (br), 1756 (br), 1640 (s), 1613 (s), 1552 (m), 1432 (br), 1176 (vs), 1153 (vs), 1015 (m), 799 (m), 703 (m) cm^{-1}.

*N-(4-(Hydroxyamino)-4-oxobutyl)-3-methyl-4-oxo-3,4-dihydroimidazo[5,1-d][1,2,3,5]tetrazine-8-carboxamide (**3d**)*

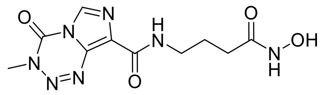

3d

Synthesized according to general procedure D using **HAIR B** (400 mg, 0.36 mmol, 1.00 equiv.), HATU (407 mg, 1.07 mmol, 3.00 equiv.), DIPEA (302 µL, 1.78 mmol, 5.00 equiv.) and **14** (209 mg, 1.07 mmol, 3.00 equiv.). Purification by preparative HPLC afforded **3d** as beige coloured solid (80.7 mg, 0.27 mmol, 76%). **HPLC**: t_R= 11.33 min. **^{1}H-NMR** (400 MHz, DMSO-d_6): δ= 10.39 (s, 1H), 8.84 (s, 1H), 8.55 (t, J = 6.0 Hz, 1H), 3.87 (s, 3H), 3.29 (q, J = 6.7 Hz, 2H), 2.01 (dd, J = 8.4, 6.7 Hz, 2H), 1.82 – 1.70 (m, 2H) ppm, , -C-N*H*-OH signal could not be detected due to solvent exchange. **^{13}C-NMR** (101 MHz, DMSO-d_6): δ= 168.8, 159.7, 139.2, 134.4, 130.5, 128.4, 38.3, 36.1, 30.0, 25.5 ppm. **HRMS-ESI (*m/z*)**: [M + Na]$^+$ calcd for $C_{10}H_{13}N_7O_4$: 318.0921 found:318.0927. **IR**: $\tilde{\nu}$= 3223 (br), 3127 (br), 2935 (br), 1742 (vs), 1641 (s), 1579 (s), 1518 (m), 1455 (vs), 1351 (w), 1248 (m), 1170 (s), 945 (br), 735 (vs), 595 (br) cm^{-1}.

*3-(2-Chloroethyl)-N-(4-(hydroxyamino)-4-oxobutyl)-4-oxo-3,4-dihydroimidazo [5,1-d][1,2,3,5]tetrazine-8-carboxamide (**3e**)*

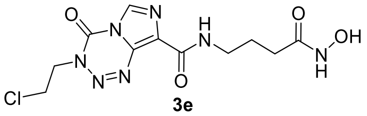

3e

Synthesized according to general procedure D using **HAIR B** (400 mg, 0.36 mmol, 1.00 equiv.), HATU (407 mg, 1.07 mmol, 3.00 equiv.), DIPEA (302 µL, 1.78 mmol, 5.00 equiv.) and **13** (260 mg, 1.07 mmol, 3.00 equiv.). Purification by preparative HPLC afforded **3e** as beige coloured solid (73.4 mg, 0.21 mmol, 59%). **HPLC**: t_R= 13.10 min. **1H-NMR** (400 MHz, DMSO-d6): δ= 10.40 (s, 1H), 8.90 (s, 1H), 8.61 (t, J = 6.1 Hz, 1H),4.64 (t, J = 6.1 Hz, 2H), 4.03 (t, J = 6.1 Hz, 2H), 3.29 (q, J = 6.7 Hz, 2H), 2.01 (t, J = 7.6 Hz, 2H), 1.76 (p, J = 7.2 Hz, 2H) ppm, -C-N*H*-OH signal could not be detected due to solvent exchange. **^{13}C-NMR** (101 MHz, DMSO-d6): δ= 168.8, 159.5, 139.1, 133.8, 131.2, 129.1, 49.9, 41.5, 38.3, 30.0, 25.5 ppm. **HRMS-ESI (*m/z*)**: [M + H]$^+$ calcd for $C_{11}H_{14}ClN_7O_4$: 344.0869, found 344.0864. **IR**: $\tilde{\nu}$= 3224 (br), 3129 (br), 2935 (br), 1743 (vs), 1642 (s), 1579 (m), 1455 (s), 1247 (m), 1157 (s), 1076 (m), 735 (vs) cm^{-1}.

*4-(4-(Bis(2-chloroethyl)amino)phenyl)-N-(4-(hydroxyamino)-4-oxobutyl)butanamide (**3f**)*

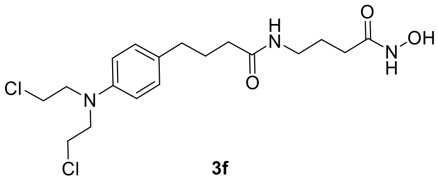

3f

Synthesized according to general procedure D using **HAIR B** (270 mg, 0.24 mmol, 1.00 equiv.), HATU (273 mg, 0.72 mmol, 3.00 equiv.), DIPEA (210 µL, 1.20 mmol, 5.00 equiv.) and chlorambucil (219 mg, 0.72 mmol, 3.00 equiv.). Purification by preparative HPLC afforded **3f** as beige coloured solid (59.5 mg, 0.15 mmol, 63%). **HPLC**: t_R= 17.38 min. **^{1}H-NMR** (300 MHz, DMSO-d_6): δ= 10.35 (s, 1H), 7.77 (t, *J* = 5.6 Hz, 1H), 7.01 (d, *J* = 8.6 Hz, 2H), 6.66 (d, *J* = 8.7 Hz, 2H), 3.69 (s, 8H), 3.01 (q, *J* = 6.9 Hz, 2H), 2.42 (t, *J* = 7.6 Hz, 2H), 2.04 (t, *J* = 7.5 Hz, 2H), 1.94 (dd, *J* = 8.3, 6.8 Hz, 2H), 1.72 (qd, *J* = 8.3, 6.6 Hz, 2H), 1.66 – 1.53 (m, 2H) ppm, -C-N*H*-OH signal could not be detected due to solvent exchange. **^{13}C-NMR** (75 MHz, DMSO-d_6): δ= 171.9, 168.8, 144.4, 130.0, 129.3, 111.9, 52.2, 41.2, 38.1, 34.9, 33.7, 29.9, 27.4, 25.4 ppm. **HRMS-ESI (*m/z*)**: [*M* - H]$^-$ calcd for $C_{18}H_{27}Cl_2N_3O_3$: 402.1357 found: 402.1356. **IR**: $\tilde{\nu}$= 3222 (br), 2935 (br), 1742 (vs), 1639 (vs), 1579 (s), 1518 (s), 1350 (w), 1248 (m), 1173 (s), 947 (m), 735 (vs), 596 (m) cm^{-1}.

*3-(2-Chloroethyl)-N-(6-(hydroxyamino)-6-oxohexyl)-4-oxo-3,4-dihydroimidazo[5,1-d][1,2,3,5]tetrazine-8-carboxamide (**3g**)*

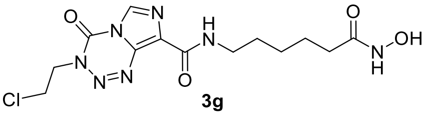

3g

Synthesized according to general procedure D using **HAIR C** (450 mg, 0.38 mmol, 1.00 equiv.), HATU (437 mg, 1.15 mmol, 3.00 equiv.), DIPEA (326 µL, 1.92 mmol, 5.00 equiv.) and **13** (279 mg, 1.15 mmol, 3.00 equiv.). Purification by preparative HPLC afforded **3g** as beige coloured solid (83.4 mg, 0.22 mmol, 58%). **HPLC**: t_R= 13.87 min. **^{1}H-NMR** (400 MHz, DMSO-d_6): δ= 10.32 (s, 1H), 8.89 (s, 1H), 8.51 (t, *J* = 6.0 Hz, 1H), 4.63 (t, *J* = 6.1 Hz, 2H), 4.03 (t, *J* = 6.1 Hz, 2H), 3.28 (q, *J* = 6.8 Hz, 2H), 1.94 (t, *J* = 7.4 Hz, 2H), 1.52 (h, *J* = 7.1 Hz, 4H), 1.27 (qd, *J* = 9.4, 8.9, 6.1 Hz, 2H) ppm, -C-N*H*-OH signal could not be detected due to solvent exchange. **^{13}C-NMR** (101 MHz, DMSO-d_6): δ= 169.0, 159.4, 139.1, 133.8, 131.3, 129.1, 49.9, 41.5, 38.5, 32.2, 28.9, 26.0, 24.9 ppm. **HRMS-ESI (*m/z*)**: [*M* + H]$^+$ calcd for $C_{13}H_{18}ClN_7O_4$: 372.1182, found: 372.1177. **IR**: $\tilde{\nu}$= 3334 (br), 3273 (br), 2941 (br), 2866 (br), 1753 (m), 1682 (m), 1629 (m), 1257 (m), 1452 (m), 1251 (s), 1200 (m), 733 (vs) cm^{-1}.

*6-(4-(4-(Bis(2-chloroethyl)amino)phenyl)butanamido)-N-hydroxyhexanamide (**3h**)*

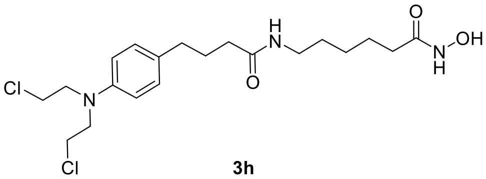

3h

Synthesized according to general procedure D using **HAIR C** (450 mg, 0.38 mmol, 1.00 equiv.), HATU (437 mg, 1.15 mmol, 3.00 equiv.), DIPEA (326 µL, 1.92 mmol, 5.00 equiv.) and chlorambucil (349 mg, 1.15 mmol, 3.00 equiv). Purification by preparative HPLC afforded **3h** as beige coloured solid (84.5 mg, 0.20 mmol, 51%). **HPLC**: t_R= 17.86 min. **^{1}H-NMR** (400 MHz, DMSO-d_6): δ= 10.32 (s, 1H), 7.72 (t, *J* = 5.6 Hz, 1H), 7.01 (d, *J* = 8.7 Hz, 2H), 6.66 (d, *J* = 8.7 Hz, 2H), 3.75 – 3.60 (m, 8H), 3.00 (td, *J* = 7.0, 5.6 Hz, 2H), 2.42 (t, *J* = 7.6 Hz, 2H), 2.04 (t, *J* = 7.5 Hz, 2H), 1.92 (t, *J* = 7.4 Hz, 2H), 1.78 – 1.65 (m, 2H), 1.47 (p, *J* = 7.5 Hz, 2H), 1.36 (p, *J* = 7.3 Hz, 2H), 1.28 – 1.14 (m, 2H) ppm, -C-N*H*-OH signal could not be detected due to solvent exchange. **^{13}C-NMR** (101 MHz, DMSO-d_6): δ= 171.7, 169.0, 144.4, 130.0, 129.3, 111.9, 52.2, 41.2, 38.3, 34.9, 33.7, 32.2, 28.9, 27.4, 26.0, 24.9 ppm. **HRMS-ESI (*m/z*)**: [*M* + H]$^+$ calcd for $C_{20}H_{31}Cl_2N_3O_3$: 432.1815, found 432.1816. **IR**: $\tilde{\nu}$= 3223 (br), 2934 (br), 1743 (vs), 1639 (vs), 1579 (s), 1518 (s), 1455 (vs), 1173 (s), 946 (m), 735 (vs) cm^{-1}.

*N-(7-(Hydroxyamino)-7-oxoheptyl)-3-methyl-4-oxo-3,4-dihydroimidazo[5,1-d][1,2,3,5]tetrazine-8-carboxamide (**3i**)*

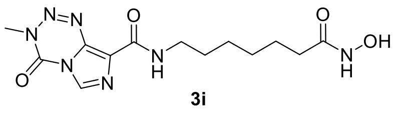

3i

Synthesized according to general procedure D using **HAIR D** (500 mg, 0.41 mmol, 1.00 equiv.), HATU (468 mg, 1.23 mmol, 3.00 equiv.), DIPEA (349 µL, 2.05 mmol, 5.00 equiv.) and **14** (242 mg, 1.23 mmol, 3.00 equiv.). Purification by preparative HPLC afforded **3i** as colorless powder (114 mg, 0.34 mmol, 83%). **HPLC**: t_R= 12.25 min. **^{1}H-NMR** (400 MHz, DMSO-d_6): δ= 10.32 (s, 1H), 8.82 (s, 1H), 8.46 (t, *J* = 6.0 Hz, 1H), 3.86 (s, 3H), 3.28 (q, *J* = 6.7 Hz, 2H), 1.93 (t, *J* = 7.3 Hz, 2H), 1.50 (dp, *J* = 14.5, 6.9 Hz, 4H), 1.27

(dq, *J* = 9.0, 5.2, 4.5 Hz, 4H) ppm, -C-N*H*-OH signal could not be detected due to solvent exchange. **^{13}C-NMR** (101 MHz, DMSO-d_6): δ= 169.1, 159.6, 139.2, 134.4, 130.6, 128.4, 38.5, 36.1, 32.2, 29.1, 28.4, 26.1, 25.1 ppm. **HRMS-ESI (*m/z*)**: [*M* + Na]$^+$ calcd for $C_{13}H_{19}N_7O_4$: 360.1391, found: 360.1401. **IR**: $\tilde{\nu}$= 3301 (br), 3195 (br), 2911 (br), 2847 (br), 1736 (vs), 1622 (m), 1580 (s), 1255 (m), 1044 (m), 948 (s) cm^{-1}.

*3-(2-Chloroethyl)-N-(7-(hydroxyamino)-7-oxoheptyl)-4-oxo-3,4-dihydroimidazo [5,1-d][1,2,3,5]tetrazine-8-carboxamide (**3j**)*

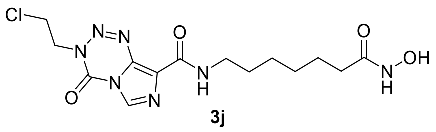

3j

Synthesized according to general procedure D using **HAIR D** (500 mg, 0.41 mmol, 1.00 equiv.), HATU (468 mg, 1.23 mmol, 3.00 equiv.), DIPEA (349 µL, 2.05 mmol, 5.00 equiv.) and **13** (300 mg, 1.23 mmol, 3.00 equiv.). Purification by preparative HPLC afforded **3j** as colorless powder (108 mg, 0.28 mmol, 68%). **HPLC**: t_R= 13.63 min. **^{1}H-NMR** (400 MHz, DMSO-d_6): δ= 10.32 (s, 1H), 8.88 (s, 1H), 8.50 (t, *J* = 6.0 Hz, 1H), 4.64 (t, *J* = 6.1 Hz, 2H), 4.03 (t, *J* = 6.1 Hz, 2H), 3.28 (q, *J* = 6.7 Hz, 2H), 1.93 (t, *J* = 7.4 Hz, 2H), 1.50 (m, *J* = 17.5, 7.1 Hz, 4H), 1.27 (m, *J* = 10.9, 9.3, 4.4 Hz, 4H) ppm, -C-N*H*-OH signal could not be detected due to solvent exchange. **^{13}C-NMR** (101 MHz, DMSO-d_6): δ= 169.1, 159.4, 139.1, 133.8, 131.3, 129.1, 49.9, 41.5, 38.5, 32.2, 29.1, 28.3, 26.1, 25.1 ppm. **HRMS-ESI (*m/z*)**: [*M* + Na]$^+$ calcd for $C_{14}H_{20}ClN_7O_4$: 408.1158, found: 408.1158. **IR:** $\tilde{\nu}$= 3251 (br), 2927 (br), 2854 (br), 1736 (vs), 1630 (vs), 1580 (s), 1519 (m), 1456 (s), 1318 (m), 1250 (m), 1088 (m), 926 (m), 737 (m), 643 (m) cm^{-1}.

*7-(4-(4-(Bis(2-chloroethyl)amino)phenyl)butanamido)-N-hydroxyheptanamide (**3k**)*

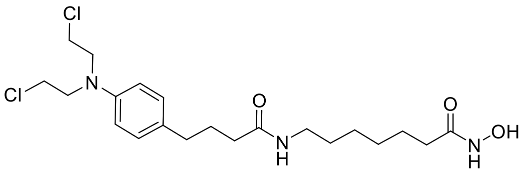

3k

Synthesized according to general procedure D using **HAIR D** (500 mg, 0.41 mmol, 1.00 equiv.), HATU (468 mg, 1.23 mmol, 3.00 equiv.), DIPEA (349 µL, 2.05 mmol, 5.00 equiv.) and chlorambucil (374 mg, 1.23 mmol, 3.00 equiv.). Purification by preparative HPLC afforded **3k** as colorless powder (93.0 mg, 0.21 mmol, 51%). **HPLC**: t_R= 17.53 min. **^{1}H-NMR** (300 MHz, DMSO-d_6): δ= 10.31 (s, 1H), 7.71 (t, *J* = 5.6 Hz, 1H), 7.01 (d, *J* = 8.6 Hz, 2H), 6.66 (d, *J* = 8.7 Hz, 2H), 3.69 (s, 8H), 3.10 – 2.90 (m, 2H), 2.42 (t, *J* = 7.5 Hz, 2H), 2.04 (t, *J* = 7.4 Hz, 2H), 1.92 (t, *J* = 7.3 Hz, 2H), 1.80 – 1.65 (m, 2H), 1.48 (q, *J* = 7.2 Hz, 2H), 1.35 (q, *J* = 6.8 Hz, 2H), 1.22 (p, *J* = 3.5 Hz, 4H) ppm, -C-N*H*-OH signal could not be detected due to solvent exchange. **^{13}C-NMR** (101 MHz, DMSO-d_6): δ= 171.7, 169.1, 144.4, 130.0, 129.3, 111.9, 52.2, 41.2, 38.3, 34.9, 33.6, 32.2, 29.1, 28.3, 27.4, 26.2, 25.1 ppm. **HRMS-ESI (*m/z*)**: [*M* + H]$^+$ calcd for $C_{21}H_{33}Cl_2N_3O_3$: 446.1972, found: 446.1971. **IR**: $\tilde{\nu}$= 3262 (br), 2928 (br), 2852 (br), 1614 (s), 1564 (s), 1517 (vs), 1354 (m), 1178 (m), 804 (m), 740 (s) cm^{-1}.

*(E)-N-(4-(3-(Hydroxyamino)-3-oxoprop-1-en-1-yl)benzyl)-3-methyl-4-oxo-3,4-dihydro-imidazo[5,1-d][1,2,3,5]tetrazine-8-carboxamide (**3l**)*

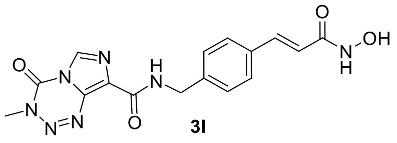

3l

Synthesized according to general procedure D using **HAIR E** (500 mg, 0.41 mmol, 1.00 equiv.), HATU (468 mg, 1.23 mmol, 3.00 equiv.), DIPEA (349 µL, 2.05 mmol, 5.00 equiv.) and **14** (242 mg, 1.23 mmol, 3.00 equiv.). Purification by preparative HPLC afforded **3l** as white coloured powder (63.0 mg, 0.17 mmol, 41%). **HPLC**: t_R= 13.05 min. **^{1}H-NMR** (300 MHz, DMSO-d_6): δ= 10.72 (s, 1H), 9.09 (t, *J* = 6.3 Hz, 1H), 8.86 (s, 1H), 7.52 (d, *J* = 8.1 Hz, 2H), 7.42 (d, *J* = 15.9 Hz, 1H), 7.37 (d, *J* = 8.2 Hz, 2H), 6.43 (d, *J* = 15.8 Hz, 1H), 4.51 (d, *J* = 6.3 Hz, 2H), 3.87 (s, 3H) ppm, -C-N*H*-OH signal could not be detected due to solvent exchange. **^{13}C-NMR** (75 MHz, DMSO-d_6): δ= 162.8, 159.8, 141.1, 139.2, 138.1, 134.6, 133.4, 130.3, 128.5, 127.9, 127.5, 118.6, 41.9, 36.2 ppm. **HRMS-ESI (*m/z*)**: [*M* + H]$^+$ calcd for $C_{16}H_{15}N_7O_4$: 392.1078, found: 392.1060. **IR**: $\tilde{\nu}$= 3209 (br), 3028 (br), 2854 (br), 1745 (vs), 1651 (s), 1582 (m), 1454 (m), 1250 (m), 1054 (m), 943 (m), 734 (s), 568 (m), 509 (s) cm^{-1}.

*(E)-3-(2-Chloroethyl)-N-(4-(3-(hydroxyamino)-3-oxoprop-1-en-1-yl)benzyl)-4-oxo-3,4-dihydroimidazo[5,1-d][1,2,3,5]tetrazine-8-carboxamide (**3m**)*

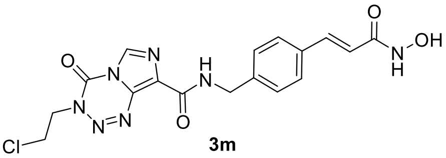

3m

Synthesized according to general procedure D using **HAIR E** (600 mg, 0.41 mmol, 1.00 equiv.), HATU (470 mg, 1.24 mmol, 3.00 equiv.), DIPEA (350 µL, 2.06 mmol, 5.00 equiv.) and **13** (301 mg, 1.24 mmol, 3.00 equiv.). Purification by preparative HPLC afforded **3m** as white coloured powder (94.1 mg, 0.23 mmol, 56%). **HPLC**: t_R= 14.38 min. **^{1}H-NMR** (400 MHz, DMSO-d_6): δ= 10.73 (s, 1H), 9.14 (t, *J* = 6.3 Hz, 1H), 8.92 (s, 1H), 7.52 (d, *J* = 8.0 Hz, 2H), 7.43 (d, *J* = 15.8 Hz, 1H), 7.37 (d, *J* = 8.3 Hz, 2H), 6.43 (d, *J* = 15.8 Hz, 1H), 4.64 (t, *J* = 6.1 Hz, 2H), 4.52 (d, *J* = 6.3 Hz, 2H), 4.03 (t, *J* = 6.1 Hz, 2H) ppm, -C-N*H*-OH signal could not be detected due to solvent exchange. **^{13}C-NMR** (101 MHz, DMSO-d_6): δ= 162.7, 159.7, 141.0, 139.1, 138.1, 134.0, 133.4, 130.9, 129.3, 127.8, 127.5, 118.6, 50.0, 41.9, 41.5 ppm. **HRMS-ESI (*m/z*)**: [*M* + H]$^+$ calcd for $C_{17}H_{16}ClN_7O_4$: 418.1025, found: 418.1025. **IR**: $\tilde{\nu}$= 3387 (br), 3226 (br), 3032 (br), 1738 (vs), 1657 (vs), 1622 (m), 1582 (m), 1519 (m), 1251 (m), 1056 (m), 780 (m), 733 (s), 612 (m), 496 (m) cm^{-1}.

*(E)-4-(4-(Bis(2-chloroethyl)amino)phenyl)-N-(4-(3-(hydroxyamino)-3-oxoprop-1-en-1-yl)benzyl)butanamide (**3n**)*

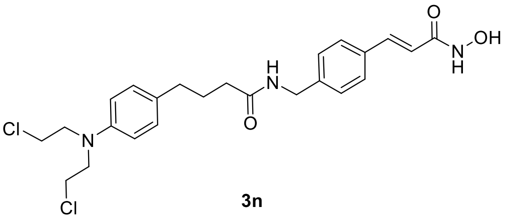

3n

Synthesized according to general procedure D using **HAIR E** (500 mg, 0.40 mmol, 1.00 equiv.), HATU (456 mg, 1.20 mmol, 3.00 equiv.), DIPEA (340 µL, 2.00 mmol, 5.00 equiv.) and chlorambucil (365 mg, 1.20 mmol, 3.00 equiv.). Purification by preparative HPLC afforded **3n** as white coloured powder (48.5 mg, 0.10 mmol, 25%). **HPLC**: t_R= 18.00 min. **^{1}H-NMR** (400 MHz, DMSO-d_6): δ= 10.73 (s, 1H), 8.32 (t, *J* = 5.9 Hz, 1H), 7.50 (d, *J* = 7.9 Hz, 2H), 7.42 (d, *J* = 15.8 Hz, 1H), 7.27 (d, *J* = 8.0 Hz, 2H), 7.02 (d, *J* = 8.6 Hz, 2H), 6.66 (d, *J* = 8.7 Hz, 2H), 6.43 (d, *J* = 15.8 Hz, 1H), 4.27 (d, *J* = 5.9 Hz, 2H), 3.69 (d, *J* = 3.2 Hz, 8H), 2.45 (t, *J* = 7.6 Hz, 2H), 2.14 (t, *J* = 7.5 Hz, 2H), 1.87 – 1.68 (m, 2H) ppm, -C-N*H*-OH signal could not be detected due to solvent exchange.**^{13}C-NMR** (101 MHz, DMSO-d_6): δ= 172.0, 162.7, 144.4, 141.3, 138.0, 133.3, 129.9, 129.3, 127.7, 127.4, 118.6, 111.9, 52.2, 41.8, 41.1, 34.9, 33.6, 27.4 ppm. **HRMS-ESI (*m/z*)**: [*M* + H]$^+$ calcd for $C_{24}H_{29}Cl_2N_3O_3$: 478.1659, found: 478.1654. **IR**: $\tilde{\nu}$= 3227 (br), 3030 (br), 2856 (br), 1745 (w), 1641 (m), 1614 (m), 1517 (s), 1349 (m), 802 (m), 736 (m) cm^{-1}.

*(E)-N-(4-(3-(Hydroxyamino)-3-oxoprop-1-en-1-yl)benzyl)-4-phenylbutanamide (**3o**)*

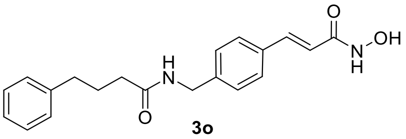

3o

Synthesized according to general procedure D with different equivalents for the reagents using **HAIR E** (170 mg, 0.14 mmol, 1.00 equiv.), HATU (186 mg, 0.49 mmol, 3.50 equiv.), DIPEA (122 µL, 0.70 mmol, 5.00 equiv.) and 4-phenylbutanoic acid (80.0 mg, 0.49 mmol, 3.50 equiv.). Purification by preparative HPLC afforded **3o** as white coloured powder (17.6 mg, 0.05 mmol, 37%). **HPLC**: t_R= 15.72 min, **^{1}H-NMR** (400 MHz, DMSO-d_6): δ= 10.72 (s, 1H), 9.00 (s, 1H, broad signal), 8.34 (t, *J* = 5.9 Hz, 1H), 7.50 (d, *J* = 8.0 Hz, 2H), 7.42 (d, *J* = 15.8 Hz, 1H), 7.32 – 7.23 (m, 4H), 7.22 – 7.13 (m, 3H), 6.42 (d, *J* = 15.8 Hz, 1H), 4.27 (d, *J* = 6.0 Hz, 1H), 2.59 – 2.53 (m, 2H), 2.16 (t, *J* = 7.5 Hz, 2H), 1.89 – 1.73 (m, 2H) ppm. **^{13}C-NMR** (101 MHz, DMSO-d_6): δ= 171.9, 162.8, 141.7, 141.3, 138.1, 133.3, 128.3, 128.3, 127.7, 127.4, 125.7, 118.6, 41.8, 34.8, 34.7, 27.1 ppm. **HRMS-ESI (*m/z*)**: [*M* + H]$^+$ calcd for $C_{20}H_{22}N_2O_3$: 339.1703, found: 339.1705. **IR**: $\tilde{\nu}$= 3288 (br), 2924 (br), 1641 (vs), 1556 (s), 1418 (w), 1350 (w), 1069 (m), 973 (m), 741 (s), 697 (vs), 590 (br), 521 (m), 488 (m) cm^{-1}.

*4-(2-(2-(2-(2-((2-(2,6-Dioxopiperidin-3-yl)-1,3-dioxoisoindolin-4-yl)oxy)acetamido)ethoxy)ethoxy)acetamido)-N-(7-(hydroxyamino)-7-oxoheptyl)benzamide (**4**)*

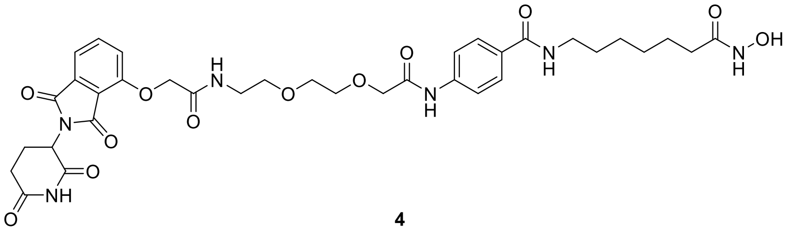

4

After swelling **HAIR E** (156 mg, 0.15 mmol, 1.00 eq) in DMF for 30 min, Fmoc deprotection was performed by treatment with deprotection solution (20% piperidine in DMF, 2 x 15 min). Afterwards the resin was washed with DMF (3 x 5 mL), CH_2Cl_2 (3 x 5 mL) and DMF (3 x 5 mL). For the subsequent amide coupling reaction a solution of 4-(Fmoc-amino)benzoic acid **8d** (162 mg, 0.45 mmol, 3.00 equiv.), HATU (171 mg, 0.45 mmol, 3.00 equiv.), and DIPEA (105 µL, 0.60 mmol, 4.00 equiv.) in DMF (0.5 mL) was agitated for 5 min and then added to the resin. The amide coupling was performed for 16 h at room temperature. Afterwards the resin was washed with DMF (3 x 5 mL), CH_2Cl_2 (3 x 5 mL) and DMF (3 x 5 mL). Completion of the reaction was monitored via TNBS-test. Coupling of the PEG-linker was performed starting with Fmoc deprotection, subsequent washing and coupling using Fmoc-O2Oc-OH (173 mg, 0.45 mmol, 3.00 equiv., supplied by Iris Biotech), HATU (171 mg, 0.45 mmol, 3.00 equiv.), and DIPEA (105 µL, 0.60 mmol, 4.00 equiv.) in DMF (0.5 mL) for 4 h at room temperature. After washing with DMF (3 x 5 mL), CH_2Cl_2 (3 x 5 mL) and DMF (3 x 5 mL) the coupling cycle was repeated using 2-((2-(2,6-dioxopiperidin-3-yl)-1,3-dioxoisoindolin-4-yl)oxy)acetic acid **7** (99.7 mg, 0.30 mmol, 2.00 equiv), HATU (125 mg, 0.33 mmol, 2.20 equiv.), DIPEA (92 µL, 0.53 mmol, 3.50 equiv.) in DMF (500 µL). Amide coupling was performed for 4 h at room temperature. Afterwards the resin was washed with DMF (3 x 5 mL) and CH_2Cl_2 (3 x 5 mL). The resin was dried *in vacuo* followed by the cleavage of the crude products from the resin by treatment with cleavage solution (5% TFA in CH_2Cl_2, 1 mL/40 mg resin) for 1 h at room temperature. The filtrates were concentrated *in vacuo* and the crude product **4** was purified by preparative HPLC. Lyophilization of the respective fractions yielded the desired product **4** (63.5 mg, 86.0 µmol, 57%) in >95% purity. **HPLC**: t_R= 13.65 min. **^{1}H-NMR** (400 MHz, DMSO-d_6): δ= 11.11 (s, 1H), 10.32 (s, 1H), 9.79 (s, 1H), 8.29 (t, *J* = 5.6 Hz, 1H), 8.00 (t, *J* = 5.7 Hz, 1H), 7.89 – 7.73 (m, 3H), 7.68 (d, *J* = 8.8 Hz, 2H), 7.48 (d, *J* = 7.2 Hz, 1H), 7.38 (d, *J* = 8.6 Hz, 1H), 5.10 (dd, *J* = 12.9, 5.4 Hz, 1H), 4.77 (s, 2H), 4.10 (s, 2H)*, 3.73 – 3.65 (m, 2H), 3.66 – 3.59 (m, 2H), 3.52 (t, *J* = 5.6 Hz, 2H), 3.35 (q, *J* = 5.6 Hz, 2H), 3.21 (q, *J* = 6.7 Hz, 2H), 2.95 – 2.83 (m, 1H), 2.69 – 2.52 (m, 2H), 2.10 – 1.98 (m, 1H), 1.94 (t, *J* = 7.3 Hz, 2H), 1.49 (p, *J* = 6.6, 6.1 Hz, 4H), 1.27 (tq, *J* = 8.2, 4.7, 3.3 Hz, 4H) ppm, -N*H* signal could not be detected due to solvent exchange. **^{13}C-NMR** (101 MHz, DMSO-d_6): δ= 172.7, 169.9, 169.1, 168.6, 166.9, 166.7, 165.5, 165.5, 154.9, 140.7, 136.9, 133.0, 129.5, 127.9, 120.3, 118.7, 116.8, 116.0, 70.3, 70.2, 69.4, 68.9, 67.5, 48.8, 39.1*, 38.3, 32.2, 31.0, 29.1, 28.4, 26.2, 25.1, 22.0 ppm, *overlapping with DMSO signal, confirmed by HSQC. **HRMS-ESI (*m/z*)**: $[M + H]^+$ calcd for $C_{35}H_{42}N_6O_{12}$: 739.2933, found: 739.2936. **IR**: $\tilde{\nu}$= 3333 (br), 2930 (br), 1707 (s), 1527 (m), 1394 (m), 1262 (m), 1196 (m), 1116 (m), 747 (m), 602 (m), 466 (m) cm^{-1}.

*N-(6-(Hydroxyamino)-6-oxohexyl)-3-methyl-4-oxo-3,4-dihydroimidazo[5,1-d][1,2,3,5]tetrazine-8-carboxamide (**3p**)*

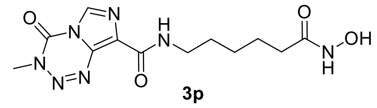

3p

Synthesized according to general procedure D using **HAIR C** (450 mg, 0.38 mmol, 1.00 equiv.), HATU (437 mg, 1.15 mmol, 3.00 equiv.), DIPEA (326 µL, 1.92 mmol, 5.00 equiv.) and **14** (226 mg, 1.15 mmol, 3.00 equiv). This compound turned out to be unstable, already during lyophillization and thus could not be characterized and was excluded from the biological evaluation.

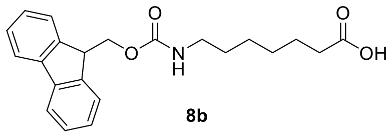

8b

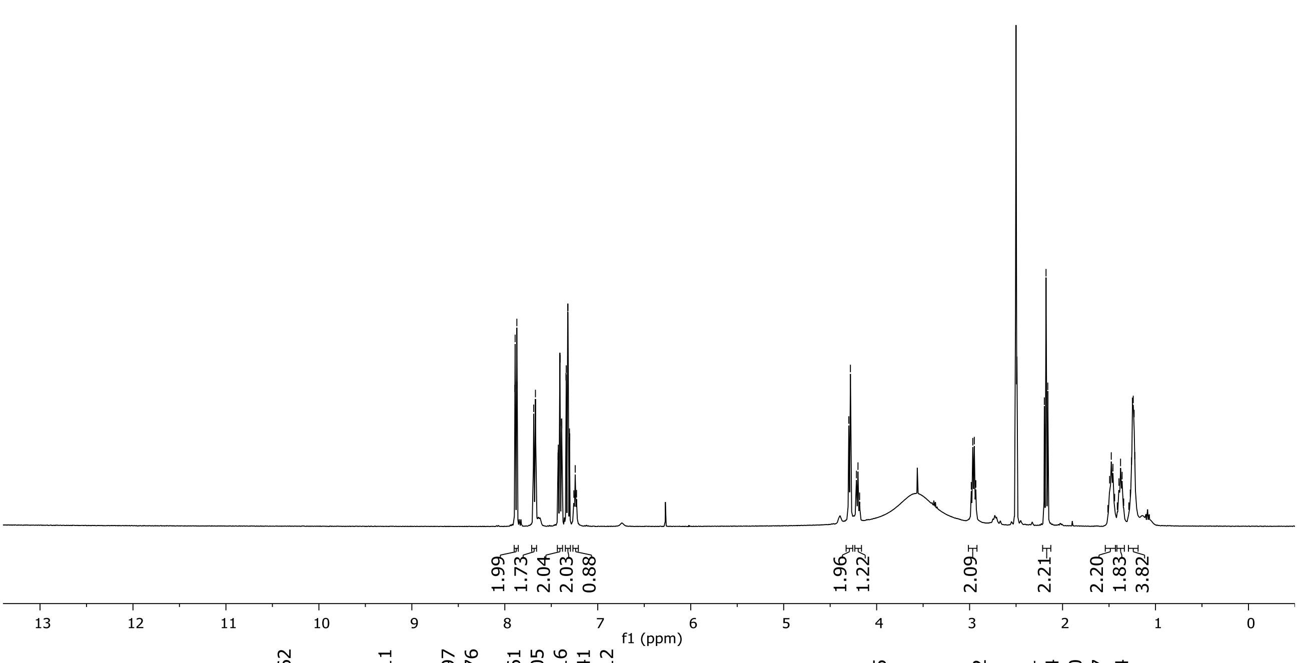

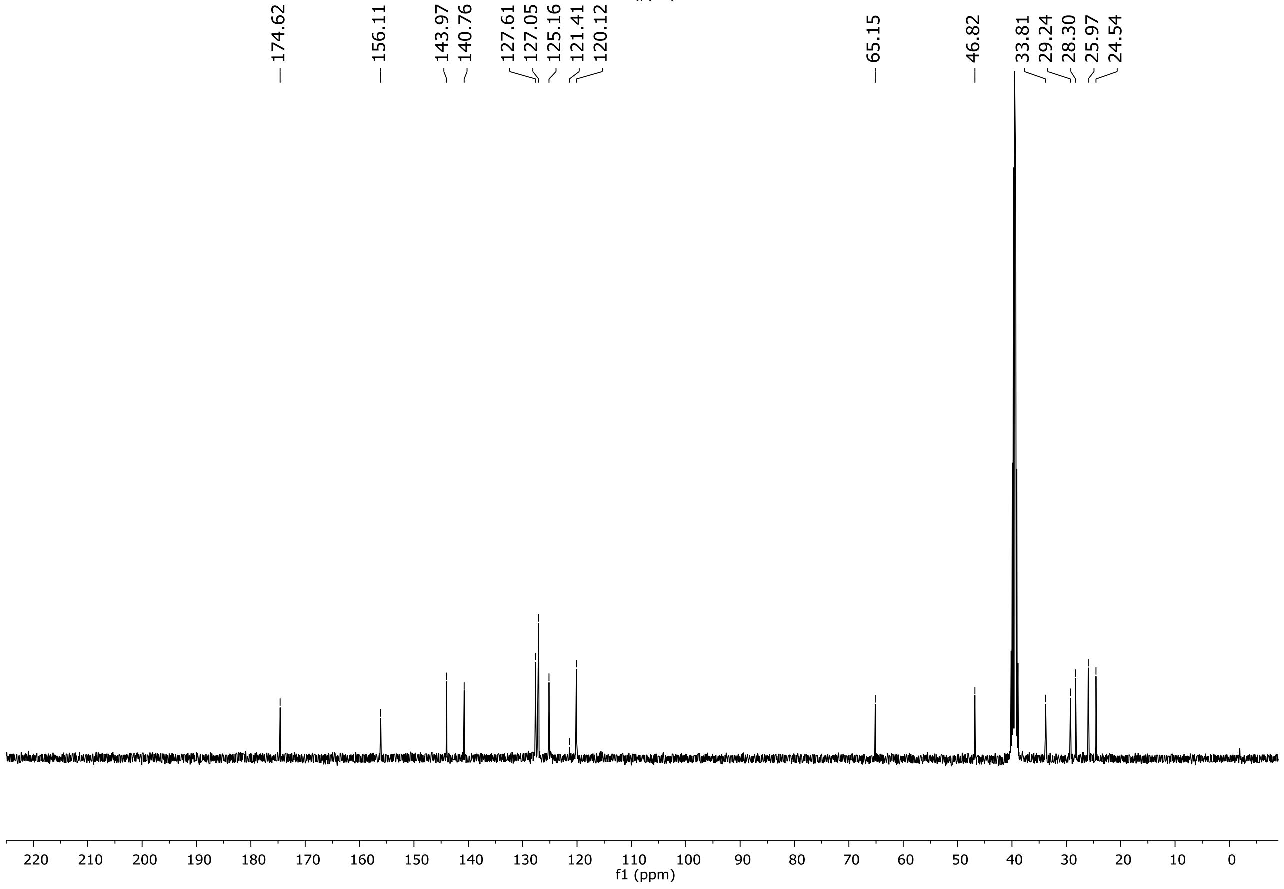

Figure S2: ^{1}H and ^{13}C-NMR spectra of **8b** in DMSO-d_6.

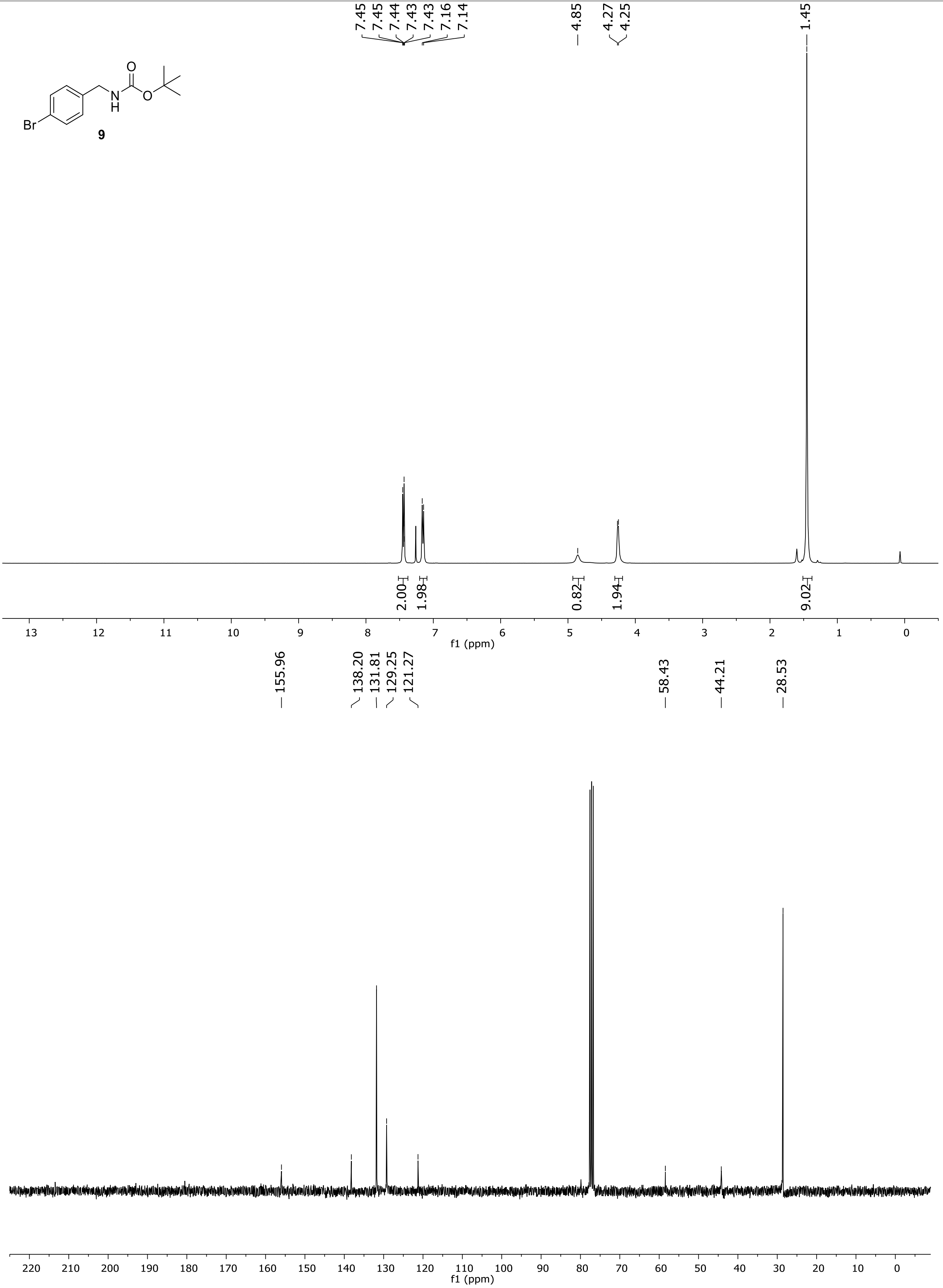

Figure S3: ^{1}H and ^{13}C-NMR spectra of **9** in DMSO-d_6.

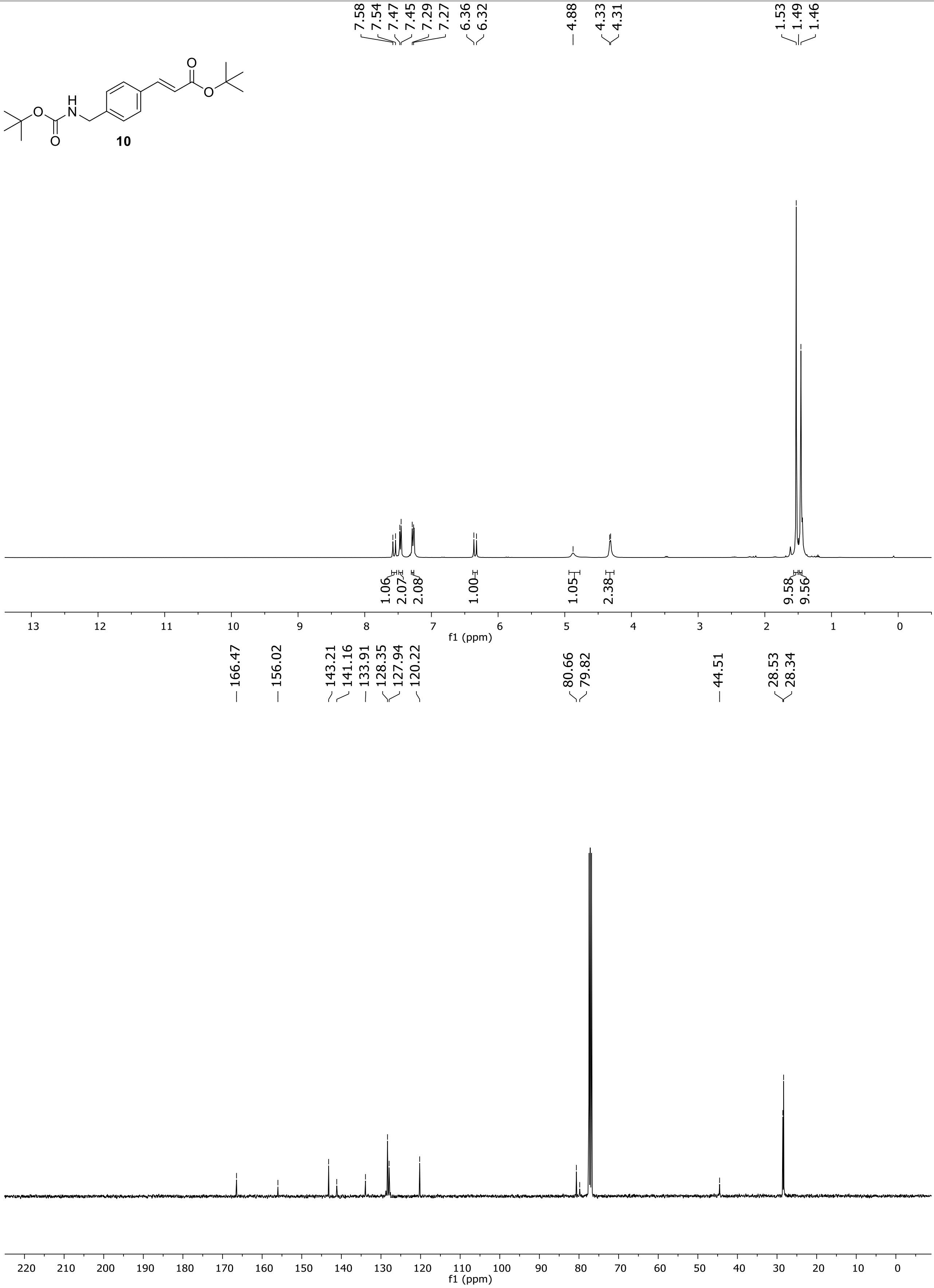

Figure S4: ^{1}H and ^{13}C-NMR spectra of **10** in $CDCl_3$.

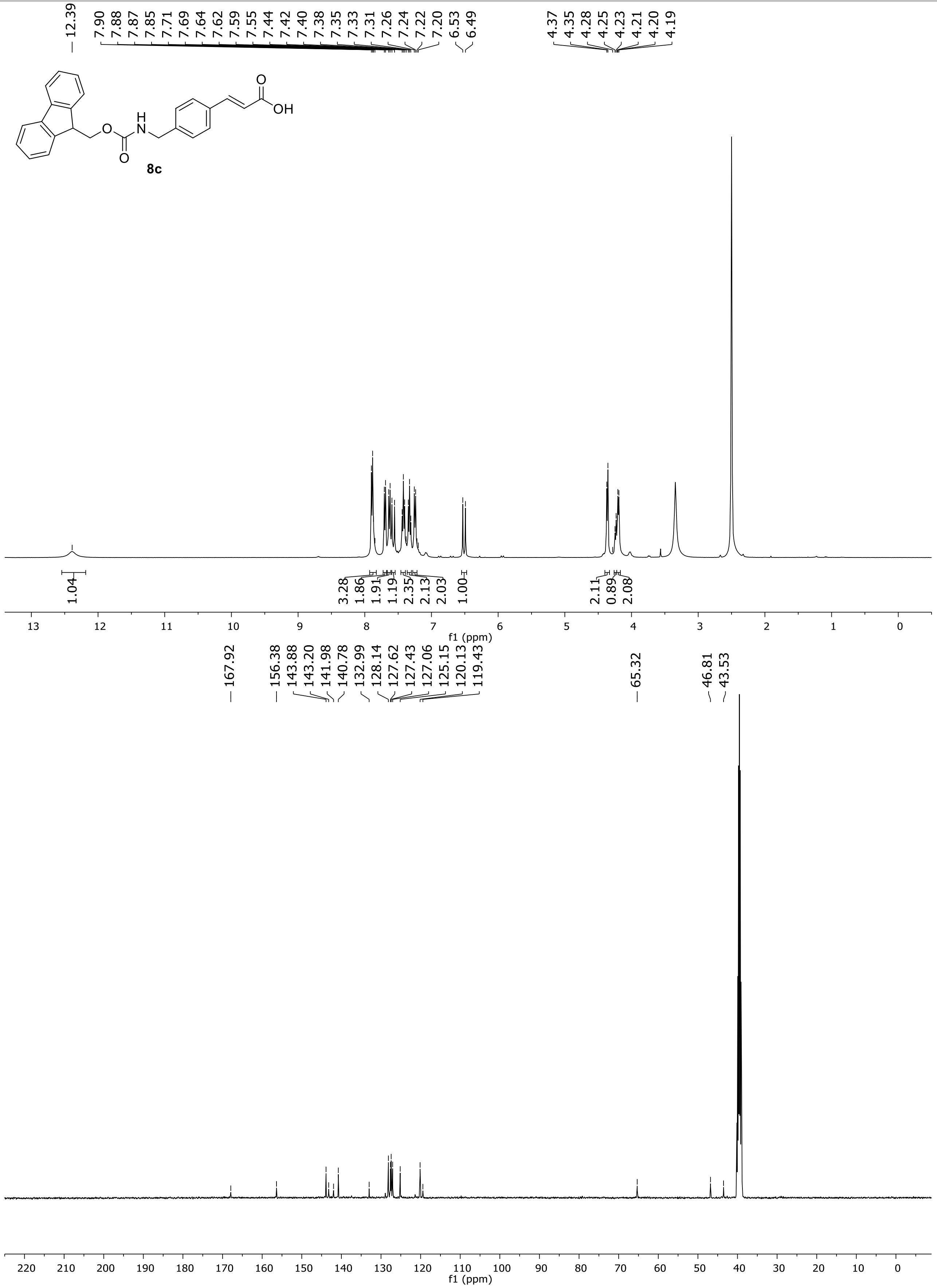

Figure S5: ^{1}H and ^{13}C-NMR spectra of **8c** in DMSO-d_6.

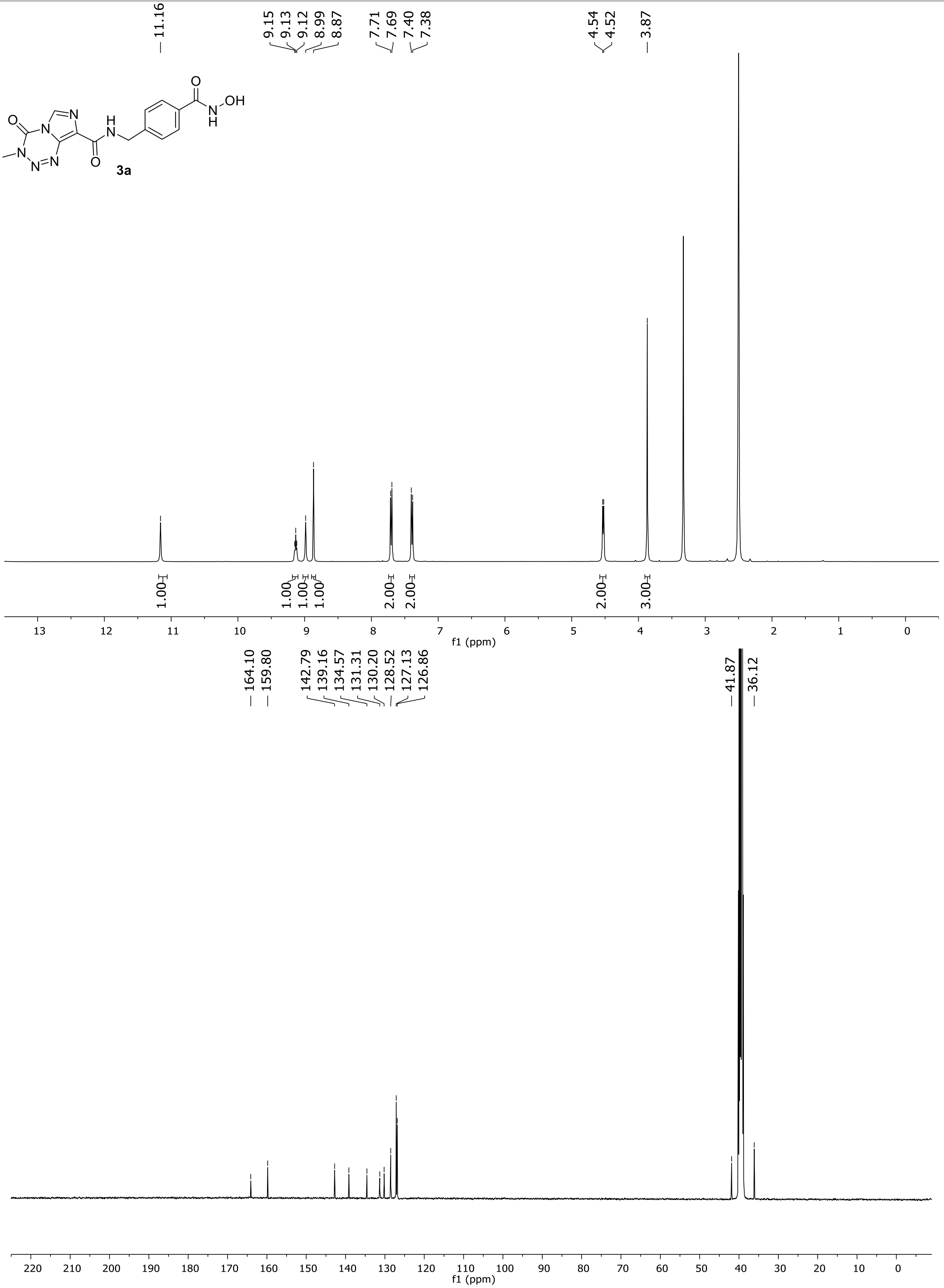

Figure S6: ^{1}H and ^{13}C-NMR spectra of **3a** in DMSO-d_6.

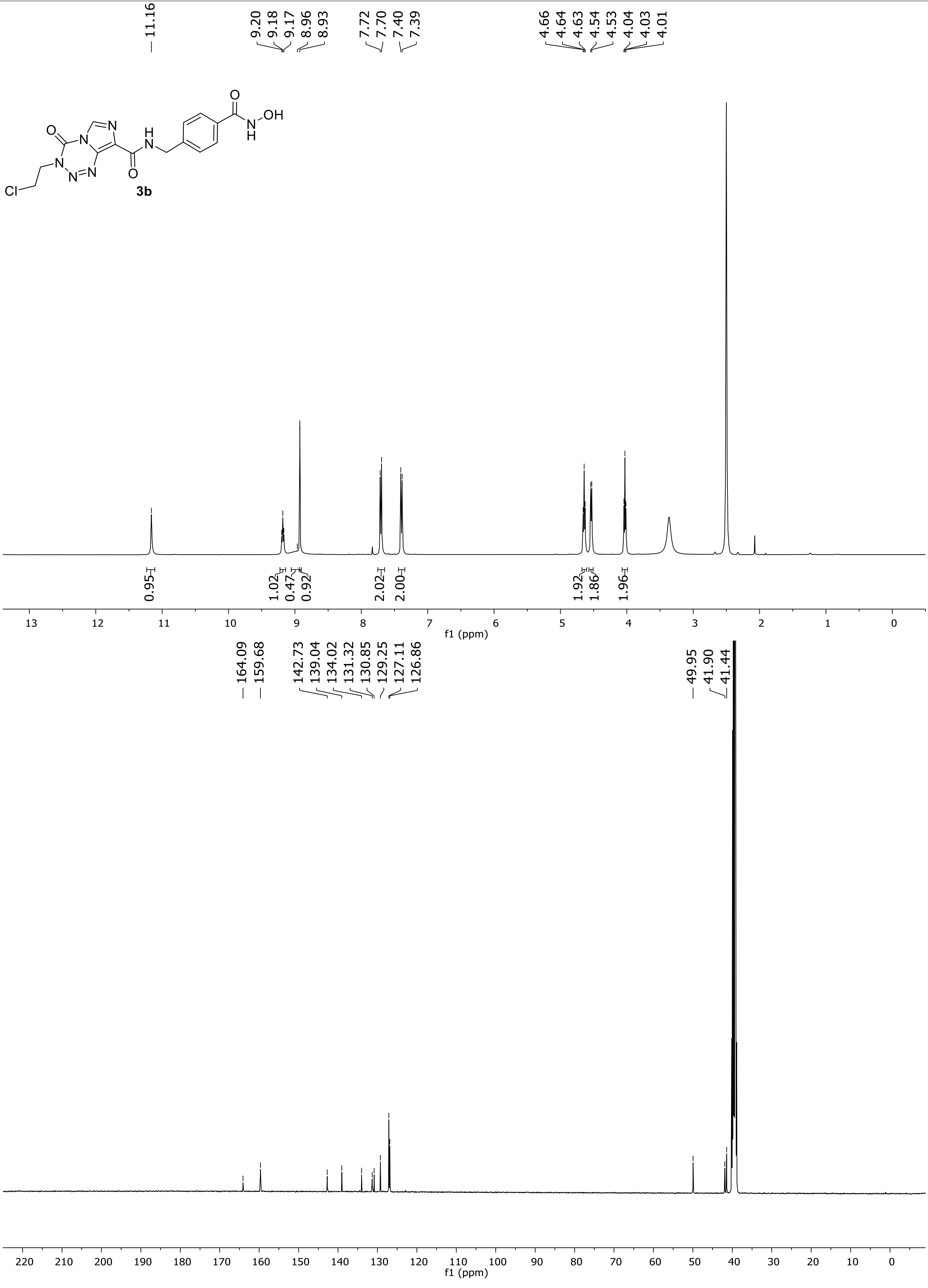

Figure S7: ^{1}H and ^{13}C-NMR spectra of **3b** in DMSO-*d*$_6$.

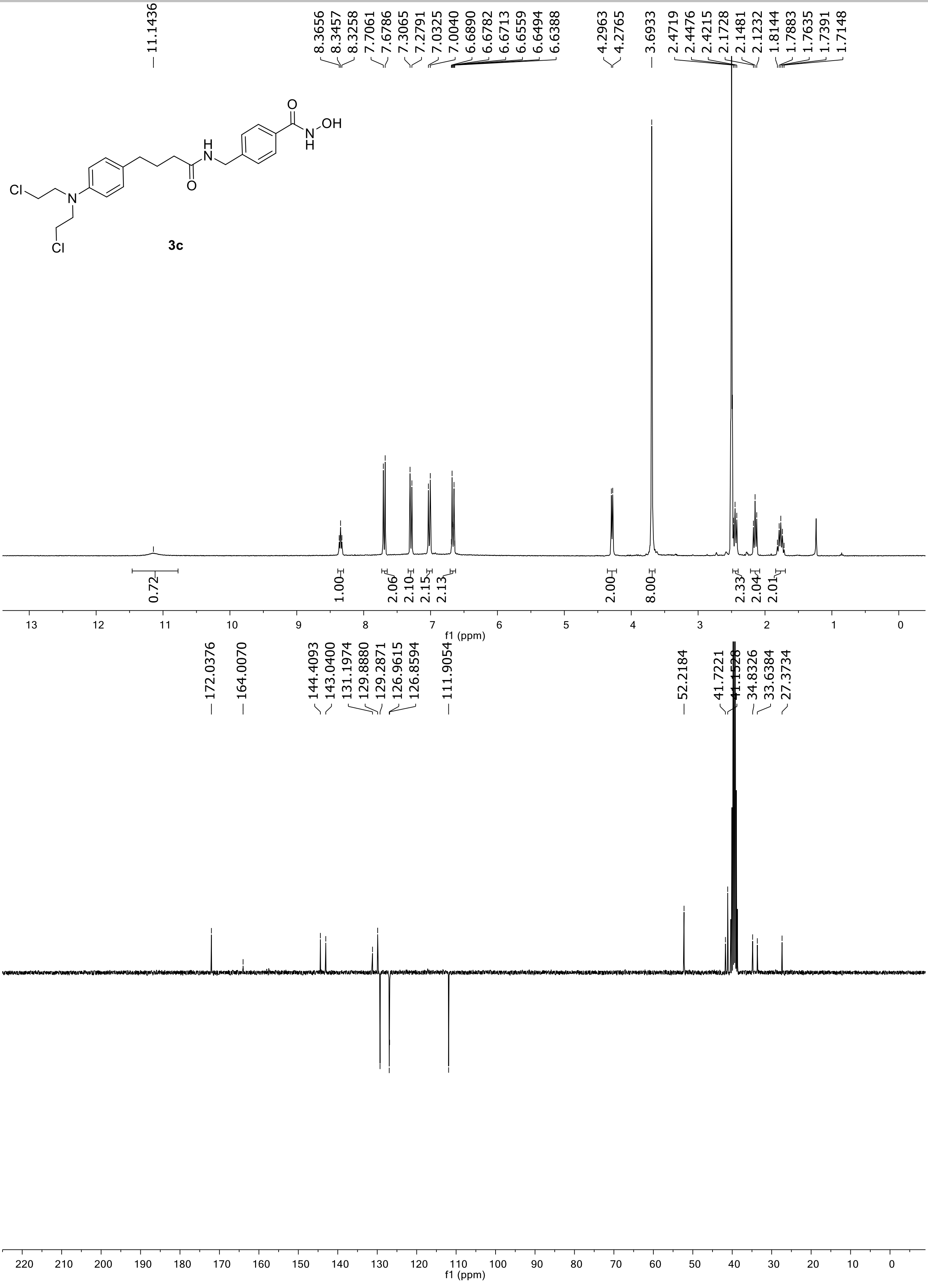

Figure S8: ^{1}H and APT spectra of **3c** in DMSO-d_6.

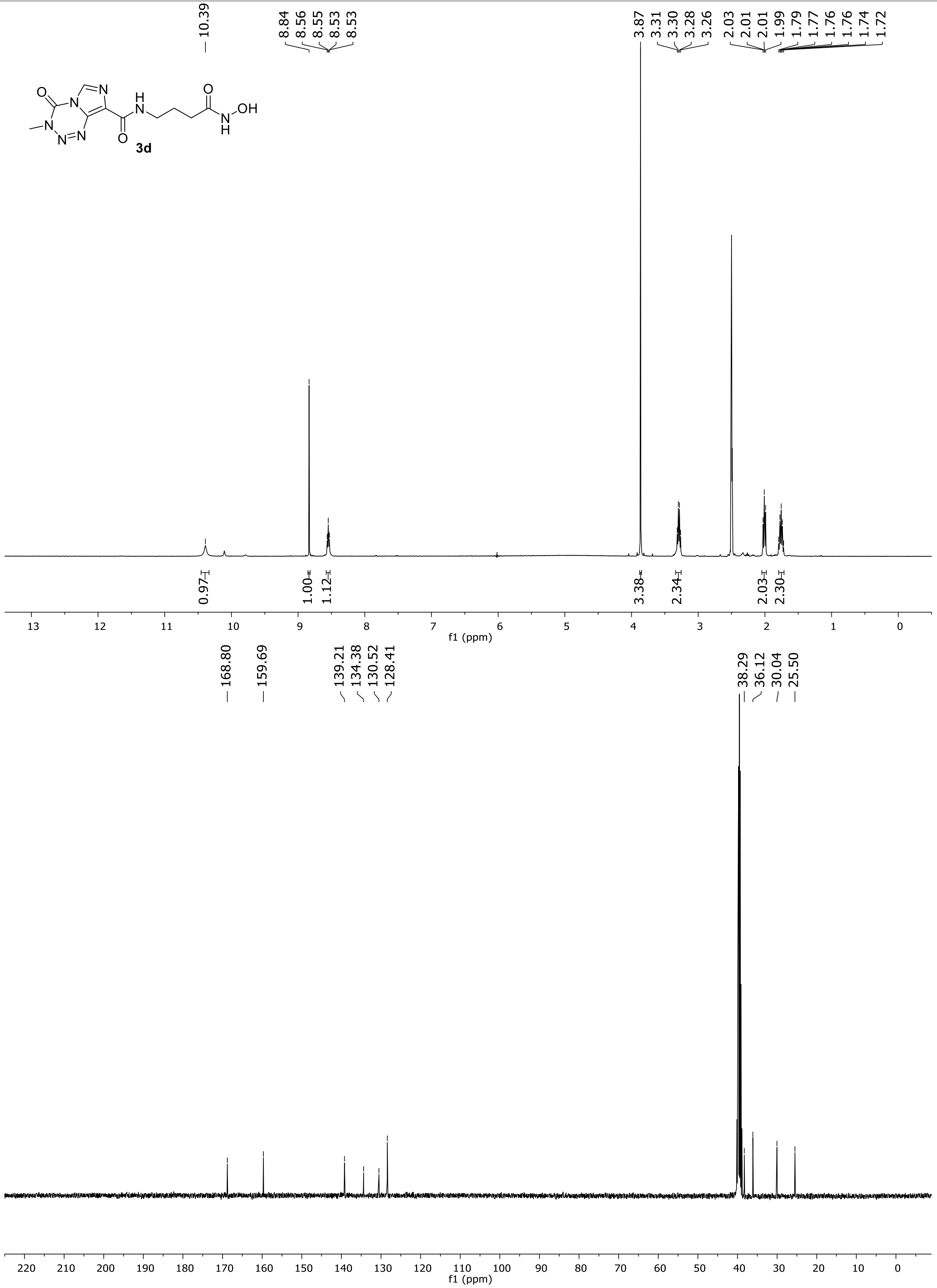

Figure S9: ^{1}H and ^{13}C-NMR spectra of **3d** in DMSO-d_6.

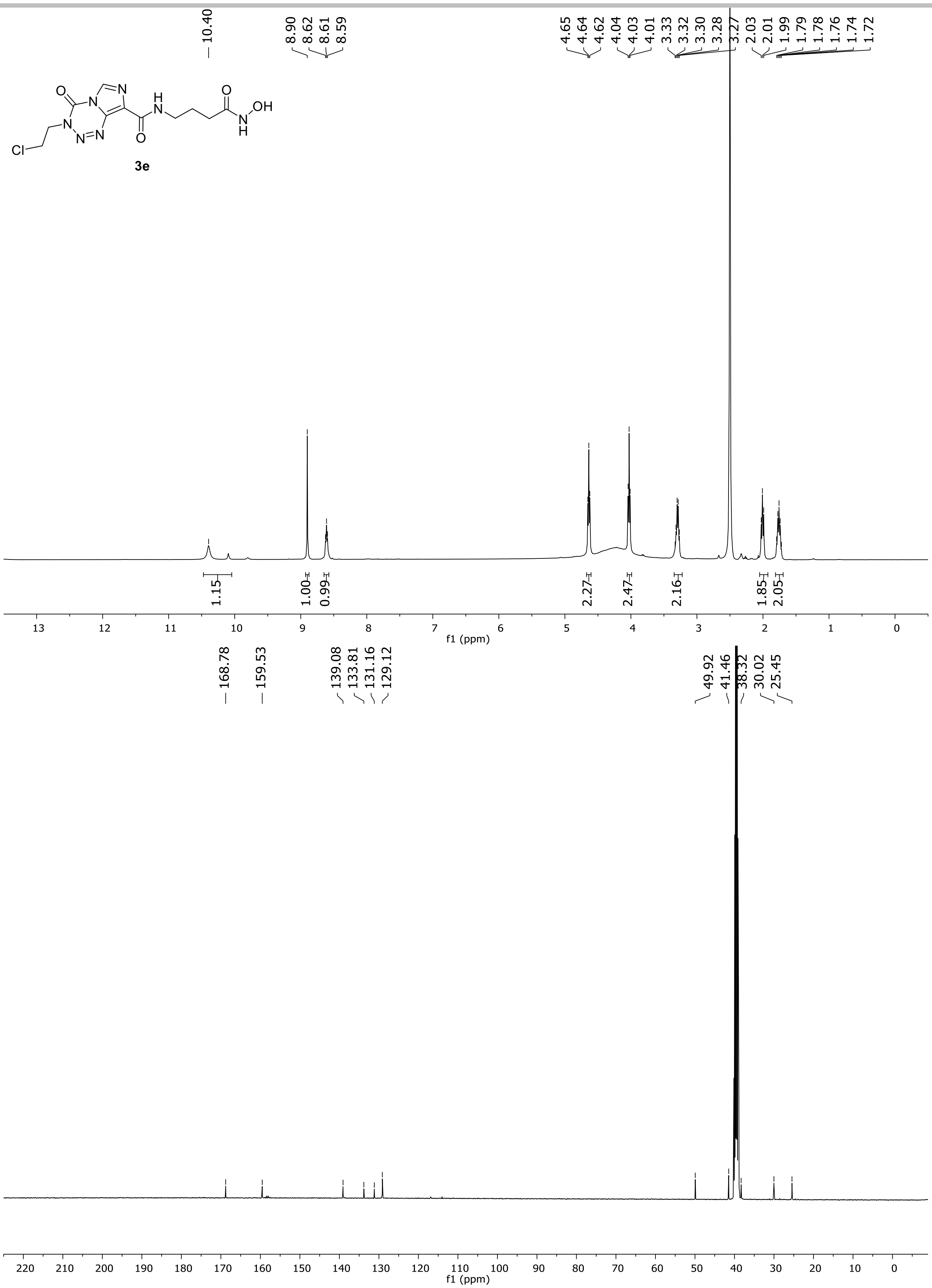

Figure S10: ^{1}H and ^{13}C-NMR spectra of **3e** in DMSO-d_6.

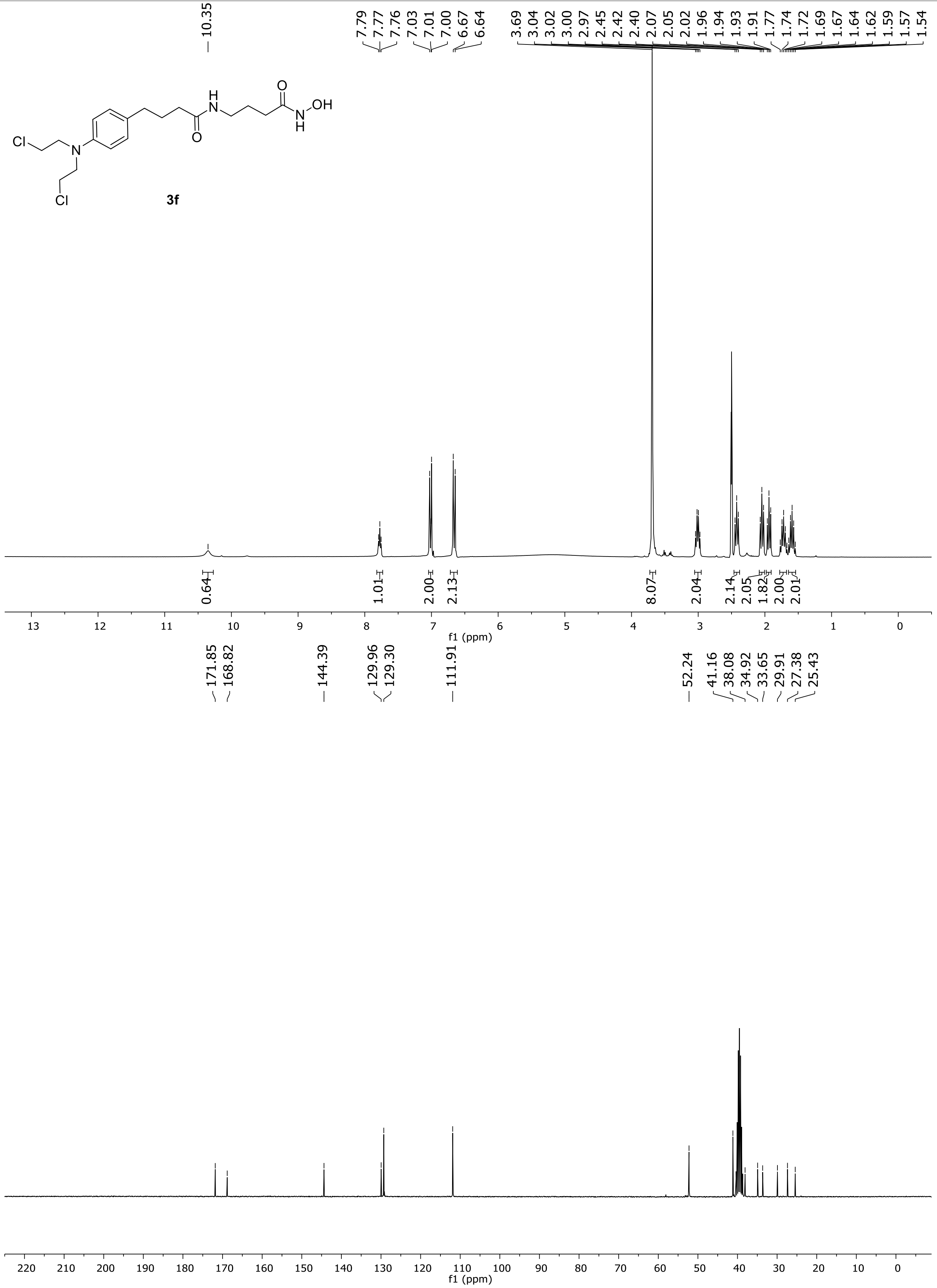

Figure S11: ^{1}H and ^{13}C-NMR spectra of **3f** in DMSO-d_6.

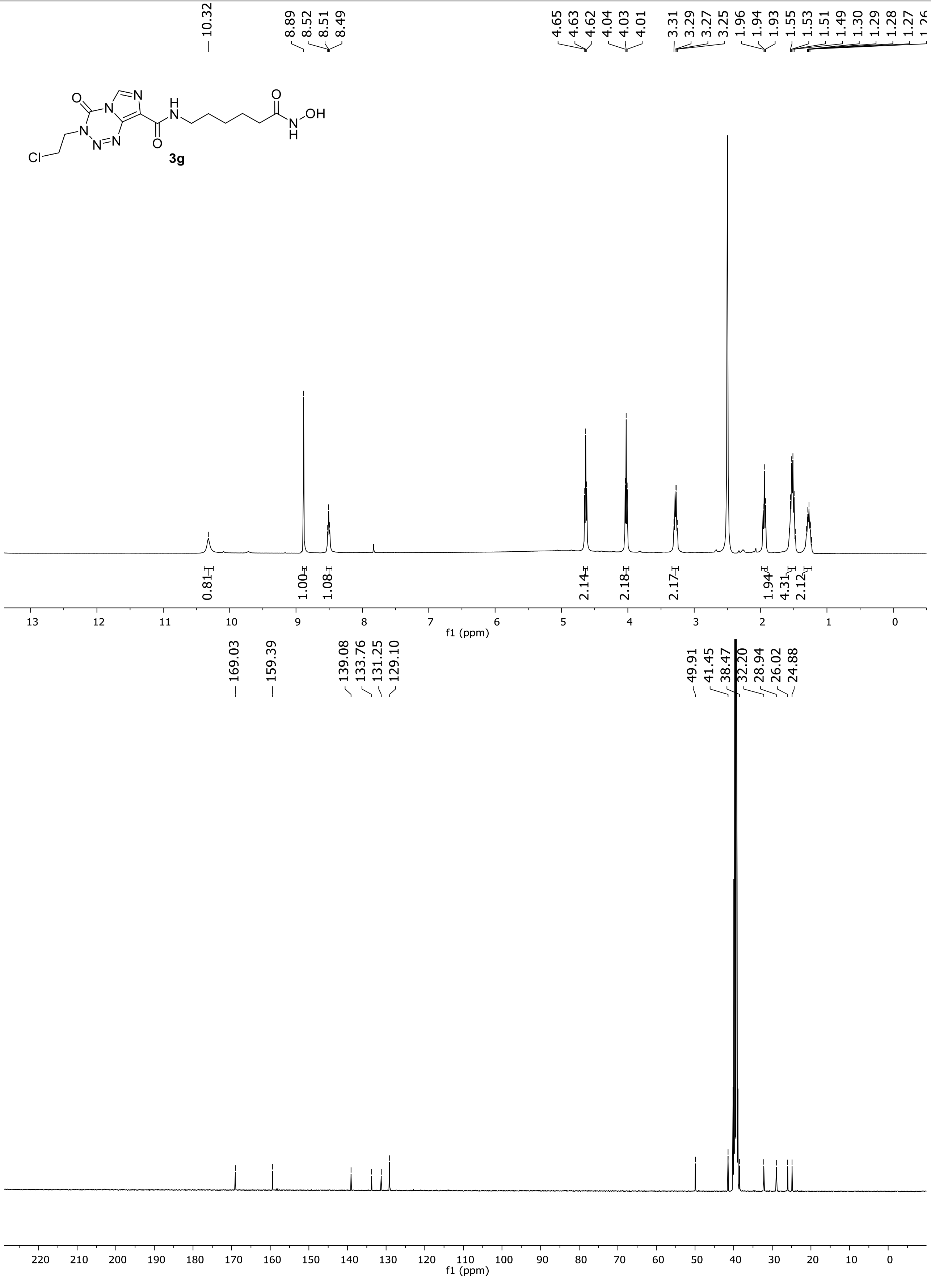

Figure S12: ^{1}H and ^{13}C-NMR spectra of **3g** in DMSO-d_6.

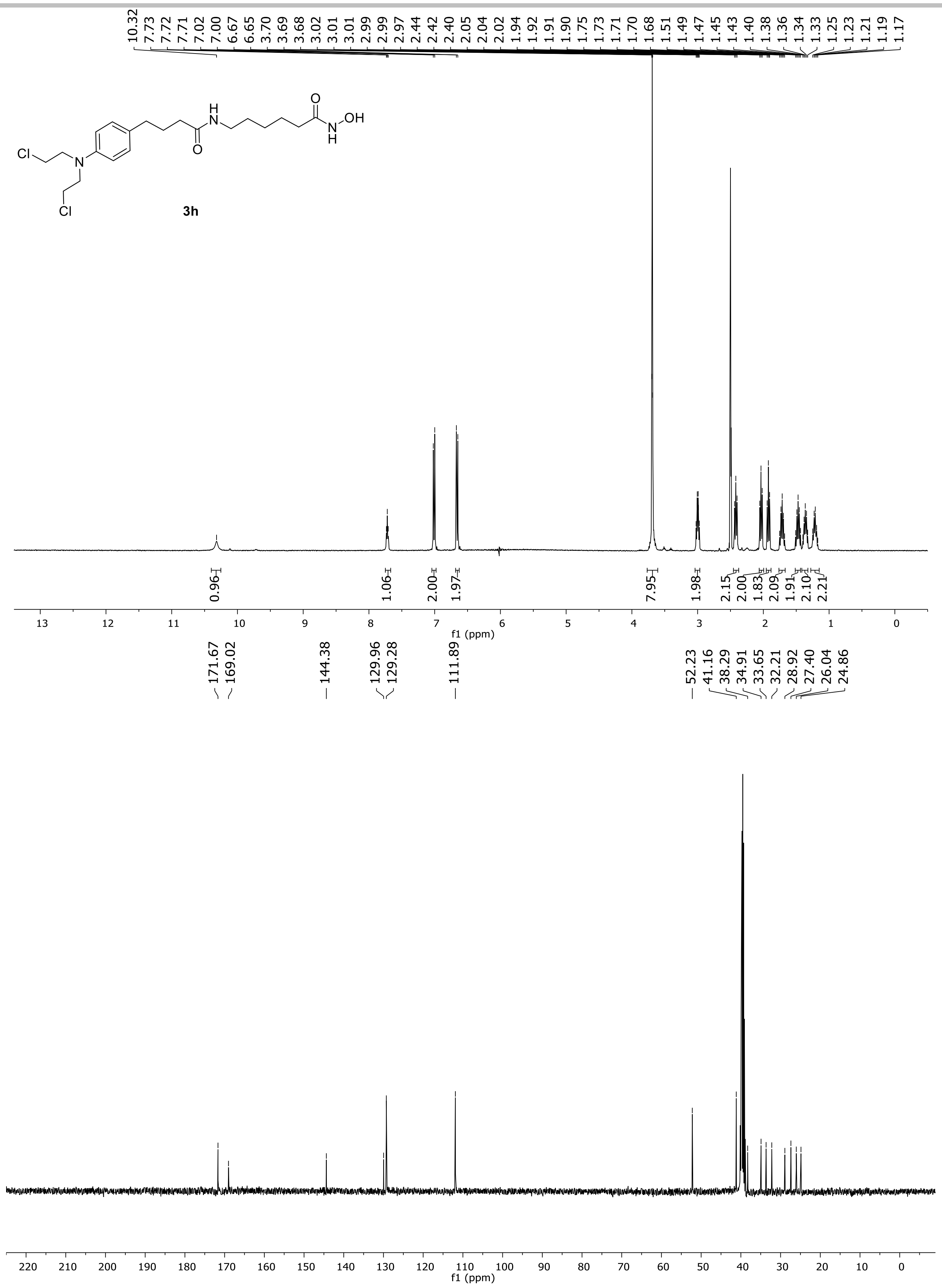

Figure S13: ^{1}H and ^{13}C-NMR spectra of **3h** in DMSO-*d*$_6$.

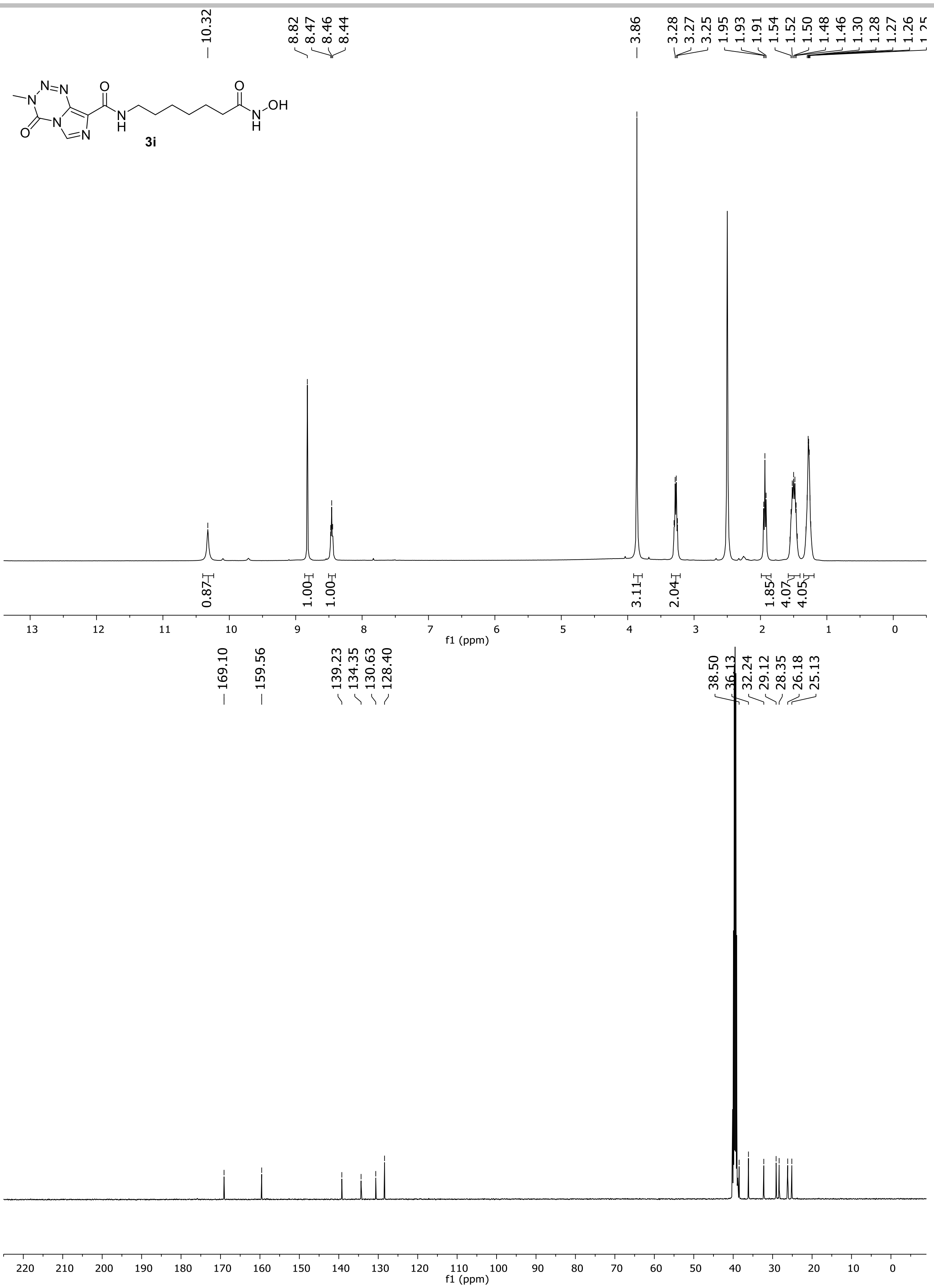

Figure S14: ^{1}H and ^{13}C-NMR spectra of **3i** in DMSO-*d*$_6$.

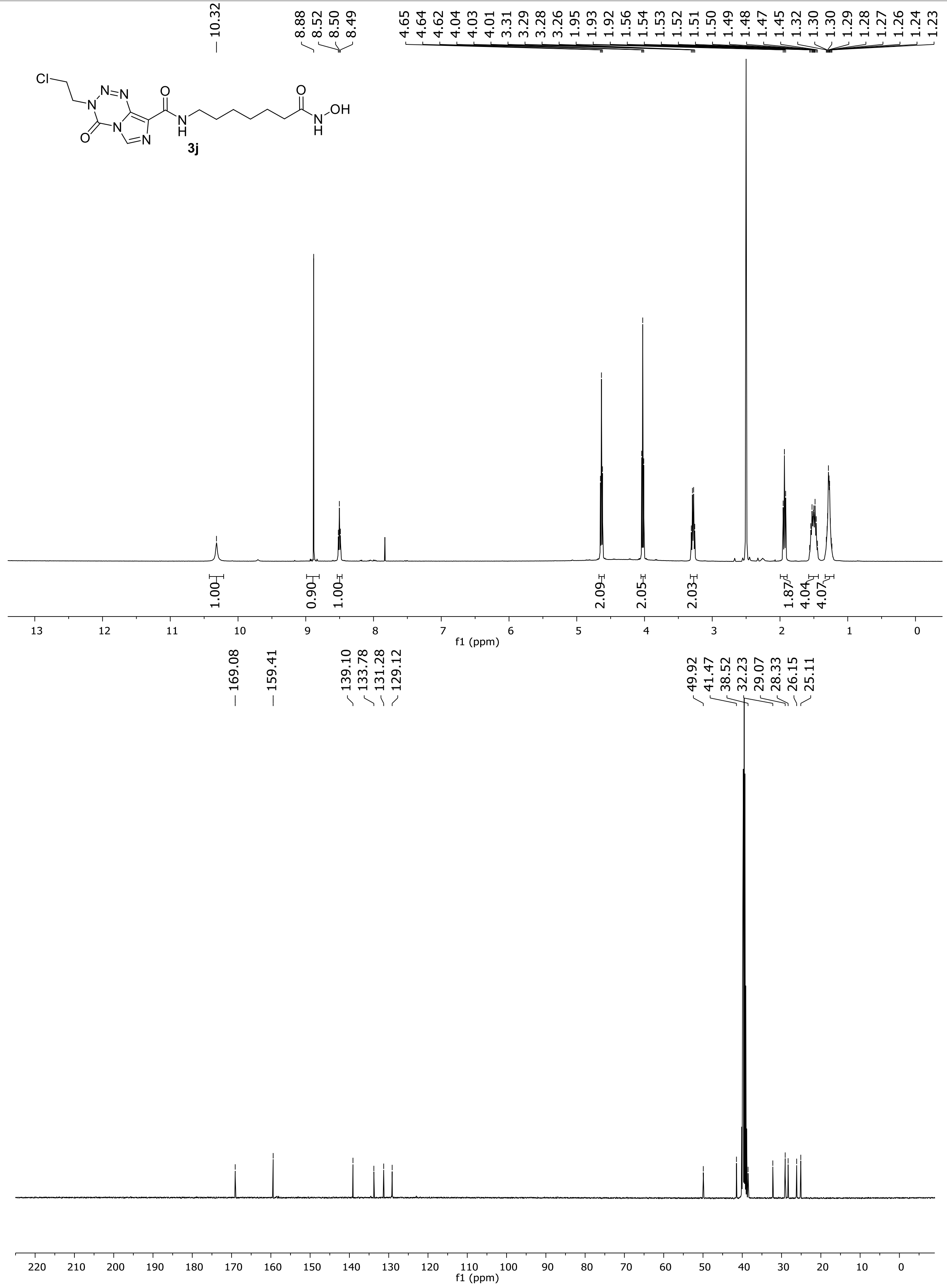

Figure S15: ^{1}H and ^{13}C-NMR spectra of **3j** in DMSO-d_6.

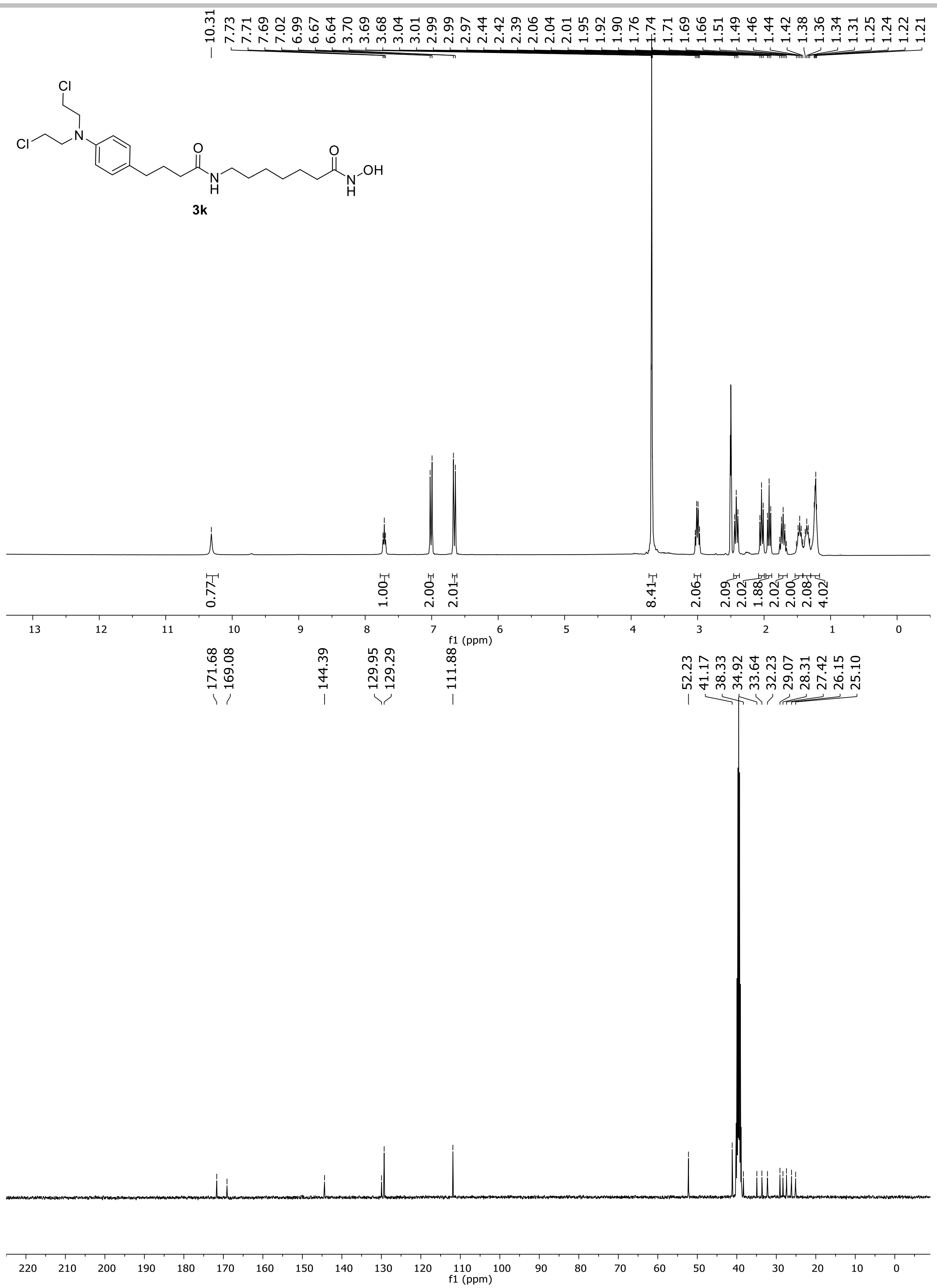

Figure S16: ^{1}H and ^{13}C-NMR spectra of **3k** in DMSO-d_6.

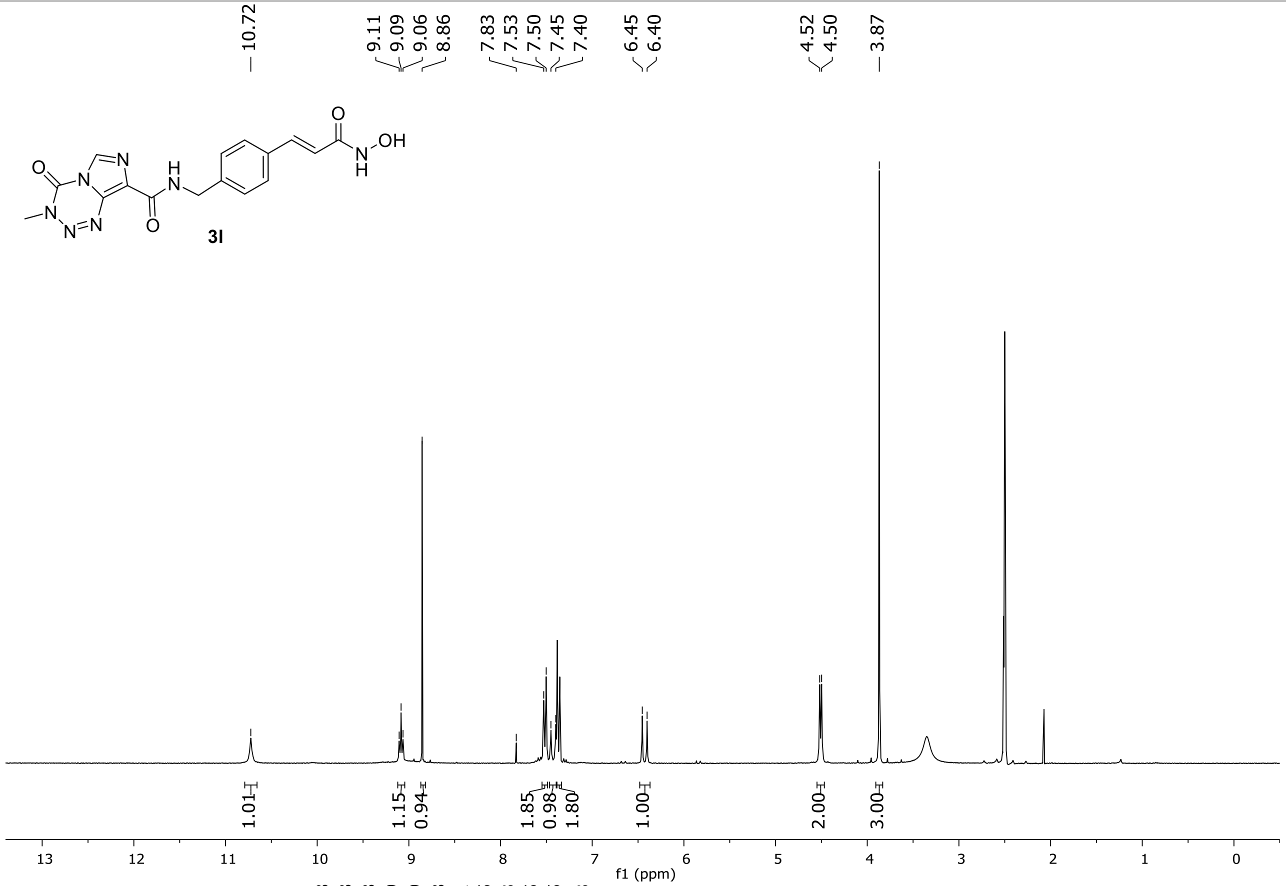

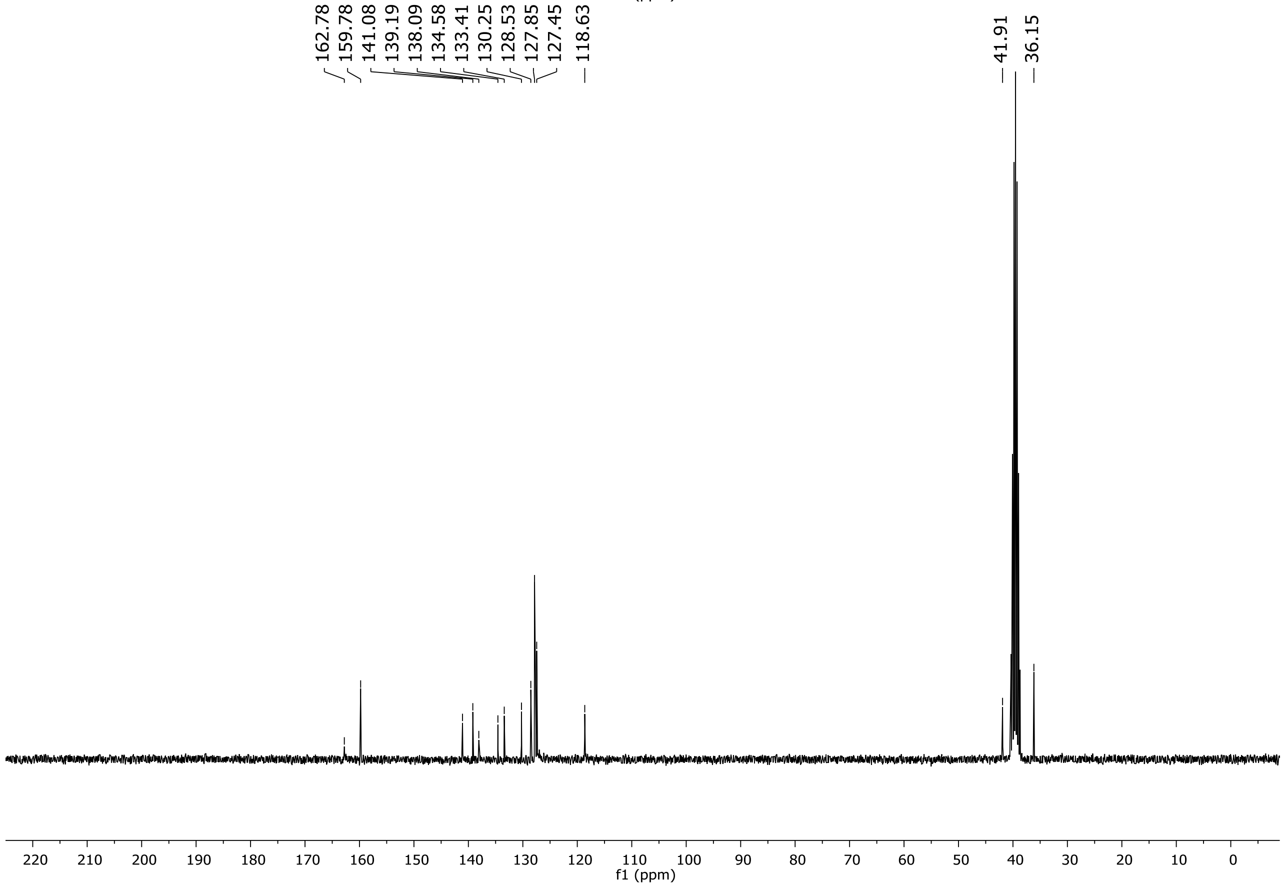

Figure S17: ^{1}H and ^{13}C-NMR spectra of **3l** in DMSO-d_6.

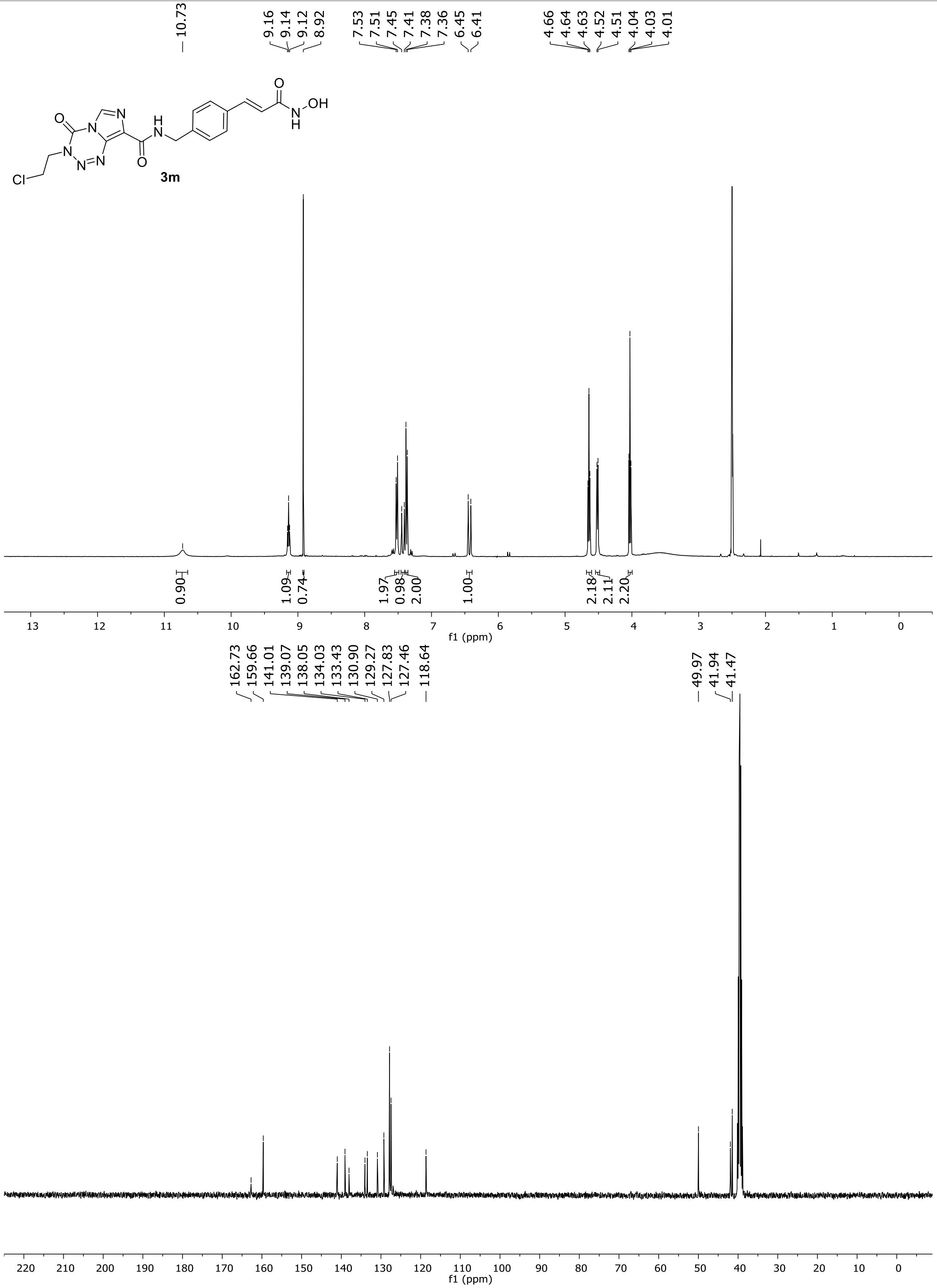

Figure S18: ^{1}H and ^{13}C-NMR spectra of **3m** in DMSO-d_6.

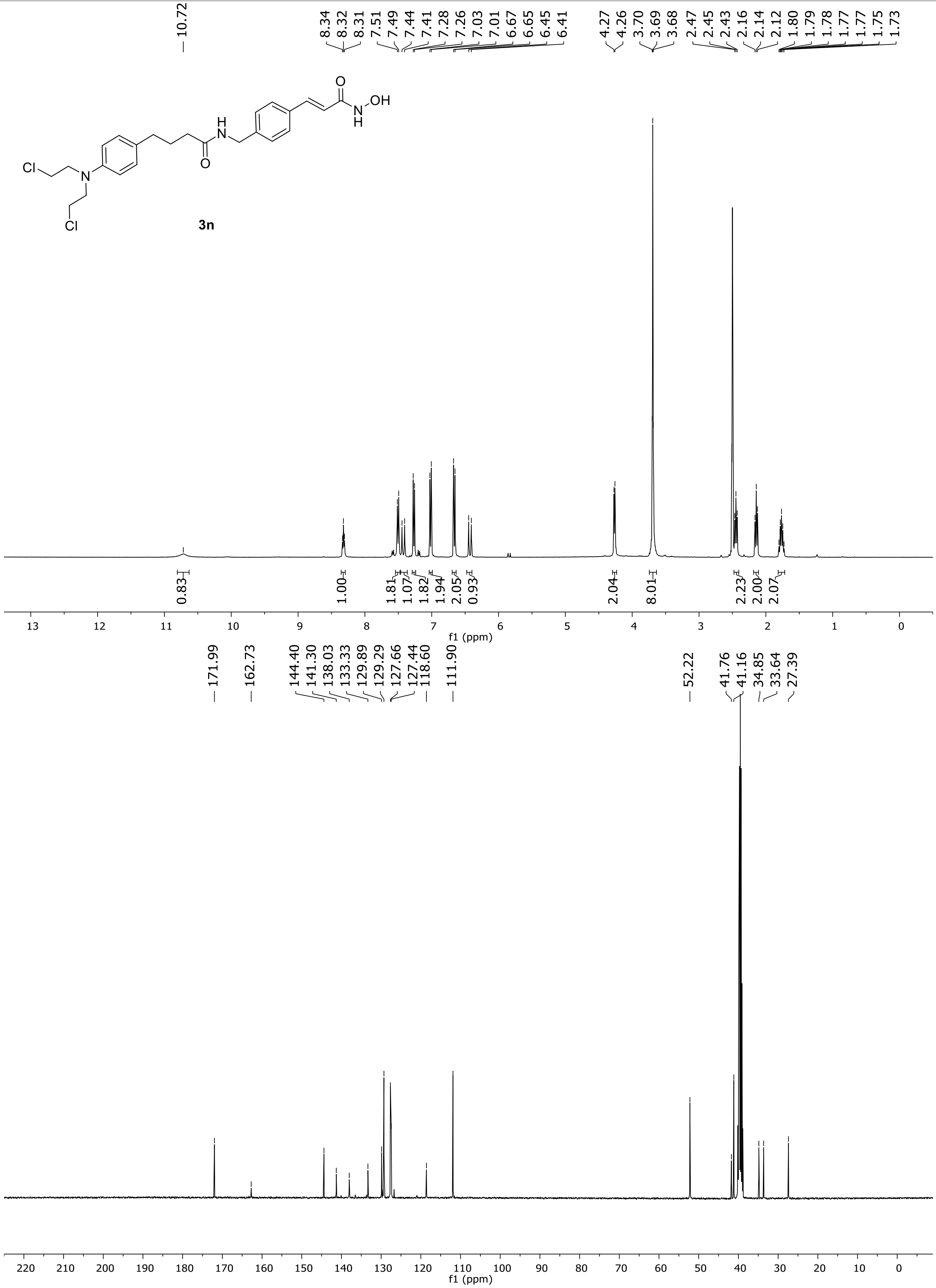

Figure S19: ^{1}H and ^{13}C-NMR spectra of **3n** in DMSO-d_6.

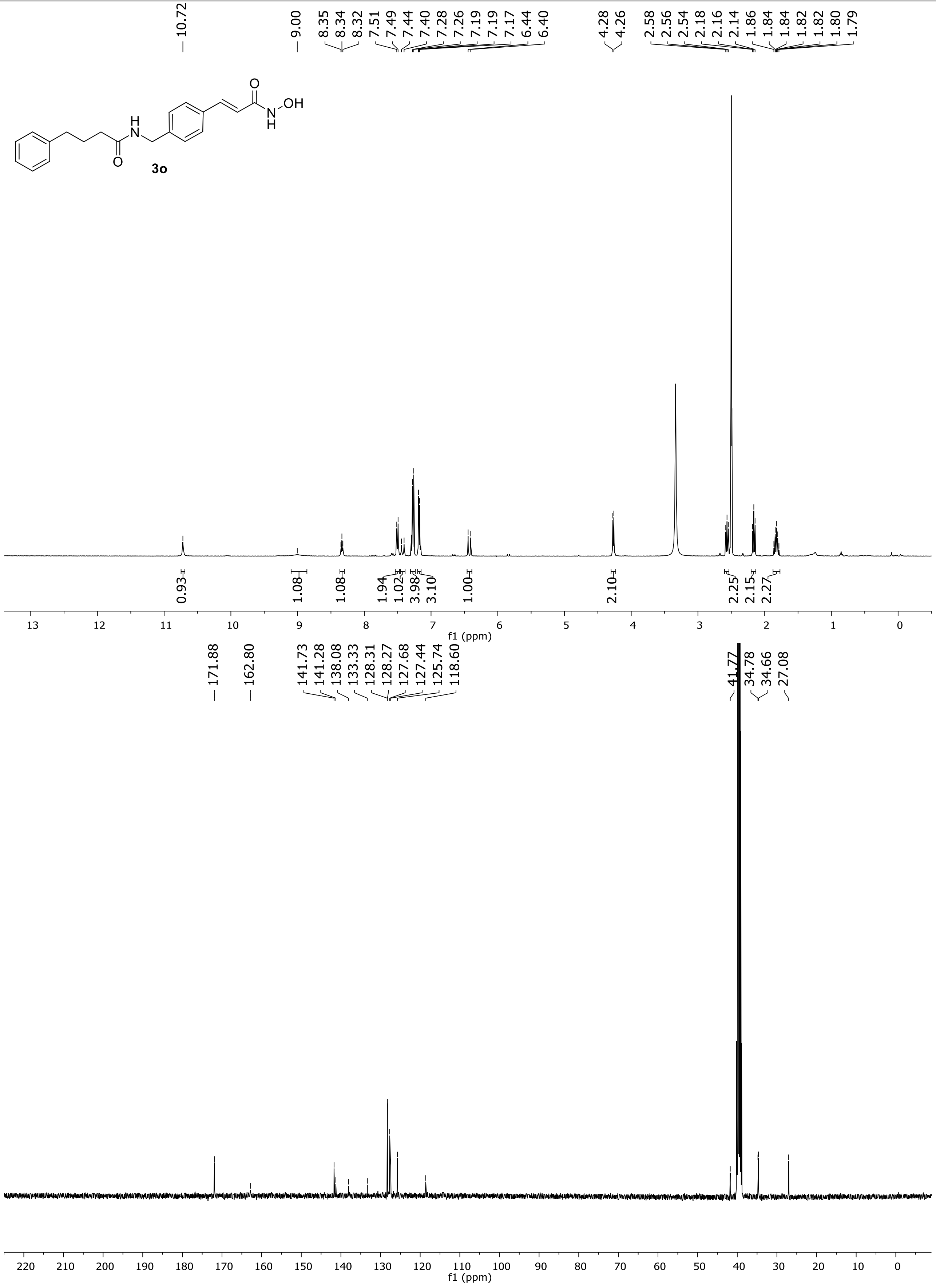

Figure S20: ^{1}H and ^{13}C-NMR spectra of **3o** in DMSO-d_6.

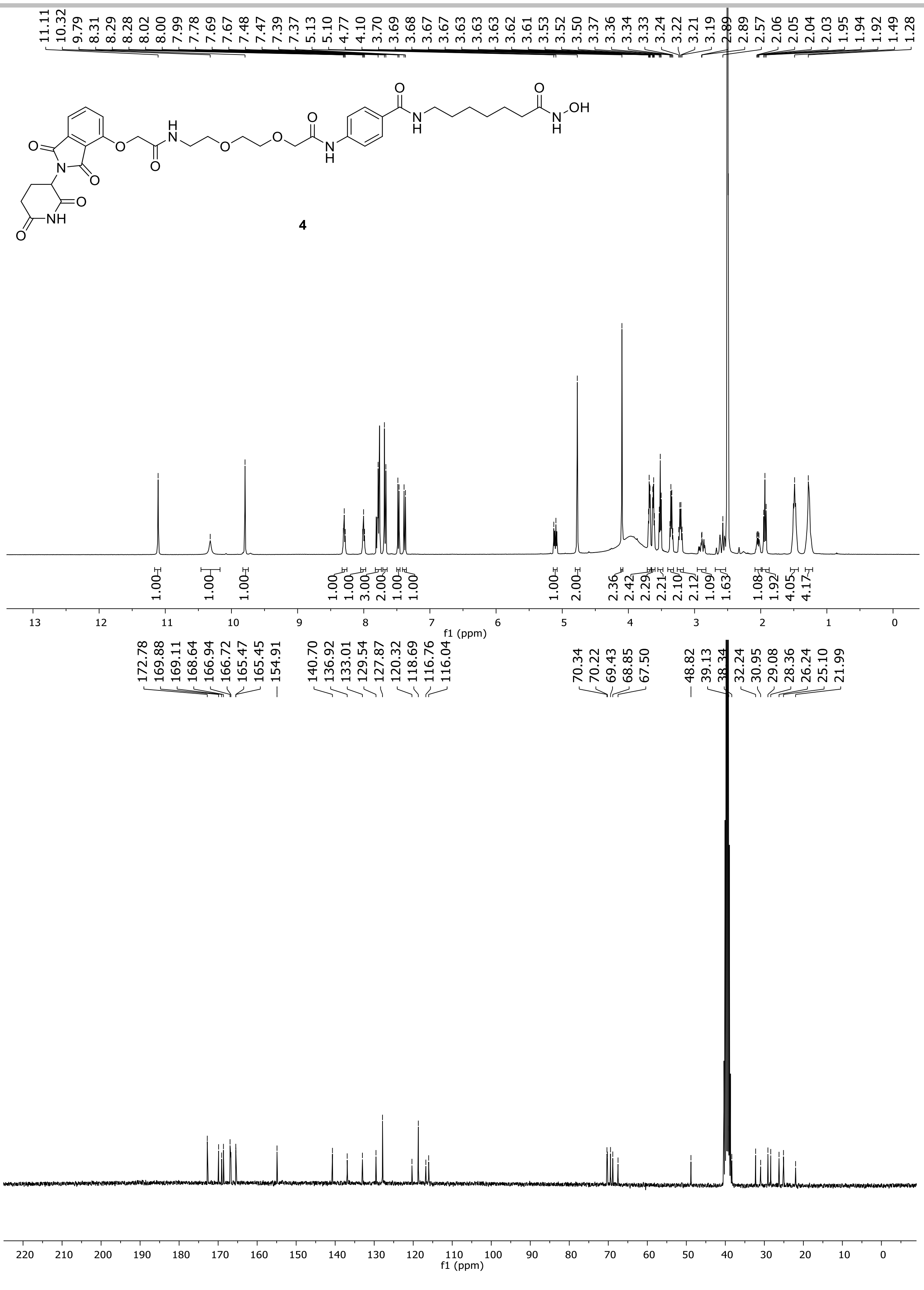

Figure S21: ^{1}H and ^{13}C-NMR spectra of **4** in DMSO-d_6.

3 Biological Evaluation

3.1 Reagents

Cisplatin was purchased from Sigma (München, Germany) and dissolved in 0.9% sodium chloride solution, propidium iodide (PI) was purchased from PromoKine (Heidelberg, Germany). Stock solutions (10 mM) of vorinostat, nexturastat A (Selleckchem, Houston, Texas, USA), chlorambucil (Sigma Aldrich, Germany), temozolomide (TCI Chemicals) and mitozolomide (synthesized, see compound **12**), were prepared with DMSO and diluted to the desired concentrations with DMEM. All other reagents were supplied by PAN Biotech (Aidenbach, Germany) unless otherwise stated.

3.2 Cell Lines and Cell Culture

The human tongue squamous cell carcinoma cell line Cal27 and the human primary glioblastoma cell lines U87 and U251 were obtained from Merck KGaA (Darmstadt, Germany). All cell lines were grown at 37 °C under humidified air supplemental with 5 % CO_2 in DMEM containing 10% heat inactivated fetal calf serum, 120 IU/mL penicillin, and 120 µg/mL streptomycin. The cells were grown to 80 % confluency before being used in further assays.

3.3 MTT Cell Viability Assay

The rate of cell survival under the action of test compounds was evaluated by an improved MTT assay as previously described.[9-11] Briefly, cells were plated out (Cal27 2,500 c/w, U87 4,000 c/w, and U251 2,500 c/w) and incubated with the compounds in different concentrations. After 72 hours, the incubation was ended by addition of MTT (Serva, Heidelberg, Germany) solution (5 mg/mL in PBS). The formazan precipitate was dissolved in DMSO (VWR, Langenfeld, Germany). Absorbance was measured at 544 nm and 690 nm in a FLUOstar microplate-reader (BMG LabTech, Offenburg, Germany).

3.4 γ-H2AX Expression DNA Damage Assay

The γ-H2AX DNA damage assay was based on an assay published by Ziegler et al. with minor modifications.[12] Briefly, Cal27 cells were seeded in 96-well tissue culture plates (Corning, Kaiserslautern, Germany) at a density of 2,500 c/w. Cells were treated with indicated concentrations and compounds for 24 hours. Cells were fixed with 4 % formaldehyde solution followed by incubation with ice-cold methanol and blocking in blocking buffer (5 % BSA, 0.3 % Triton X-100 in PBS). Cells were incubated with primary antibody (Cat. No. 05-636, Merck Millipore, Darmstadt, Germany) overnight at 4°C and secondary fluorophore-labeled antibody (Northern Light Anti Mouse, Cat. No. NL007, Biotechne, Wiesbaden, Germany) for 2 hours in the dark. Cell nuclei were counterstained with Hoechst 33342. Analysis was performed with Thermo Fischer ArrayScan XTI (Thermo Scientific, Wesel, Germany).

3.5 Measurement of Apoptotic Nuclei

Cal27 cells were seeded at a density of 32,000 c/w in 24-well plates (Sarstedt, Nürnbrecht, Germany). Cells were treated with indicated compounds for 24 hours. Supernatant was removed after a centrifugation step and the cells were lysed in 500 µL hypotonic lysis buffer (0.1 % sodium citrate, 0.1 % Triton X-100, 100 µg/mL propidium iodide) at 4°C overnight in the dark. The percentage of apoptotic nuclei with DNA content in sub-G1 was analyzed by flow cytometry using the CyFlow instrument (Partec, Norderstedt, Germany).

3.6 Caspase 3/7 Activation Assay

Compound-induced activation of caspases 3 and 7 was analyzed using the CellEvent Caspase-3/7 green detection reagent (Thermo Scientific, Wesel, Germany) according to the manufacturer´s instructions. Briefly, Cal27 cells were seeded in 96-well plates (Corning, Kaiserslautern, Germany) at a density of 2,500 c/w. Cells were treated with indicated compounds for 24 hours. Then, medium was removed and 50 µL of CellEvent Caspase 3/7 green detection reagent (2.0 µM in PBS supplemented with 5 % heat inactivated FBS) was added. Cells were incubated for 30 min at 37 °C in a humidified incubator before imaging by using the *Thermo Fisher* ArrayScan XTI high content screening (HCS) system with a 10X magnification (Thermo Scientific). Hoechst 33342 was used for nuclei staining. The pan caspase inhibitor QVD was used in a concentration of 20 µM diluted in DMEM and incubated 30 min prior to compound addition.

3.7 Immunoblotting

Cells were treated with the indicated concentration of the compound or vehicle (DMSO) for 48 hours. Cell pellets were dissolved with RIPA buffer (50 mM Tris-HCl pH 8.0, 1 % Triton X-100, 0.5 % sodium deoxycholate, 0.1% SDS, 150 mM sodium chloride, 2 mM EDTA, supplemented with protease and phosphatase inhibitors (Pierce protease and phosphatase inhibitor mini tablets, Thermo Scientific, Wesel, Germany) and clarified by centrifugation. Equal amount of total protein were resolved by SDS-PAGE and transferred to polyvinylidene fluoride membranes (Merck Millipore, Darmstadt, Germany). PageRuler Prestained Protein Ladder, 10 to 180 kDa (Thermofisher Scientific, Wesel, Germany) was used as protein molecular weight marker. Blots were incubated with antibodies against acetylated α-tubulin (Cat. No. #5335), HDAC6 (Cat. No. #7558), HDAC1 (Cat. No. #2062), acetyl histone H3 (Cat. No. # 9677) and GAPDH (Cat. No. #97166), obtained from Cell Signaling Technology, Danvers, MA.

3.8 Data Analysis

Concentration-effect curves were constructed with Prism 7.0 (GraphPad, San Diego, CA, USA) by fitting the pooled data of at least three experiments performed in triplicates to the four-parameter logistic equation. Statistical analysis was performed using t-test or one-way ANOVA. To analyze the superadditive effects of chlorambucil and **3o** on caspase3/7-activation and DNA damage, the values of single treatments were summed up and the standard deviation calculated according to Bandolik et al.[11] This value was compared with the effects of **3n** using t-test.

3.9 In-vitro human HDAC1/2/3/6 assay

All reactions were performed in OptiPlate-96 black microplates (Perkin Elmer) with duplicate series in at least two independent experiments. An assay end volume of 50 µL was set for each experiment and all reactions were carried out in assay buffer (50 mM Tris-HCl, pH 8.0, 137 mM NaCl, 2.7 mM KCl, 1.0 mM $MgCl_2$, 0.1 mg/mL BSA) with appropriate concentrations of substrate and inhibitors obtained by dilution from 10 mM stock solutions. Control wells without enzyme and control wells with vorinostat as reference were included in each plate. After addition of 5 µL test compound or control in assay buffer, 35 µL of the fluorogenic substrate ZMAL (21.43 µM in assay buffer) and 10 µL of human recombinant HDAC1 (1.2 ng/µL in assay buffer; BPS Bioscience, Catalog# 50051) or HDAC2 (1.0 ng/µL, BPS Bioscience Catalog# 50052) or HDAC3 (1.0 ng/µL, BPS Bioscience Catalog# 50003) or HDAC6 (2.0 ng/µL in assay buffer; BPS Bioscience, Catalog# 50006) the reaction were incubated at 37 °C for 90 min. Afterwards 50 µL of 0.4 mg/mL trypsin in trypsin buffer (50 mM Tris-HCl, pH 8.0, 100 mM NaCl) was added, followed by further incubation at 37 °C for 30 min. Plates were analyzed using a Fluoroskan Ascent microplate reader (Thermo Scientific) and fluorescence was measured with an excitation wavelength of 355 nm and an emission wavelength of 460 nm.

3.10 In-vitro human HDAC8 assay

HDAC8 recombinant enzyme was purchased from Reaction Biology Corp. (Cat Nr. KDA-21-481; Malvern, PA, USA). The HDAC activity assay was performed in 96-well-plates (Corning, Kaiserslautern, Germany). Briefly 20 ng of HDAC8 per reaction were used. HDAC8 was diluted in assay buffer (50 mM Tris-HCl, pH 8.0, 137 mM NaCl, 2.7 mM KCl, 1.0 mM $MgCl_2$, and 1.0 mg/mL BSA). After a 5 min incubation step the reaction was started with 10 µL of 60 µM Boc-Lys-(TFa)-AMC (Bachem, Bubendorf, Switzerland). The reaction was stopped after 90 min by adding 100 µL stop solution (16 mg/mL trypsin, 2.0 µM panobinostat in 50 mM Tris-HCl, pH 8.0, and 100 mM NaCl). 15 min after the addition of the stop solution the fluorescence intensity was measured at excitation of 355 nm and emission of 460 nm in a NOVOstar microplatereader (BMG LabTech, Offenburg, Germany).

4. Docking Studies

Docking of 3n and 3o to human HDAC6 with RosettaLigand

The crystal structure of human HDAC6 (PDB: 5EDU)[13] was obtained from the Protein Data Bank (PDB, www.rcsb.org). Residues 114-478 of chain A corresponding to the maltose-binding periplasmatic protein, which was used to facilitate crystallization, and a second copy of the protein in the crystallographic unit, chain B, were deleted. All heteroatom records were removed, except for all metal ions (one zinc atom and two potassium atoms). The zinc bound water was positioned based on crystal structure PDB: 6CW8.[14] Throughout the calculation a metal ion restraint was applied. The structure was optimized to the closest local energy minimum using the RosettaRelax application with coordinate constraints on the backbone.[15] Ligand input files for **3n** and **3o** were created with ChemDraw. An initial 3D conformer with hydrogen atoms was constructed in Corina[16] and subsequently an ensemble of 1000 low-energy conformers was produced by using the BCL:ConformerGenerator.[17] One conformer was placed in the binding pocket of HDAC6 and aligned to the known binding pose of hydroxamic acids in PDB: 6CW8.[11] A constraint file was constructed using the known distance measures between hydroxamic acid, zinc ion and water molecule as described by Porter *et al*.[18] Ligand docking was performed for an initial 10,000 models and subsequently three rounds of focused refinement using RosettaLigand were applied to identify the best scoring models by predicted binding energy.[19-21] A final ensemble of models was selected for **3n** from three clusters containing 640 models and for **3o** from seven clusters containing 315 models of ligand poses by root mean square deviation. Only such models were considered that complied with the observed binding pose of the hydroxamic acid residue and the hydrogen bond between the amide group and serine 531, a known interaction of this compound class.[14,18]

The following commands were executed throughout the modeling process.

Relax input starting structures:

```
/dors/meilerlab/apps/rosetta/rosetta-3.11/main/source/bin/relax.default.linuxgccrelease -s
5edu_align_A.pdb -database /dors/meilerlab/apps/rosetta/rosetta-3.11/main/database/ -
in:auto_setup_metals -constrain_relax_to_start_coords -ignore_waters False -out:prefix
relax_ -nstruct 25
```

Options for RosettaLigand:

```
-packing
    -ex1
    -ex2
    -no_optH false
    -flip_HNQ true
    -ignore_ligand_chi true

-parser
    -protocol
/dors/meilerlab/home/schoedct/Documents/Versuche/HDAC_docking_chlorambucil_derivates/dock_to
_human_HDAC6/docking/docking-LOST/dock.xml

-mistakes
    -restore_pre_talaris_2013_behavior true

-constraints:cst_file
/dors/meilerlab/home/schoedct/Documents/Versuche/HDAC_docking_chlorambucil_derivates/dock_to
_human_HDAC6/docking/docking-LOST/constraint.cst
```

RosettaScripts protocol for executing RosettaLigand:

```
<ROSETTASCRIPTS>

        <SCOREFXNS>
            <ScoreFunction name="ligand_soft_rep" weights="ligand_soft_rep">
            </ScoreFunction>
            <ScoreFunction name="hard_rep" weights="ligand">
            </ScoreFunction>
        </SCOREFXN  S>

        <LIGAND_AREAS>
            <LigandArea name="inhibitor_dock_sc" chain="X" cutoff="6.0" add_nbr_radius="true"
all_atom_mode="false"/>
```

```
            <LigandArea name="inhibitor_final_sc" chain="X" cutoff="6.0" add_nbr_radius="true"
all_atom_mode="false"/>
            <LigandArea name="inhibitor_final_bb" chain="X" cutoff="7.0"
add_nbr_radius="false" all_atom_mode="true" Calpha_restraints="0.3"/>
        </LIGAND_AREAS>

        <INTERFACE_BUILDERS>
            <InterfaceBuilder name="side_chain_for_docking"
ligand_areas="inhibitor_dock_sc"/>
            <InterfaceBuilder name="side_chain_for_final" ligand_areas="inhibitor_final_sc"/>
            <InterfaceBuilder name="backbone" ligand_areas="inhibitor_final_bb"
extension_window="3"/>
        </INTERFACE_BUILDERS>

        <MOVEMAP_BUILDERS>
            <MoveMapBuilder name="docking" sc_interface="side_chain_for_docking"
minimize_water="false"/>
            <MoveMapBuilder name="final" sc_interface="side_chain_for_final"
bb_interface="backbone" minimize_water="false"/>
        </MOVEMAP_BUILDERS>

        <SCORINGGRIDS ligand_chain="X" width="25">
            <ClassicGrid grid_name="classic" weight="1.0"/>
        </SCORINGGRIDS>

        <MOVERS>
            <ConstraintSetMover name="coordinate" add_constraints="true"
cst_file="/dors/meilerlab/home/schoedct/Documents/Versuche/HDAC_docking_chlorambucil_derivat
es/dock_to_human_HDAC6/docking/docking-LOST/constraint.cst"/>
            <Transform name="transform" chain="X" box_size="7.0" move_distance="0.2" angle="20"
cycles="500" repeats="1" temperature="5"/>
            <HighResDocker name="high_res_docker" cycles="6" repack_every_Nth="3"
scorefxn="ligand_soft_rep" movemap_builder="docking"/>
            <FinalMinimizer name="final" scorefxn="hard_rep" movemap_builder="final"/>
            <InterfaceScoreCalculator name="add_scores" chains="X" scorefxn="hard_rep" />
        </MOVERS>

        <PROTOCOLS>
            <Add mover_name="coordinate"/>
            <Add mover_name="transform"/>
            <Add mover_name="high_res_docker"/>
            <Add mover_name="final"/>
            <Add mover_name="add_scores"/>
        </PROTOCOLS>

</ROSETTASCRIPTS>
```

Executing RosettaLigand:

```
#!/bin/tcsh

 foreach a (`cat templates.ls`)
     /dors/meilerlab/apps/rosetta/rosetta-
3.10/main/source/bin/rosetta_scripts.default.linuxgccrelease -s $a -database
/dors/meilerlab/apps/rosetta/rosetta-3.10/main/database/ -ignore_waters False -
in:auto_setup_metals
@/dors/meilerlab/home/schoedct/Documents/Versuche/HDAC_docking_chlorambucil_derivates/dock_t
o_human_HDAC6/docking/docking-LOST/docking.options -in:file:extra_res_fa
/dors/meilerlab/home/schoedct/Documents/Versuche/HDAC_docking_chlorambucil_derivates/dock_to
_human_HDAC6/docking/docking-LOST/Los.params -out:prefix dock_ -nstruct 400 -out:path:all
/dors/meilerlab/home/schoedct/Documents/Versuche/HDAC_docking_chlorambucil_derivates/dock_to
_human_HDAC6/docking/docking-LOST/output_run_01/ &
end
```

Calculation of binding energy

```
/dors/meilerlab/apps/rosetta/rosetta-
3.10/main/source/bin/rosetta_scripts.default.linuxgccrelease -l run_01_models.ls -database
/dors/meilerlab/apps/rosetta/rosetta-3.10/main/database/ -in:file:extra_res_fa nLo.params -
parser:protocol interface.xml -ignore_waters False -in:auto_setup_metals -
out:file:score_only
```

RMSD versus binding energy (interface delta) plots for **3n** and **3o**

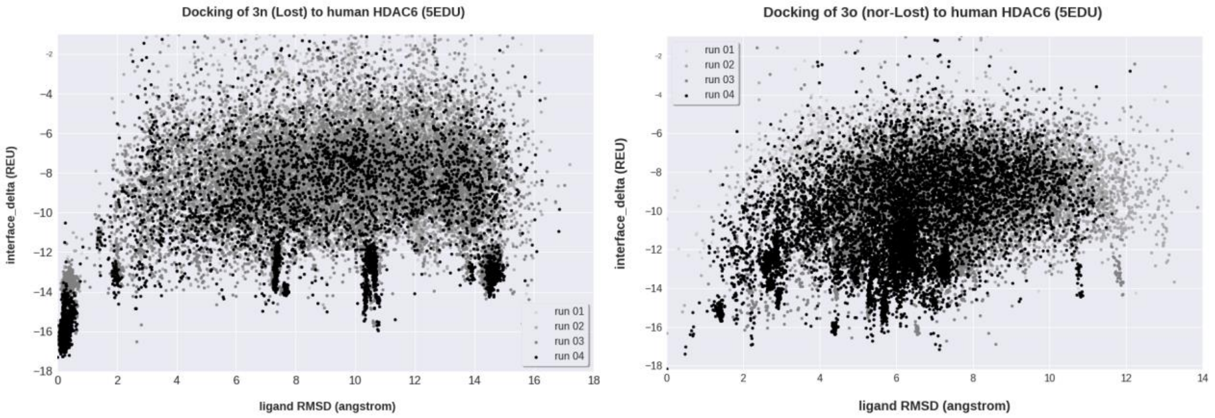

Figure S22: Ligand RMSD versus interface_delta plots for **3n** and **3o**. Both compounds were docked over four consecutive rounds, always taking the best 1000 models by interface_delta forward and redocking the ligand.

References

[1] J. Lohbeck, A. Miller, *Bioorg Med Chem Lett.* **2016**, *21*, 5260-5262.
[2] T.-C. Chou, *Cancer Res*. **2010**, 70, 440-446.
[3] T.-C. Chou, P. Talalay, *Adv. Enzyme Regul*. **1984**, 22, 27-55.
[4] S. I. Khan, M. W. Grinstaff, *Tetrahedron Lett.* **1998**, *39*, 8031-8034.
[5] H. Aloysius, L. Hu, *Chem Biol Drug Des* **2015**, *4*, 837-848.
[6] H. Seyler, A. Kilbinger, *Tetrahedron Lett.* **2013**, *8*, 753-756.
[7] J. F. Shealy, R. F. Struck, L. B. Holum, J. A. Montgomery, *J. Org. Chem.* **1961**, *26*, 7, 2396-2401.
[8] K. R. Horspool, M. F. G. Stevens, C. G. Newton, E. Lunt, R. J. A. Walsh, B. L. Pedgrift, G. U. Baig, F. Lavelle, C. Fizames, *J. Med. Chem* **1990**, 33, 1393-1399.
[9] L. Marek, A. Hamacher, F. K. Hansen, K. Kuna, H. Gohlke, M. U. Kassack, T. Kurz, *J. Med. Chem.* **2013**, *56*, 427-436.
[10] L. H. Engelke, A. Hamacher, P. Proksch, M. U. Kassack, *J. Cancer.* **2016**, *7*, 353-363.
[11] J. J. Bandolik, A. Hamacher, C. Schrenk, R. Weishaupt, M. U. Kassack, *Int J Mol Sci.* **2019**, *20*, 3052.
[12] V. Ziegler, A. Albers, G. Fritz, *Biochim Biophys Acta.* **2016**, *1863*, 1082-1092.
[13] Y. Hai, D. W. Christianson, *Nat Chem Biol* **2016,** *12*, 741-747.
[14] S. Bhatia, V. Krieger, M. Groll, J. D. Osko, N. Ressing, H. Ahlert, A. Borkhardt, T. Kurz, D. W. Christianson, J. Hauer, F. K. Hansen, *J Med Chem* **2018,** *61*, 10299-10309.
[15] L. G. Nivon, R. Moretti, D. A. Baker, *PLoS One* **2013,** *8*, e59004.
[16] C. Schwab, *Drug Discov Today Technol* **2010,** *7*, e203-70.
[17] S. Kothiwale, J. L. Mendenhall, J. Meiler, *J Cheminform* **2015,** *7*, 47.
[18] N. J. Porter, J. D. Osko, D. Diedrich, T. Kurz, J. M. Hooker, F. K. Hansen, D. W. Christianson, *J Med Chem* **2018,** *61* (17), 8054-8060.
[19] S. DeLuca, K. Khar, J. Meiler, *PLoS One* **2015,** *10* (7), e0132508.
[20] K. W. Kaufmann, J. Meiler, *PLoS One* **2012,** *7* (12), e50769.
[21] J. Meiler, D. Baker, *Proteins* **2006,** *65*, 538-548.
